# Comparative mapping of crawling-cell morphodynamics in deep learning-based feature space

**DOI:** 10.1371/journal.pcbi.1009237

**Published:** 2021-08-12

**Authors:** Daisuke Imoto, Nen Saito, Akihiko Nakajima, Gen Honda, Motohiko Ishida, Toyoko Sugita, Sayaka Ishihara, Koko Katagiri, Chika Okimura, Yoshiaki Iwadate, Satoshi Sawai

**Affiliations:** 1 Department of Basic Science, Graduate School of Arts and Sciences, University of Tokyo, Tokyo, Japan; 2 Universal Biological Institute, University of Tokyo, Tokyo, Japan; 3 Exploratory Research Center on Life and Living Systems, National Institutes of Natural Sciences, Okazaki, Japan; 4 Research Center for Complex Systems Biology, Graduate School of Arts and Sciences, University of Tokyo, Tokyo, Japan; 5 Department of Biosciences, School of Science, Kitasato University, Sagamihara, Japan; 6 Faculty of Science, Yamaguchi University, Yamaguchi, Japan; 7 Department of Biology, Graduate School of Science, University of Tokyo, Tokyo, Japan; Inria, FRANCE

## Abstract

Navigation of fast migrating cells such as amoeba *Dictyostelium* and immune cells are tightly associated with their morphologies that range from steady polarized forms that support high directionality to those more complex and variable when making frequent turns. Model simulations are essential for quantitative understanding of these features and their origins, however systematic comparisons with real data are underdeveloped. Here, by employing deep-learning-based feature extraction combined with phase-field modeling framework, we show that a low dimensional feature space for 2D migrating cell morphologies obtained from the shape stereotype of keratocytes, *Dictyostelium* and neutrophils can be fully mapped by an interlinked signaling network of cell-polarization and protrusion dynamics. Our analysis links the data-driven shape analysis to the underlying causalities by identifying key parameters critical for migratory morphologies both normal and aberrant under genetic and pharmacological perturbations. The results underscore the importance of deciphering self-organizing states and their interplay when characterizing morphological phenotypes.

## Author summary

Migratory cells that move by crawling do so by extending and retracting their plasma membrane. When and where these events take place determine the cell shape, and this is directly linked to the movement patterns. Understanding how the highly plastic and interconvertible morphologies appear from their underlying dynamics remains a challenge partly because their inherent complexity makes quantitatively comparison against the outputs of mathematical models difficult. To this end, we employed machine-learning based classification to extract features that characterize the basic migrating morphologies. The obtained features were then used to compare real cell data with outputs of a conceptual model that we introduced which describes coupling via feedback between local protrusive dynamics and polarity. The feature mapping showed that the model successfully recapitulates the shape dynamics that were not covered by previous related models and also hints at the critical parameters underlying state transitions. The ability of the present approach to compare model outputs with real cell data systematically and objectively is important as it allows outputs of future mathematical models to be quantitatively tested in an accessible and common reference frame.

## Introduction

Cell migration is a fundamental cellular process that underlies embryonic development, wound healing, immunological surveillance and cancer metastasis. In particular, fast migrating cells such as *Dictyostelium* and migrating immune cells are versatile in their patterns of movement that range from random exploratory movements with frequent turns to more persistent migration in a straight path. *Dictyostelium* cells exhibit both random migration [1] as a phagocyte, and more persistent migration when forming a fruiting body. Exploratory interstitial migration in leukocytes [2,3] underlies antigen search and immune surveillance [4], and some are also known to move in a straight line [5]. Frequency of cell turning and their angles is dictated by when and where branched networks of F-actin that drives formation of lateral protrusions called pseudopods occur. In *Dictyostelium*, pseudopods appear arm-like, and their formation and splitting randomizes cell orientation [6]. Selective maintenance of pseudopods thus provides directional bias in the shallow attractant gradients [7]. Similar F-actin enriched projections in immune cells vary in their appearance from those that are finger-like in DC cells to those more lamellar in neutrophils, however their role in directional choice appears to be conserved [8,9]. On the other hand, ability to move in a straight and persistent manner requires cell polarity which refers to a long-term state having a dominant leading edge enriched in branched F-actin meshwork and a trailing end with crosslinked actomyosin. In certain cells under geometrical confinement, buildup of hydrostatic pressure by contractility can rapidly switch the protrusion to a bleb which is devoid of F-actin [10]. Besides such cases, F-actin driven leading edge protrusion and rear contraction are concomitant in the polarized cells. While the particular shape that cells take depends on the extracellular conditions such as cell-substrate adhesion and diffusible attractants, shapes with broken-symmetry emerge in the absence of extracellular asymmetries, and thus their origins are cell-intrinsic by nature [6,7,11]. The fact that movement of fast migrating cells depend highly on self-deformation contrasts highly to those of mesenchymal cells such as fibroblasts, which move at an order of magnitude slower speed and are strongly dictated by the asymmetries introduced by the adhesive foci.

The common and recurring shapes observed under highly divergent culture conditions and across evolutionary distant species and taxa [12] suggest generality of the self-deforming dynamics in fast migrating cells. Transmigrating neutrophils, genetically or pharmacologically perturbed *Dictyostelium* [13,14] and certain cancer cells [15] take a canoe-like polarized morphology similar to fish-keratocytes and some protozoan amoebae. Conversely, polarized neutrophils under certain genetic and pharmacological perturbations are known to exhibit increased number of pseudopods [16,17]. These common and interconvertible morphologies suggest that they reflect basic self-organizing states of motile cells that can be quantified and compared with minimal reference to the details of the molecular underpinnings and the permissive extracellular conditions. Due to compounding levels of complexity, quantitative characterization of these canonical morphologies also requires one to leave aside fine-scale protrusions such as filopodia and endocytic cups and apply an appropriate coarse-grained description at the cellular-level. The aspect ratio of fish keratocytes was identified as the major variation in shape features by principle component analysis (PCA) [18]. Variation in more complex features requires other non-trivial measures of characterization. Fourier and related spectral analysis allows one to extract the periodicity in the protrusion-retraction cycle as well as in their spatial ordering [19]. Combined with PCA, Fourier description of *Dictyostelium* cell shape has shown that the morphologies observed at various steepness of a chemo-attractant gradient can be characterized in a two-dimensional feature space that represents differences in degree of elongation, splitting and polarization [20]. Zernike polynomials in combination with PCA has been used to classify invasive cancer morphologies in two-dimensional feature space [21]. Besides Fourier-based analysis, methods such as tracking of local curvature [22] and pseudopods at the cell edge [7,23] have been employed to characterize spatio-temporal dynamics of membrane protrusions. In RNAi screen of *Drosophila* culture cells, a large body of hand-picked morphology features has been employed to train a classifier by shallow neural networks [24] and Support Vector Machine [25]. These studies indicate that the states of physically realizable morphologies are confined to a relatively low-dimensional feature space [25]. The downside of data-driven approaches, however, are that the analysis often remains in a black-box making it difficult for one to understand data with reference to the underlying causalities.

A great challenge remains as to how one can quantitatively relate the characterized shapes to the underlying dynamics and vice versa [12,26,27]. For the most basic analysis, it is instructive to formulate a top-down model for isolated cells that is free of extracellular context [12], as behaviors under complex environments may later be deduced, given the repertoire of realizable dynamics, from spatial asymmetries and constraints in the key parameters. In the “graded radial extension model”, a polarized morphology similar to that observed in fish keratocytes and neutrophils is described without reference to the underlying mechanism by assuming that the plasma membrane extends radially and that its magnitude is spatially graded along the anterior-posterior axis [28]. Such a steady and graded distribution is thought to result from reaction-diffusion based symmetry breaking in the activity of the polarity signals GTPases Rac and RhoA at the plasma membrane that specifies the state of F-actin at each given place and time. Resource limitation that prevents one state from dominating the other is expected when the sum of the inactive and active form of the small GTPases is approximately fixed in time [29]. Bi-stable reaction-diffusion systems with the above constraint are known to support a protrusive membrane region (front) and contractile membrane region (rear) to co-exist in a spatially separate domains within a cell—a mathematical manifestation of a stable polarized cell shape [29]. On the other hand, pseudopods are transient structures regulated by locally amplified formation of branched F-actin networks. In *Dictyostelium* this is governed by transient activation in Ras/Rap and PI3K [30], and in case of neutrophils, by Cdc42 and PI3K [31,32]. Because the localized protrusive dynamics occur under uniform conditions, they are thought to arise by noise amplification by excitable regulatory network [23,30,32,33]. Cdc42 in neutrophils and Ras/Rap in *Dictyostelium* are also known to act positively to strengthen Rac and Rho and hence cell polarity [16,34–36].

Recent mathematical models addressed how interconvertible morphologies of random and persistent migrating cells can be described in a single framework. Modified models of excitability [14,37–40] introduce means to prolong propagation of wave-like activities which gives rise to elongated forms with directional persistence. Conversely, in bistable-based models [41], a recent modification to include large noise with memory [42] has demonstrated pseudopod-like dynamics. Despite added complexities, these models still fall within the realm of what excitability or bistability can describe. To which extent these two disparate schemes capture real cell morphologies have not been systematically and quantitatively addressed. In this work, we develop a framework that employs deep learning based classifier to obtain objective measures for shape comparison. By introducing a conceptual model that describes coupling between excitable and bistable regulation, we show that their interplay successfully maps experimentally observed morphologies across the full range of the feature space including those where the existing models fail. Furthermore, the model highlights key parameters that define morphologies under normal and aberrant conditions. The present approach provides a general and extendable framework to characterize varieties of other cell shapes in a data-driven manner which can then be interpreted and tested to further improve hypothesis-driven modeling. Provided that there are large sets of simulated timeseries and real data for feature extraction, the ability to help infer the migratory dynamics from snapshot images should also have practical single-cell applications for cell identification.

## Results

### A feature space related to cell polarity and pseudopod dynamics can be obtained from classification of stereotype morphologies by deep convolutional neural networks

**Fig 1:**
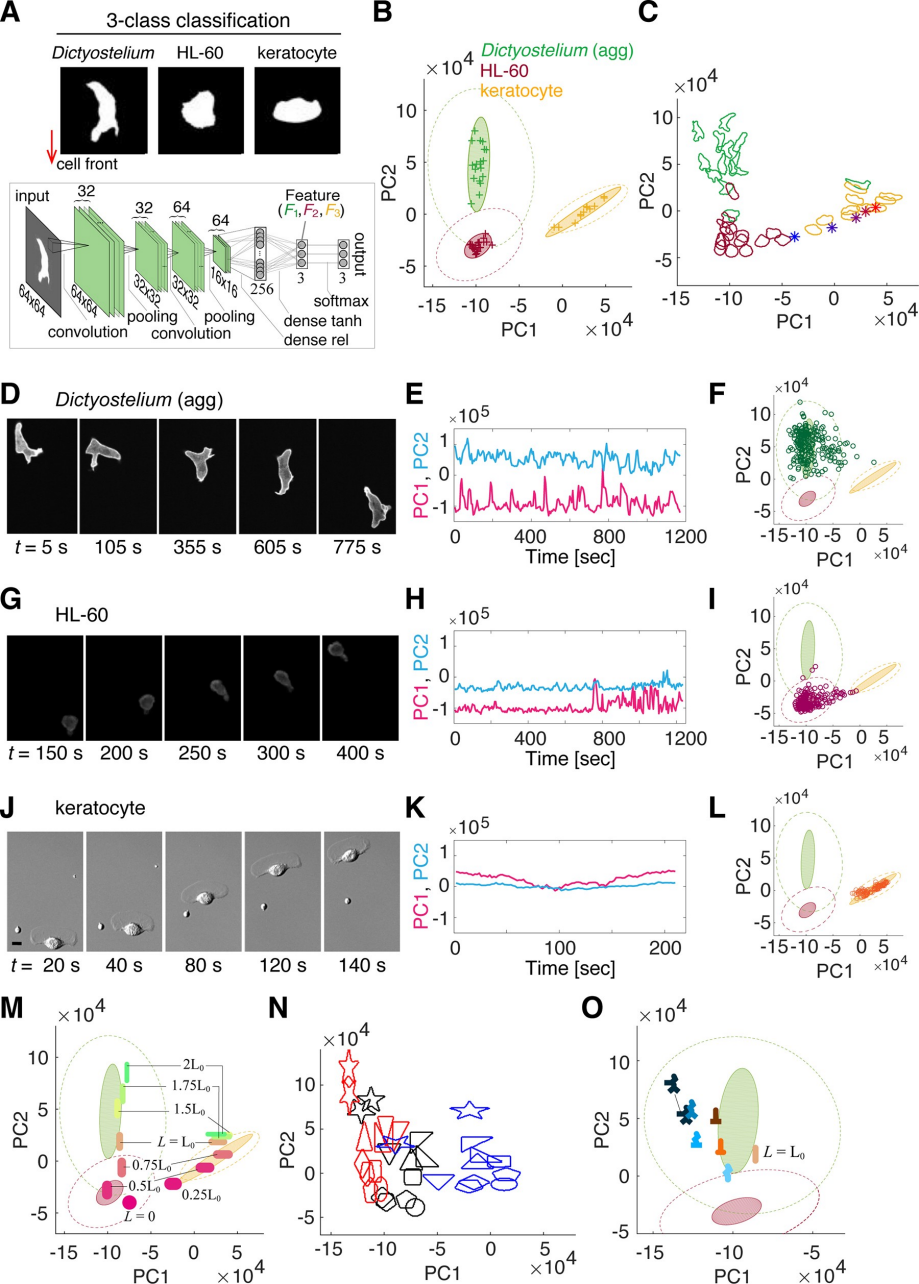
Stereotype migrating cell morphology can be classified in two-dimensional feature space. (A) Representative mask images (upper panels: *Dictyoselium* (agg; aggregation-stage), HL-60 and fish keratocyte) used for trainining a deep convolutional neural network (lower panel). Masks were normalized in area and aligned downwards in the migrating direction. The feature vector ***F*** = (*F*_1_, *F*_2_, *F*_3_) defined by the three nodes in the last layer was further reduced to PC1 and PC2 by PCA. (B) Mapping of trained data in PC1 and PC2 (dark green + (aggregation-stage *Dictyostelium*), dark red + (HL-60) and yellow + (keratocyte)). Each data point represents time-averaged scores from a time-series of a single cell. (C) Representative cell contours mapped to the feature space. Asterisks are the first principal shape variation of fish keratocyte (images taken from [18] (-2σ (blue) to +2σ from the mean (purple)). (D-L) Representative time-series and the corresponding feature scores for *Dictyostelium* (agg) (D-F), HL-60 (G-I) and fish keratocyte (J-L), respectively. (M-O) Mapping of skewed ellipsoidal shapes (the number indicates aspect ratio) (M), polygons (N) and a multi edge geometry (O) in the feature space. The circled regions in the background (B, F, I, M, O) are 95% confidence ellipses for the mean of all timeseries combined (dotted) and for the mean of individual cell (filled); green (*Dictyostelium*), dark red (HL-60), yellow (keratocyte).

For systematic extraction of cell morphology features from microscopy data, a convolutional neural network (Fig 1A, lower panel) was trained to classify snapshot mask images of: *Dictyostelium* (aggregation-stage; ‘agg’), neutrophil-like HL-60 and fish keratocytes (Fig 1A, upper panel; S1 Movie). The choice of the reference data was based on the fact that they are well-studied systems and that each represented a stereotype morphology that can be interpreted to represent different degree of pseudopod formation and cell polarity [6,12]. Under our experimental conditions, *Dictyostelium* cells showed an elongated form in the anterior to posterior direction with locally appearing pseudopods. Fish keratocyte took a canoe-like shape characterized by its long axis orthogonal to the moving direction. HL-60 exhibited an intermediate form between the two where, compared to *Dictyostelium*, transient protrusions appear less frequently and the overall shape was more horizontally elongated but to a lesser extent than the keratocyte. Image masks of these isolated single cells (Table A in S1 Text) were normalized in size and orientation (Fig 1A; see Methods). Hyper-parameters for deep-learning were chosen for relative high-accuracy for various network structures. The extracted features were well trained as judged by the high validation accuracy; 97.9% and 89.7%, for the training and the validation data respectively (S1A and S1B Fig; the mean of the last 10 epochs; see Table B in S1 Text for accuracy per dataset). The classification accuracy of the validation data of *Dictyostelium*, HL-60, and keratocyte is 94.6%, 96.0%, and 87.8%, respectively (Table B in S1 Text). The three nodes ***F*** = (*F*_1_, *F*_2_, *F*_3_) that constituted the second to last layer of the network showed good representation of the three data classes: *Dictyoselium* (S1C Fig; high *F*_1_), HL-60 (S1D Fig; high *F*_2_) and keratocyte (S1E Fig; high *F*_3_), which were further reduced to two by principal component analysis (PCA). The latency values of PC1, PC2 and PC3 were approximately 66.3%, 33.5% and 0.3%, respectively. The contribution of *F*_1_, *F*_2_ and *F*_3_ to PC1 = (-0.42,-0.35, 0.84)•***F*** and PC2 = (0.76,-0.64, 0.11)•***F*** indicate that PC1 highlights keratocyte-like shapes while penalizing features common to *Dictyostelium* and HL-60, and PC2 favors *Dictyostelium*-like features not found in HL-60. Fig 1B shows good separation of the three datasets in the PCA space (~ 92.8% accuracy) compared to classification based on hand-crafted features (S2D Fig, Tables Q and R in S1 Text). The keratocyte and *Dictyostelium* (agg) dataset were found in the high PC1 and high PC2 regions, respectively. The HL-60 dataset were mapped to a low PC1 low PC2 region.

The feature metrics acquired above, by the very fact that they constitute a good classifier, should be useful to quantify similarity of cell morphology of one’s interest in reference to the trained data. The overall relationship between representative cell contours and the morphology feature is shown in Fig 1C. At first glance, higher PC1 appears to indicate more pronounced elongation in the lateral direction while higher PC2 indicates marked longitudinal elongation and protruding edges. These variations in the feature space not only reflected the average morphology differences between the three training classes but also shape changes in time. Fig 1D–1l show representative single-cell timeseries from the validation dataset; i.e. a reserved dataset not used for training. *Dictyostelium* data showed large fluctuations in both the PC1 and PC2 direction (Fig 1D, 1E, and 1F), whereas the HL60 (Fig 1G, 1H, and 1I) and keratocyte data (Fig 1J, 1K, and 1L) exhibited more marked changes in the PC1 direction (see also S1C–S1E Fig for changes in ***F***). To clarify the relationship between the PC1-PC2 and the extent of elongation, we re-visited the principle component of the fish keratocyte shape variation reported earlier (‘shape mode 1’ in [18]). Low aspect-ratio shapes (-2*σ* in ‘shape mode 1’ [18]) were located near the HL-60 dataset, and high aspect-ratio shapes (2*σ* in ‘shape mode 1’ [18]) were located near the keratocyte dataset. Consequently, the shape along the ‘shape mode 1’ of fish keratocyte [18] constitutes a well-confined manifold in the PC1-PC2 space (Fig 1C; asterisks) to which our fish keratocyte data were also mapped. Furthermore, ellipsoidal shapes with various aspect ratios indicate that an increase in the lateral elongation maps them on the same manifold as the keratocyte dataset (Fig 1M, PC1 > -1; magenta to pink ellipsoids) while an increase in the degree of head-to-tail elongation maps the ellipsoids to another manifold along the PC2 axis (Fig 1M, green ellipsoids). Rotating these ellipsoids to intermediate angles map these shapes to intermediate PC1 values (S1F and S1G Fig).

While the analysis of the stretched ellipsoids clearly demonstrated how the cell orientation and the degree of elongation are mapped to the feature space, the *Dictyostelium* (agg) dataset was markedly offset from these ellipsoids towards negative PC1 values. To further clarify the nature of this deviation, well-defined polygons were subjected to the same feature analysis (Materials and Methods). All 33 geometrical objects tested were mapped within the region of the PC1-PC2 space spanned by the microscopy data (Fig 1N). Regular polygons and circles were mapped to a low PC1—PC2 region and their vertically stretched counterparts were mapped to higher PC2 region (Fig 1N, red). Polygons and ellipsoids that were stretched in the lateral direction marked high PC1 value (Fig 1N, blue; S1H and S1I Figs). Of particular note were the star objects which scored highest in the PC2 value and deviated markedly in the feature space from the ellipsoids and other objects when stretched (S1I Fig). Stars have higher PC2 compared to squares and triangles indicating that PC2 reflects pointed edges. Comparisons between upright and vertically flipped stars and triangles indicated that degree of pointedness towards the cell front also affects PC2 but in a complex way (Fig 1N; see also S1J Fig). An analysis of more asymmetrical geometries with varying number of edges showed that they map to a domain in high PC2 with high variations towards negative PC1 values as in the *Dictyostelium* (agg) dataset; i.e. away from ellipsoidal shapes (Fig 1O). Rotating the multi-edge forms with the small PC1 values (Fig 1O; 0 to 30 degrees) bring about decrease in PC1 (S1K Fig). Further rotation increases PC1 (S1K Fig; 60 to 90 degrees) due to the shape now appearing more horizontally elongated overall. Generality of the results was confirmed in independent real-cell data by analyzing fully differentiated prespore cells of *Dictyostelium* which is elongated longitudinally and lacks pseudopods thus mapping identically to the elliposoids (S3A and S3B Fig). Likewise, effector T cells with their signature branching protrusions were broadly distributed along PC2 (S3C Fig, Th1) compared to markedly less polarized regulatory T cells (S3D Fig, Treg). These analysis indicate that our classifier was able to yield data-driven representation of complex signatures with respect to the cell orientation for both local pseudopodal protrusions and more global cell elongation.

### The ‘ideal cell’ model recapitulates a generalized morphological landscape constrained by the choice of the protrusion speed and the balance between the local protrusion and global polarity

**Fig 2:**
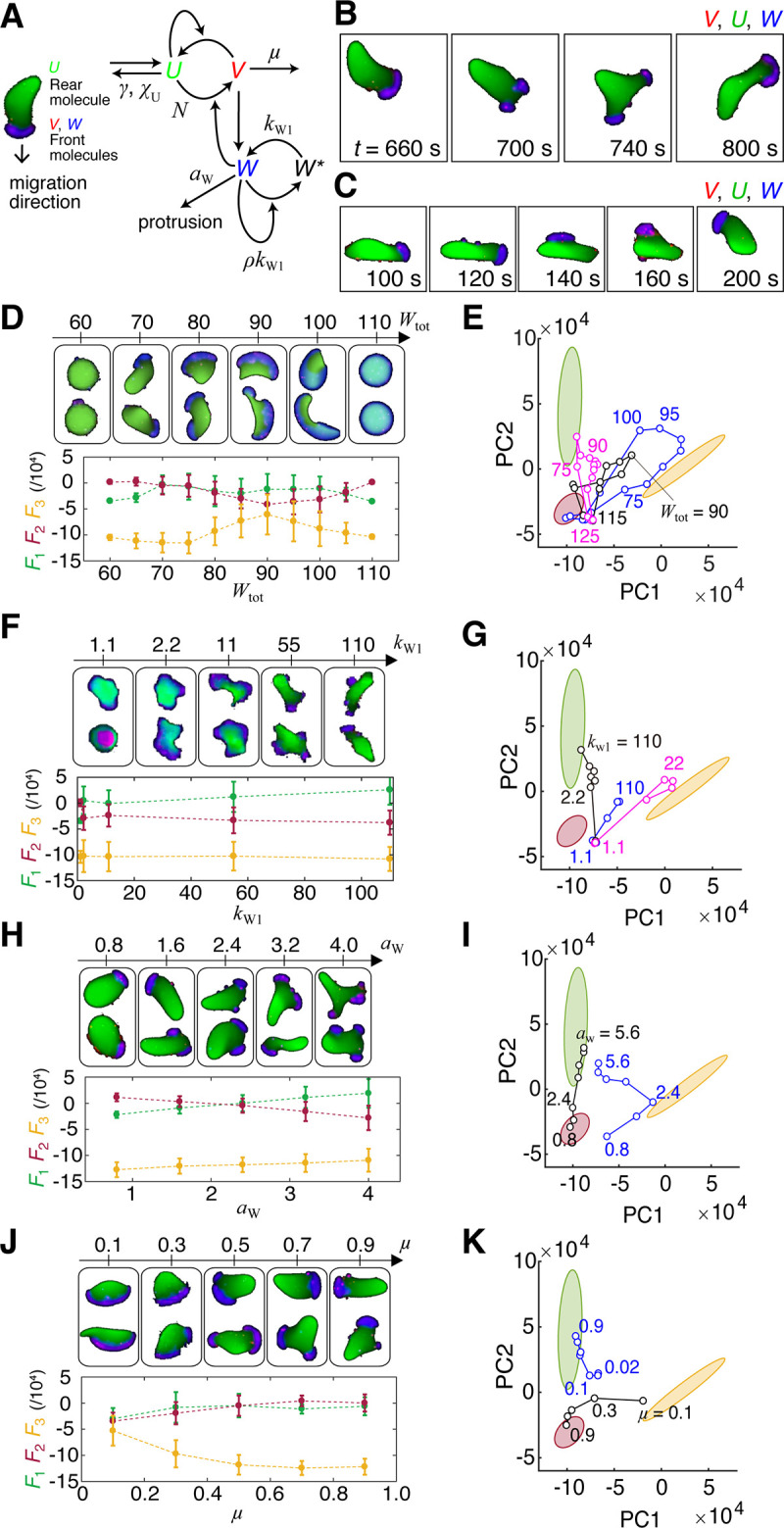
Degree of leading edge expansion and the balance between pseudopod formation and polarity persistence can account for the main morphology feature. (A) Schematics of the dynamical model. Self-amplifying and excitatory synthesis from *U* to *V* [70] induces protrusion factor *W* which can be prolonged by the bistable dynamics (Eq 2). The reaction takes place in a region *ϕ* = 1 governed by interface physics according to the phase-field equation (Eq 1). The membrane expands outward into an unoccupied region *ϕ* = 0 in the direction perpendicular to the border at a rate proportional to local *W*. Main parameters (*χ*_U_, *γ*, *μ*, *k*_W1_, *ρ*, *a*_W_) are denoted along the associated reaction steps. (B, C) Representative model behavior (overlay plots for *U*, *V*, *W*); front splitting (B) and pseudopod formation (C). (D-K) Parameter dependence of cell morphology (D, F, H, J upper panels), the average feature ***F*** from 2–4 independent simulation runs (D, F, H, J lower panels) and time averaged PC1-PC2 values (E, G, I, K); (D) *W*_tot_ (*χ*_U_ = 50, *k*_W1_ = 90, *μ* = 0.5, *ρ* = 5.5556, *γ* = 0.1, *a*_W_ = 2.4, *D*_W_ = 3). (E) Black same as (D), blue (*χ*_U_ = 50, *k*_W1_ = 100, *μ* = 0.1, *ρ* = 5, *γ* = 0.1, *a*_W_ = 2.4, *D*_W_ = 3) and magenta (*χ*_U_ = 50, *k*_W1_ = 110, *μ* = 0.5, *ρ* = 4.5455, *γ* = 0.1, *a*_W_ = 4, *D*_W_ = 3). (F) *k*_w1_ (*χ*_U_ = 50, *μ* = 0.5, *ρ* = 4.5455, *γ* = 0.1, *a*_W_ = 5.6, *D*_W_ = 3, *W*_tot_ = 80). (G) Black same as (F), blue (*χ*_U_ = 50, *μ* = 0.5, *ρ* = 4.5455, *γ* = 0.1, *a*_W_ = 2.4, *D*_W_ = 3, *W*_tot_ = 90), and magenta (*χ*_U_ = 50, *μ* = 0.1, *ρ* = 4.5455, *γ* = 0.1, *a*_W_ = 2.4, *D*_W_ = 3, *W*_tot_ = 80). (H) *a*_W_ (*χ*_U_ = 30, *k*_W1_ = 110, *μ* = 0.5, *ρ* = 4.5455, *γ* = 0.1, *D*_W_ = 3, *W*_tot_ = 80). (I) Black same as (H), blue (*χ*_U_ = 50, *k*_W1_ = 100, *μ* = 0.1, *ρ* = 5, *γ* = 0.1, *D*_W_ = 3, *W*_tot_ = 80). (J) *μ* (*χ*_U_ = 50, *k*_W1_ = 100, *ρ* = 5, *γ* = 0.1, *a*_W_ = 2.4, *D*_W_ = 3, *W*_tot_ = 80). (K) Black same as (J), the blue line is (*χ*_U_ = 50, *k*_W1_ = 110, *ρ* = 4.5455, *γ* = 0.1, *a*_W_ = 4.8, *D*_W_ = 3, *W*_tot_ = 80).

Let us now introduce an ideal cell model (see Equations: Eqs 1 and 2) that serves as a canonical shape generator of migrating cells that is strictly constrained by the transient protrusion dynamics and polarization (S1 Text). To describe the interfacial membrane mechanics, we employed the phase-field method with the addition of an active force *F*_prot_ = *a*_w_*W* (Eq 1; Table C in S1 Text). The model also consists of spatio-temporal dynamics of variable *W* and variables *U*, *V* that define global cell polarity and local protrusions, respectively (Fig 2A; Eq 2). These variables are abstract representation of how the respective signaling molecules—such as Rac, Rho for *W* and Ras, Cdc42, PI3K for *V*—are regulated in space and time (Materials and Methods). *W* is bistable and takes either a low or a high state which signifies the retracting rear and the expanding front, respectively. The low state indicates that *W* has converted to the other form W* while the integrated sum of *W* and W* is fixed to *W*_tot_. Owing to this constraint, the dynamics of *W* exhibits wave-pinning and therefore supports a polarized shape with *W* being high at one end and low at the other end (Fig 2B, *t* = 660s). In addition, there is an excitable network that describes conversion of *U* to *V* which is invoked by small signal fluctuations. The important assumption in the model is that these two core networks are coupled. On top of the core bistability in *W*, *V* promotes conversion of *W* from the low to the high state, and *W* catalyzes amplification of *V* (Fig 2A). Because *V* amplifies noisy fluctuations and generates local protrusions through *W*, a leading edge defined by a region with high *W* is most likely to be perturbed and often split into two (Fig 2B; *t* = 700). This is however transient, as *W* by itself works to maintain global unipolarity hence only one protrusion survives (Fig 2B; *t* = 800s). In some cases, a new protrusion can also form away from the anterior and more towards the lateral side and still develop into a new dominant front (Fig 2C). These features required full 3-variable equations (Eq 2; Table D in S1 Text) and were of particular importance in our comparative analysis. Insofar as our parameter search (Tables E and F in S1 Text), neither the dynamics of *U* and *V* (S4A–S4D Fig) nor that of *W* alone (S4E–S4I Fig) supported these bifurcating protrusions (S1 Text). Although our model encompasses the 1- and 2-variable limits which describe well morphologies outside of the training dataset such as oscillatory non-migratory shapes (S4B and S4C Fig) and non-bifurcating polarized cells (S4F and S4G Fig), we shall exclude these parameter regimes from the following analysis. Related oscillatory and fan-like morphologies have been addressed earlier [37,43,44].

In total, 228 parameter conditions (Table G in S1 Text) were selected by heuristic sampling where we performed grid search in the parameter subspace around hand-picked reference points (S5 Fig; Material & Methods). The advantage of the ‘ideal cell’ model over the existing models focused on specific cells and conditions is that, it can describe a generalized morphological landscape incurred by the choice of the protrusion speed and the balance between the local protrusion and global polarity. As we shall describe later, this versatility allows us to resolve and characterize cells in different developmental stages and under perturbations. First, of particular interest was whether the cell was elongated in the lateral or anterior-posterior direction. We found *W*_tot_ has a large influence in the direction and the degree of overall cell elongation (Fig 2D; see also S2 Movie). For small *W*_tot_, the system behaves as a passive interface that relaxes to the state of minimum surface energy of fixed area, thus the simulated morphology was near circular (Fig 2D, *W*_tot_ = 50–70). As *W*_tot_ increased, a small region with *W* appeared, and cells became elongated (Fig 2D, *W*_tot_ = 80, 90). A further increase in *W*_tot_, expanded the high *W* region (Fig 2D, *W*_tot_ = 100) until it encompassed the entire perimeter (Fig 2D, *W*_tot_ = 110), where the simulated morphology was again near circular because *W* occupied an entire cell. Accordingly, moderately high *W*_tot_ (*W*_tot_ = 80) supported relatively high *F*_1_ and high *F*_2_ on average (indicating shapes resembling *Dictyostelium* and HL-60) (Fig 2D; bottom panel). In the PC1-PC2 space, small *W*_tot_ yielded low PC1 and low PC2, whereas at a moderately high *W*_tot_, PC2 took high values (Fig 2E). At high *W*_tot_, the average PC1 and PC2 decreased. The overall dependency on *W*_tot_ were conserved when other parameters were varied (Fig 2E; magenta and blue). The other important parameters that affected cell polarity was *ρ*; the strength of autocatalysis in the interconversion reaction between W and W*. High *ρ* means that the non-zero roots of the cubic equation −*ρW*^3^+ρW*W^2^−*W* = 0 is large thus supporting a larger domain with high *W*. Therefore, at low *ρ*, the leading edge is small and cells become elongated in the moving direction (S5A Fig; *ρ* = 4.55). As *ρ* is increased, leading edge became broader and *F*_3_ increased (S6B Fig; *ρ* = 5.56).

Occurrence of local protrusions depended largely on *k*_W1_ and *a*_W_. *k*_W1_ specifies the depth of the bistable well. Because *k*_W1_ by definition defines how strong protrusive activity is dominated by bistability of W rather than through V, it can be viewed as representation of relative activity in Arp2/3 and crosslinker Myosin II. For small *k*_W1_, the front-rear asymmetry was weak, and the overall cell shape was near circular (Fig 2F; *k*_W1_ = 1.1). Intermediate *k*_W1_ gave rise to mixed dynamics where local protrusions induced asymmetrical deformation however without persistent front-to-back polarity (Fig 2F; *k*_W1_ = 2.2–55). Large *k*_W1_ elevates *W* which makes it less affected by the dynamics of *U* and *V*, and thus supports elongated shape with more marked polar asymmetry in *W* (Fig 2F, *k*_W1_ = 110; S3 Movie). Accordingly, we obtained high *F*_1_ (i.e. high resemblance to *Dictyostelium* (agg)) (Fig 2F, lower panel), and hence high PC2 (Fig 2G). Deletion of myosin light chain reduces pseudopodia and strengthens polarity in *Dictyostelium* [45]. Conversely, increased myosin light chain kinase expression is known to reduce lamellipodia size and induce multiple protrusions in keratocyte independently of Rho kinase and membrane tension [46]. The appearance of local protrusions also depended strongly on the protrusion force *a*_W_ (Fig 2H; S4 Movie). For low *a*_W_ (Fig 2H; *a*_W_ = 0.8), only small deformation was observed and the overall cell shape was near circular. At an intermediate value of *a*_W_ (Fig 2H; *a*_W_ = 1.6–2.4), cells were more longitudinally elongated and the cell displacement was more directional. At high *a*_W_ (Fig 2H; *a*_W_ = 3.2–4.0), multiple pseudopods appeared, and the cell orientation changed frequently (S4 Movie). There was an increase and a decrease in *F*_1_ and *F*_2_ respectively (i.e. high resemblance to *Dictyostelium* (agg)) (Fig 2H, lower panel). The PC2 score increased accordingly (Fig 2I).

Parameters that affected the pseudopod dynamics were *μ* and *γ* which define the downregulation rate of *V* and *U*, respectively. Excitable dynamics in neutrophils and *Dictyostelium* are associated with PIP3, and thus *μ* can be viewed as activity of phosphatases and kinases other than those directly involved in conversion between U and V. Low *μ* (Fig 2J; *μ* = 0.1; S5 Movie) elevates *V*, hence the concomitant increase in *W* supported a laterally elongated shape. *Dictyostelium* cells are known to take similarly elongated form when perturbed with 5-phosphatase that effectively reduces plasma membrane PTEN and increases PIP3 [14]. The polarized shape was highly persistent as high *V* renders the patterning less prone to noise perturbation. On the other hand, at intermediate to high value of *μ*, cells became more elongated longitudinally and the polarity was less persistent (Fig 2J; *μ* = 0.5–0.9). Here, the high *W* domain was easily disrupted; fronts frequently split, and the cell orientation was altered (e.g., a Y-shaped front in Fig 2J at *μ* = 0.7 and 0.9). Accordingly, *F*_3_ (Fig 2J; bottom panel) and the PC1 score decreased at high *μ* (Fig 2K). Similarly, at low *γ*, pseudopods split frequently and new pseudopods were rare (S6C and S6D Fig; *γ* = 0.1) and the opposite was true for high *γ* (S6C and S6D Fig; *γ* = 0.5 or 0.7). Additionally, for splitting to occur, it was important that diffusion of *W* does not average out the local perturbations. Broad leading edge split at low *D*_W_, (S6E Fig; *D*_W_ = 0.6, 1.8 and 2.4), but was sustained at high *D*_W_ (S6E Fig; *D*_W_ = 3.6). These details only made subtle changes in our morphology feature (S6E and S6F Fig). The boundary flux *χ*_U_, was also important to restrict the *U*-*V* reaction at the edge (S4B and S4C Fig). Splitting of the front occurred more frequently at high *χ*_U_ (S6G Fig)_._ Due to the temporal nature, the feature vector on average remained almost unchanged (S6H Fig).

### Morphology-based mapping of model parameters can help infer candidate dynamics

**Fig 3:**
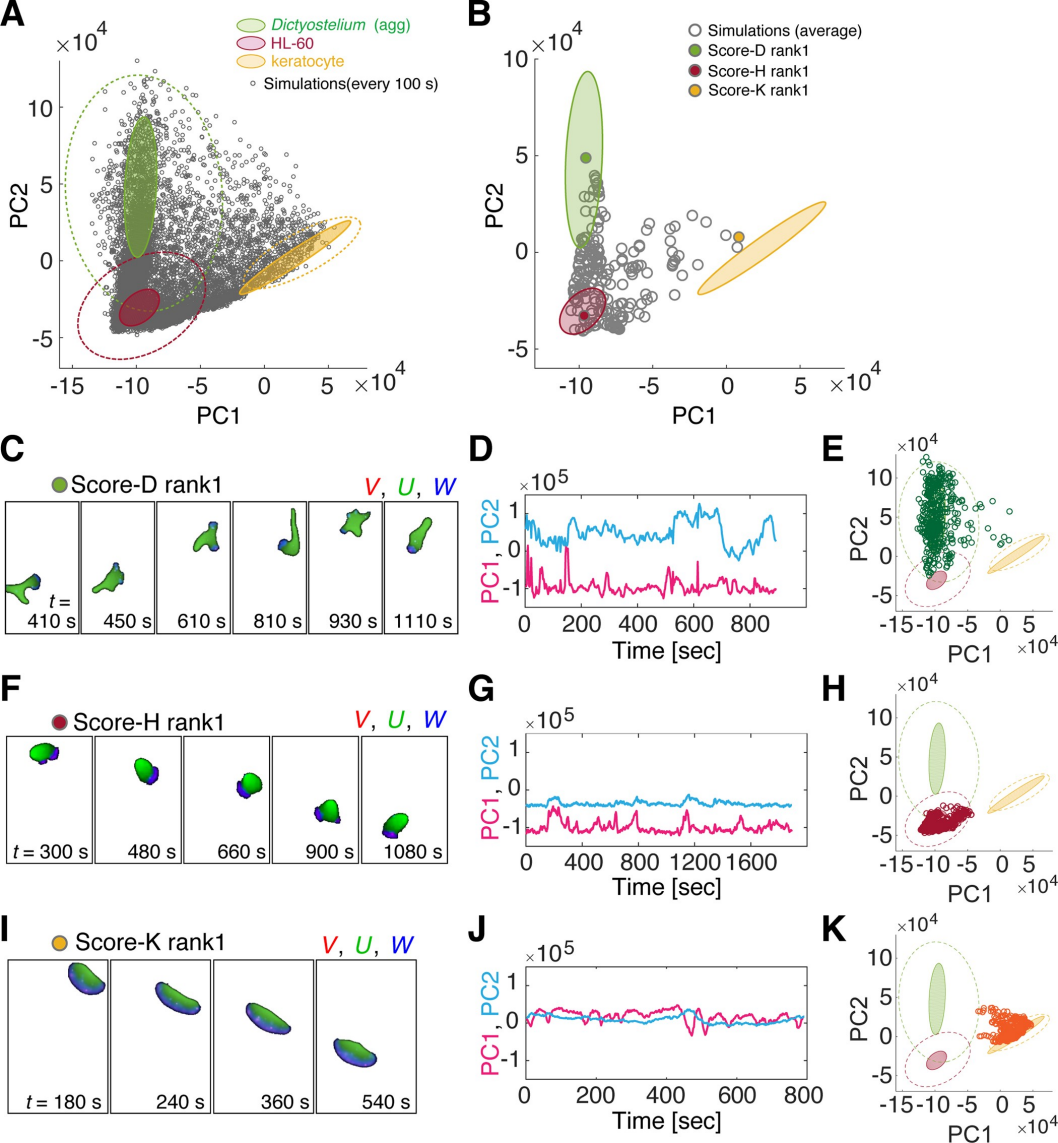
Mapping of morphology features between simulated and real cell data confirms critical parameters that define the morphology type. (A, B) Mapping of simulated cell morphology from the 228 parameter sets (Grey open circle: subsampled time series (A) and time averages for 2 to 4 independent runs (B). Filled circle: simulations with the highest similarity to aggregation-stage *Dictyoselium*-rank1 (B, green), HL-60 (B, red) and fish keratocyte (B, orange). (B) The 95% confidence ellipses of the trained datasets (aggregation-stage *Dictyostelium* dark green (dark green), HL-60 (dark red) and fish keratocyte (yellow) in Fig 1B (A, B) are shown for reference. (C-K) Snapshots and the feature scores of the top-ranked simulations; aggregation-stage *Dictyostelium* (C-E), HL-60 (F-H) and fish keratocyte (I-K). See Table N in S1 Text for parameter values.

The distribution of the simulation data in the PC1-PC2 space spanned a large region occupied by the training dataset (Fig 3A, black circles) further vindicated the ability of the ideal cell model to describe the characteristic morphologies. Proximity of the time-averaged simulated morphologies (Fig 3B) to the average of the three reference dataset was analyzed by computing the Euclidean distance in the feature space ***F*** = (*F*_1_, *F*_2_, *F*_3_). According to the reference data, the distance was designated as Score-D (*Dictyostelium)*, Score-H (HL-60), Score-K (keratocytes), and ranked in the ascending order; i.e. a low score means high similarity (Table H in S1 Text). The time averaged morphology feature in the PC1-PC2 space and the time-series of the top ranking simulations are shown in Fig 3B (filled circles) and Fig 3C–3K, respectively. Simulations with high Score-D on average exhibited morphology that closely resembled the aggregation-stage *Dictyostelium* with their elongated form in the anterior-posterior direction accompanied by a few pseudopods that frequently reoriented cell directionality (Fig 3C; S6 Movie). Similarly, simulations that ranked high for Score-H (Fig 3F; S7 Movie) exhibited fan-like cell shape that moved directionally with some occasional turning as observed in HL-60. These high ranking parameter sets were found near the median of the reference dataset in PC1-PC2 space (Fig 3E, 3H, and 3K). When high-ranking simulations were retested for classification per snapshot, close to or higher than 90% were correctly classified as *Dictyostelium* (Table I in S1 Text) or HL-60-like (Table J in S1 Text). As for high Score-K simulations, the simulated shapes were canoe-like with high directional persistence (Fig 3I; S8 Movie). On average, they deviated transiently in the PC1 direction thus making classification per snapshot less clear (< 66% accuracy). The deviation was due to occasional shape perturbation by random noise (S7A and S7B Fig) which can be removed by reducing noise in the simulation (S7C and S7D Fig). The overall mapping of real cell data and model simulations were conserved when intermediate layer of the classifier was used to obtain the feature space (S1 Text; S8 Fig; Table M in S1 Text).

**Fig 4:**
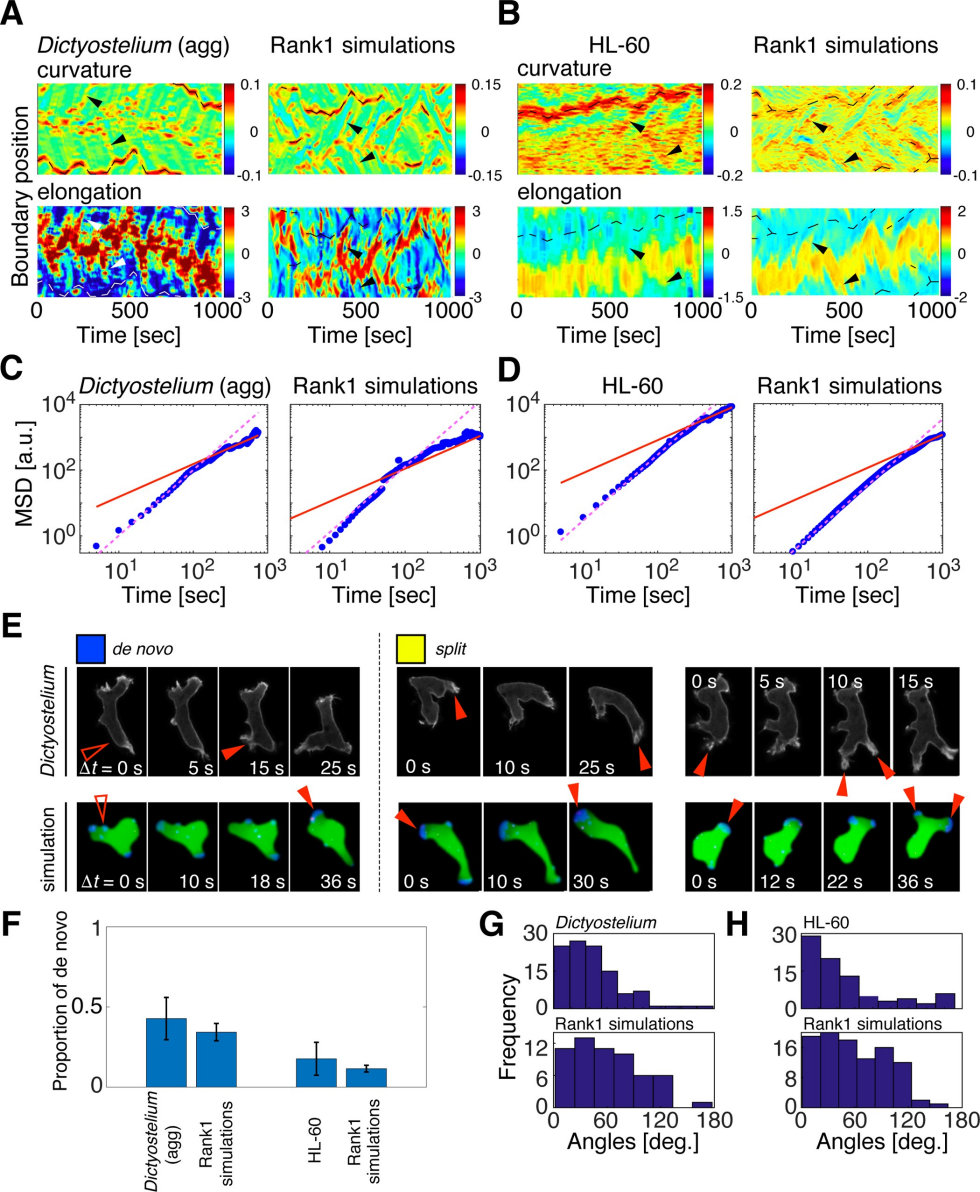
Consistent exploratory dynamics underlie high similarity in the simulated morphologies. (A, B) Local boundary curvature (top panels) and the protrusion speed (bottom panels) [47] from the real cell data (left panels) and the top ranked simulations (right panels) for *Dictyostelium* (agg) (A) and HL-60 (B). (C, D) Mean square displacement of the centroid of *Dictyostelium* (agg) (C) and HL-60 (D); real cell data (left panels) and top ranked simulations (right panels). The length scale was normalized by the mean cell length in the moving direction. (E) Representative snapshots of pseudopod formation; de novo formation (left panel) and splitting of an existing pseudopod (one-way-split (middle panel) and Y-shape (right panel)). (F) Fractional occurrence of pseudopods by de novo formation; *Dictyostelium* (agg) (left) and HL-60 (right). (G-H) Histogram of pseudopod angles obtained from the time-series of real cell data (A-D left panels) and the top ranked simulations for *Dictyostelium* (agg) (G) and HL-60 (H).

Although similarity was evaluated based on still images, dynamics of high ranking simulations were by and large consistent with those of real cells. This was well illustrated in the kymographs of boundary curvatures (Fig 4A and 4B upper panels); the anterior and posterior regions can be identified by high curvature regions with either positive or negative velocity respectively (Fig 4A and 4B lower panels). Both *Dictyostelium* and HL60 (Fig 4A and 4B left panels) showed anterior projections (Fig 4A and 4B bottom panel red regions) that bifurcated from time to time and traveled towards the high curvature region at the rear [47]. The wave-like appearance was somewhat more prominent in *Dictyostelium*. In both cell types, the posterior end was characterized by a high curvature region that persisted over time. These dynamical features were well recapitulated in the simulations (Fig 4A and 4B right panels). Moreover, there was good agreement between the simulations and the real data in the cell trajectories. The mean square displacement (MSD) of the centroid showed a characteristic time-scale dependency where it was proportional to the square of the elapsed time (*ΔT*^2^) for *ΔT* < *τ*_0_ (Fig 4C, magenta line) and to *ΔT* for *ΔT* > *τ*_0_ (time domain, Fig 4C red line). In other words, cells moved ballistically i.e. at a constant velocity for *ΔT* < *τ*_0_ and more like a Brownian particle for *ΔT* > *τ*_0_. *τ*_0_ can be interpreted as the persistence time for directional migration, and square root of the MSD at the inflection point *X*_0_ characterizes the persistence length. Throughout this paper, we chose the time-scale factor *τ*′ = 10 based on approximate matching in the crossover point of the two regression lines between the top ranking simulations and the real-cell data (Fig 4C and 4D; red lines). For the top Score-D simulations, we obtained *τ*_0_ = 87 sec, *X*_0_ = 9.8 cell length, compared to *τ*_0_ = 151 sec and *X*_0_ = 15.2 cell-length in the real data which are in good agreement with values reported earlier [48]. Trajectories of top ranking simulations for Score-H were more persistent (*τ*_0_ = 257 sec, *X*_0_ = 22.1 cell length) as was the case for the real HL60 data (*τ*_0_ = 278 sec, *X*_0_ = 47.7 cell length).

The other important feature of random migration is the relation between pseudopod dynamics and the cell orientation [7]. New pseudopods frequently appeared in vacant regions (Fig 4E left), or on top of a pre-existing pseudopod thereby giving the cell the appearance of Y-shape (‘Y-split’ in [7]). In other cases, pseudopods continued to extend while turning (‘one-way-split’ in [7]). The relative occurrence of de novo formation of pseudopods was approximately 47% for *Dictyostelium* (agg) data and 34% for the high ranking simulations (Fig 4F). For HL60, de novo formation in real cell data was 12% and in the high ranking simulations 21% (Fig 4F). The extension angles relative to the direction of centroid displacement was about 20–40° for both *Dictyostelium* and HL60 data and their top-ranking simulation counterparts (Fig 4G and 4H). Although there was some overrepresentation of extension angles around 90° in the simulation, the angles above 120° were rare in both real data and simulations. All in all, these results demonstrate that the model, albeit its simplification, is able to recapitulate semi-quantitatively both the persistent random walk behavior and the underlying morphology dynamics in *Dictyostelium* and HL-60 cells.

### Mapping of morphological diversification predicts key parameters for state transition

**Fig 5:**
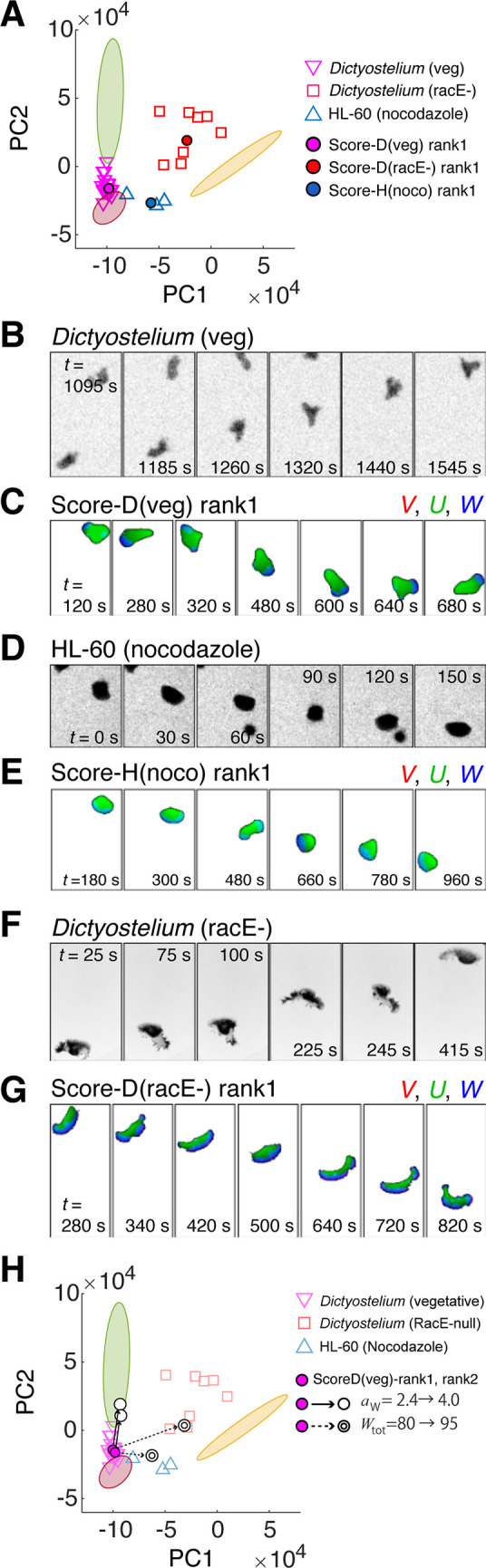
Mapping of non-trained morphologies with perturbing cell polarity predicts a continuous landscape along critical parameters. (A) Morphology features of real cell data from *Dicyostelium* (veg; vegetative stage) (pink inverted triangle), *Dicyostelium* racE- (red square) and nocodazole-treated HL-60 (blue upright triangle) and those from simulations exhibiting highest similarity (filled circles). Colored regions in the background represent the reference microscopy data in Fig 1B (aggregation-stage *Dictyostelium* dark green (dark green), HL-60 (dark red) and fish keratocyte (yellow)). (B-G) A time-series sample of experimental data closest to the average morphology and the corresponding top-ranked simulations; vegetative-stage *Dictyostelium* (B, C), nocodazole-treated HL-60 (D, E) *Dicyostelium* racE- (F, G). (H) The top-ranked simulations (rank1 and rank2; magenta circles) for *Dicyostelium* (veg) shifted toward *Dictyostelium* (agg) by shifting *a*_W_ from 2.4 to 4 (solid line), and toward the orange region by shifting *W*_tot_ from 80 to 95 (dotted line). See Table N in S1 Text for parameter values.

Although the datasets analyzed above showed little overlap with one another in the feature space, it should be noted that these coordinates are by no means singular representation of specific cell-types and species from which the data were obtained. As we saw above, there was a large cell-cell variability in the fish keratocyte data that constituted a distinct manifold in the feature space (Fig 1B; yellow). Likewise, cell-cell variability was evident in the aggregation-stage *Dictyostelium* cells along the PC2 axis (Fig 1B; green). To see how changes in cell-intrinsic properties alter their positions in the feature space, data from new experimental conditions expected to alter cell polarity were studied (Fig 5A). Undifferentiated (vegetative) *Dictyostelium* cells took less elongated shape than the aggregation-stage *Dictyostelium* cells under the same substrate and buffer condition. Their aspect ratio on average was smaller than aggregation-stage *Dictyostelium* but larger than that of HL-60 (Fig 5B). Accordingly, in the PC1-PC2 space, the vegetative *Dictyostelium* was mapped between aggregation-stage *Dictyostelium* and HL-60 (Fig 5A; magenta reverse triangles). Model simulations that ranked similar to the vegetative *Dictyostelium* data (Fig 5C; S9 Movie) had small *a*_w_ in common (Table L in S1 Text). Similarly, we analyzed HL-60 cells treated with microtubule destabilizer nocodazole which is known to strengthen neutrophil cell polarity [49,50]. Nocodazole-treated HL-60 cells showed morphology similar to keratocyte with a somewhat smaller aspect ratio (Fig 5D) and were mapped between the non-treated HL-60 and the keratocyte datasets (Fig 5A; blue triangle). The nocodazole-treated HL-60 cells exhibited shape fluctuations making them wobble which was also observed in the simulations (S10 Movie). These features were well represented in the respective simulations that ranked high for shape similarity (Fig 5E). In addition to high *W*_tot_, high ranking simulations had relatively low *a*_w_ or *k*_w1_ (Table L in S1 Text). This can be interpreted from the fact that low *a*_w_ prevents a cell from breaking apart at high *W*_tot_, while low *k*_w1_ makes the polarized front less pronounced and more sensitive to noise perturbation.

Next, a null strain of *racE* (*Dicytostelium* RhoA homologue) was chosen for the analysis because of its aberrant cell shape that resembled fish keratocytes [51]. Under our experimental condition, relatively undifferentiated *racE*- cells exhibited canoe-like shapes that were similar to the fish keratocyte but more dynamic (Fig 5F). Small fragmented pieces were observed to occasionally split from high curvature regions (Fig 5F). These data marked relatively high PC1 and PC2 scores and were mapped between the keratocyte and the aggregation-stage *Dictyostelium* data (Fig 5A; black square). The top ranking simulations for the *racE*- data (Table L in S1 Text; high Score-D (*racE*-)) exhibited remarkably similar morphology dynamics characterized by high lateral deformation and occasional fragmentation (Fig 5G; S11 Movie). As expected, there was a large overlap with high Score-K data, leaving only the top 3 Score-K data (Table H in S1 Text bottom rows; ||*F*||_2_ < 3 x 10^4^) that uniquely mapped to the keratocyte data. Compared to either the high Score-D (veg) or the high Score-D (agg) data, the high Score-D (*racE*-) data had large *W*_tot_ (Table L in S1 Text) consistent with the laterally extended cell shape. All in all, the above analysis suggests key parameters *a*_w_ and *W*_tot_ that are pivotal for the state transition between the characteristic morphologies. This was further verified by studying unexamined regions in the high-dimensional parameter space near the top ranking simulations for vegetative-stage *Dictyostelium*. Increasing the value of *a*_w_ brought the morphology score closer to that of the aggregation-stage cell (Fig 5H). In contrast, increasing *W*_tot_ increased PC1 and brought the shape state closer to the nocodazole-treated HL-60 and *racE*- cells (Fig 5H).

## Discussion

Deep learning-based extraction of shape features is a promising avenue for cell classification and identification [52]. In this study, we presented a hybrid cell morphology analysis that combined deep-learning-based feature extraction and dynamical model simulations. Convolutional neural network was trained to classify stereotype migrating cell morphology represented by *Dictyostelium*, HL-60 and fish keratocytes. The feature vector of the trained classifier showed that the three representative morphologies can be described in low dimensional feature space; the first component represents elongation in the lateral direction and the second component represents anterior-posterior elongation and edges. Earlier studies have addressed a low dimensional embedding of morphology with milder variations [18,19,20–22,24,25]. The present results extend this approach to encompass more divergent morphologies commonly observed in different migratory cell-lines. Our new feature space essentially expanded the principle shape variations associated with cell polarity previously identified in the keratocyte data [18] to encompass narrowly polarized cell morphologies with pseudopodal extensions. The finding of low dimensional feature space is in line with an earlier feature representation of *Dictyostelium* cells based on Fourier-mode decomposition of the cell contour [20]. While being less analytically clear than the Fourier analysis, the present approach allows one to distinguish morphology with respect to the cell orientation and thus suited to analyze migratory cells. Since it did not take time sequence into consideration, a potential shortcoming of the analysis is that the comparision with real cells had to be amended with other dynamic features such as centroid movement and pseudopod splitting. Another downside is that application to cells with no clear orientation is not straightforward. Interestingly, sample conditions expected to alter cell polarity–vegetative stage *Dictyostelium*, *racE*-/*Dictyostelium*, and the nocodazole treated HL-60 cells were mapped to intermediate coordinates spanned by these two major manifolds. Moreover, the new sample conditions were each found clustered without significant overlaps with the training data sets. These results suggest that the region spanned by the three training datasets (keratocyte, H60, *Dictyostelium* (agg)) in the PC1-PC2 coordinate constitutes a space continuously occupied by forms realizable by genetic and phenotypic variations.

Search for the core regulatory logic behind cell deformation is important as it provides a much-needed basis to bridge the extracted shape features and the dynamics. Recent models have hinted at how the common shapes of migrating cells and their interconversions may come about (Table S in S1 Text). Excitable systems [14,37–40] describe pseudopod-like dynamics however at the expense of poor description of persistence; they exhibit unusually flat and periodically varying morphologies due to the wavefront dynamics (S1 Text; S4A–S4D, S9 and S10 Figs). Bistable models depicts polarity well, however simple addition of excitatory protrusions [42,53] do not recapitulate morphologies associated with formation of new pseudopods (S1 Text; S11 Fig, Tables T and U in S1 Text). These analyses show that a simple additive approach to complement the missing part is insufficient. Our model therefore takes into account the competition and feedback between the excitable protrusion and bistable polarization explicitly. Amplification of noisy signals through a feedback loop consisted of the *W*-dependent amplification of *V* (Eq 2C) and amplification of *W* by *V* (Eq 2B) supports de novo formation and splitting of pseudopods while maintaining the global cell polarity. These dynamics compete for dominance under limited *W*_tot_ i.e. the maximal protrusive activity. The resulting morphologies capture features that were not fully represented by earlier models (S1 Text).

Given its abstract nature, the ideal cell model is tailored to describe generic migratory morphologies observed across cell-types and species at the cost of being limited in predicting how exactly the coupling is implemented biochemically which may differ depending on the system. In neutrophils, excitable leading edge dynamics are governed by Cdc42 [32], and it also acts globally to enforce cell polarity by promoting actomyosin contractility through its effector WASP in a microtubule dependent manner [54]. In *Dictyostelium*, excitable dynamics at the leading edge is observed at the level of Ras which also interacts with GDP-bound form of RacE to strengthen cell polarity [36]. In this respect, mapping of the *racE*- morphology to a high *W*_tot_ state (Fig 5H) hints at the nature of the competition at least for *Dictyostelium* with regard to the states of F-actin: the contractile cortical meshwork that are crosslinked with myosin II or the protrusive dendritic meshwork that requires the Arp2/3 complex for side-branching nucleation. RacE is essential for plasma membrane localization of Diaphanous-related formins (DRFs) [51], and deletion of DRFs (ForA-/ForE-/ForH-) results in the loss of cortical actin. Since the morphology of the null mutant of DRFs phenocopies that of the *racE*-null cells [51], the increase in *W*_tot_ are associated with the absence of DRFs from the plasma membrane. On the other hand, the fluctuating protrusions are largely associated with fast idling pulses of Scar/Wave activities which are amplified by the excitable network [55]. Recent studies suggested that actin nucleators such as formins and Arp2/3 are competing for a limited pool of actin monomers and/or their upstream activators such as Rac-GTP [56–58]. Such notion is also supported by an observation that the amount of F-actin is compensated in Scar-/WASP- cells by increased localization of ForH at the cortex [59]. Taken together with our mapping of *racE*- data, these observations are in line with our current model view that excitability and bistable regulatory networks compete for dominance over limited *W*_tot_.

The variations in the distinct morphologies of differentiating *Dictyostelium* cells suggest alterations in the key parameters that serves as a control point. The difference between the vegetative- and aggregation-stage *Dictyostelium* was ascribed mainly to an increase in the membrane protrusion force *a*_w_. The increase in *a*_w_ can be understood from the fact that Rac1 [60] and SCAR [61], the essential factors for Arp2/3 activation, are known to be expressed at low levels in the vegetative-stage then increase markedly in the aggregation-stage cells. A recent study based on an excitable model [37] suggested a progressive state transition from a circular to amoeboid then to a keratocyte-like shape by the increase in the protrusion force (Fig 2F. in [37]). Rather, our model predicts that changes in the protrusive force should allow a direct transition from a circular shape (low PC1 low PC2) state to either amoeboid or keratocyte-like form depending on *W*_tot_. Such direct transition has been demonstrated experimentally and was attributed to an increased activity of a nested excitable network [14]. However, as discussed above, the elongated shapes simulated in their model were oscillatory and lacked persistency (S10 Fig). The presence of the polarity dynamics is essential for the persistent and longitudinally extended cell shapes. Relatively low *D*_w_ is required in both vegetative and aggregation-stage *Dictyostelium* to prevent the polarity dynamics from completely winning over the excitable dynamics. A highly polarized form at high *D*_w_; i.e. the 1-variable model limit *ζ/ D*w → 0 (S4E–S4H Fig; S1 Text) is indeed reached by cells that further differentiated into prespore cell-type (S4I Fig). While it is possible that certain cells are in a decoupled state (*ζ =* 0), the requirement of large *D*_w_ for cell polarity signifies the importance of *W* acting globally. Large *D*_w_ may not necessarily be mediated by pure diffusion as the present model postulates, but instead could be realized by other transport processes implicated in cell polarity such as membrane flow or the myosin-II dependent global actin flow. Global actin flow has been shown to maintain asymmetric distribution of de-filamenting factors [62,63], however such global flow may not always be present in polarized cells [64]. Since these transport processes are tied to cortical actin, they are naturally accompanied by changes in membrane tension [65] which should also be part of the feedback process from *W* to *V*.

Quantitative and systematic analysis of model outcomes will only increase its importance as we proceed further to unwind causality behind detailed geometries and dynamics associated with specific cells and conditions. The present framework of data analysis potentially provides means to test and improve specific models of migrating cells. For example, our ideal cell model gave rise to pseudopods from the tail region more frequently compared to the real *Dictyostelium* (agg) data. The discrepancy could be due to the fact that retraction was assumed to be driven only by the area conservation and that no regulated contractility was explicitly described. While this approximation can be justified when there is reciprocity between the front expansion and the rear contraction as has been shown to hold independently of actomyosin in neutrophils [50], the present model could be modified in the future to include local cortical actomyosin regulation when analyzing detailed shapes of the cell rear and the bleb-based front protrusion. Further improvement of the model and increasing dimensionality of the feature space may work hand in hand with extending the present analysis to classify morphologies exhibited by other cell types of wildtype and mutant backgrounds. For example, the present analysis fails to distinguish the pancake-like shape known for Rac and Rap related mutants that result from uniform expansion [44] and similarly round (i.e. low PC1, low PC2) cells inhibited of actin polymerization. This limit maybe overcome by introducing absolute size instead of normalizing the area so as to distinguish spherical cell versus flattened cell in two-dimensional cell masks. Expanding the analysis to 3-dimensional images would also be better suited to the present machine learning approach. As resolutions and dimensions are increased, the cell-shape based analysis may be supplemented with fluorescence image data of cytoskeletons and their regulators. Given the significant bottleneck in the present simulation by the huge computational loads which required parallel computation by GP-GPU, other avenues of coarse-graining maybe required to extend the present approach to a larger multi-modal analysis.

## Materials and methods

### *Dictyostelium* and HL-60 cell culture and data acquisition

Time-lapse data of freely migrating *Dictyostelium*, neutrophil-like HL-60, fish keratocyte and differentiated T mouse cells were acquired with an inverted microscope using either 20, 40 or 60x objective lens. For *Dictyostelium* and HL-60, cells expressing Lifeact fused to mNeonGreen [66] and mTurquoise2 were employed, respectively. A Lifeact-mTurquoise2 expression vector was constructed by ligating Lifeact-mTurquoise2 into an episomally replicating plasmid pEBMulti Neo (WAKO, 057–08131) at restriction sites *Xho*I and *Not*I. The Lifeact-mTurquoise2 expressing stable HL60 cell line was obtained by introducing the plasmid by electroporation (NEPA21; Nepa Gene, Ltd., Chiba, Japan) followed by G418 selection (1 mg mL^–1^) after 2 days. For fish keratocytes, DIC images were employed. *Dictyostelium* cells were grown axenically and obtained according to standard protocols as previously described [67]. Vegetative *Dictyostelium* AX4 cells (N = 1694 snapshots from 18 timeseries;), aggregation-stage *Dictyostelium* AX4 cells (starved for 3.5 hours; N = 2841 snapshots 19 timeseries;), vegetative LifeactGFP/*rac*E- cells (N = 330 snapshots from 8 timeseries;) were plated on a non-coated coverglass and images were acquired at 5 sec/frame (aggregation-stage AX4 and *rac*E- cell) or 15 sec/frame (vegetative AX4). Neutrophil-like HL-60 cells were grown in RPMI1640/glutamate media (Wako 189–02145) supplemented with 12% FBS (Sigma 172012). Differentiated HL-60 cells were obtained by treating the cells with DMSO for 3 days. Images of HL-60 cells on fibronectin-coated glass plates in the presence of 1 nM fMLP (N = 3468 snapshots from 23 timeseries;) were taken at 5 sec/frame. For nocodazole treatment, differentiated HL-60 cells were collected by centrifugation, suspended in fresh HBSS containing 20 μM nocodazole and plated on a coverslip pre-coated with 1–2% BSA in PBS. Data were acquired within 20–75 min in the same medium in the presence of nocodazole (N = 181 snapshots from 3 timeseries).

### Primary cell culture and data acquisition

Keratocytes from the scales of Central American cichlids (*Hypsophrys nicaraguensis*) were cultured as previously described [68] and images were recorded at 2-s intervals (N = 1590 snapshots from 12 timeseries;). Naïve CD4^+^ T cells were isolated from the lymph nodes and spleen of C57BL/6 mice by MidiMACS (Miltenyi Biotec). Cells were activated by plate-bound anti-CD3 (5 μg mL^–1^) and anti-CD28 (2.5 μg mL^–1^) with cytokines and blocking antibodies. Th1: 2 ng mL^–1^ hIL-2, 5 ng mL^–1^ mIL-12. Treg: 1 ng mL^–1^ hTGFβ, 1 μg mL^–1^ anti-IFNγ. Snapshots from these timelapse recording were employed for feature extraction.

### Deep-learning-based feature extraction

Mask images were pre-processed as follows (Fig 1A, upper panel): (i) the migration direction was determined from the centroid displacement at a five timeframe interval (equivalent to 25 sec for aggregation-stage and *rac*E- *Dictyostelium* data, 10 sec for keratocyte data) except for vegetative *Dictyostelium* data where 1 timeframe (equivalent to 15 sec) was used, (ii) binarized mask image was rotated to align the migration direction to the y-axis, (iii) the image was rescaled so that the cell area is equal to the area of a circle with 25 pixel diameter. The rescaled masks were each embedded at the center of a blank square frame of 64x64 pixels. The exact spatial resolution of mask images varied from sample to sample due to rescaling, however they were all in the order of ~0.5 μm/pixel. Convolutional neural network (Fig 1A, bottom panel) was implemented using Keras (https://keras.io) with TensorFlow backend. To make the sample size of the three datasets near equal, data augmentation was performed by rotating the original masks at angles (< ±5 deg) randomly picked from a uniform distribution (see Table A in S1 Text for the number of samples). Input vectors were processed through layers of convolution operation (Fig 1A, bottom panel; ‘convolution’) in addition to layers of max pooling operation with a 3x3 kernel to render the analysis robust to positional deviation (Fig 1A, bottom panel; ‘pooling’). These were then processed through a set of densely connected layers with rectified linear and hyperbolic tangent activation function (Fig 1A, bottom panel; ‘rel’ and ‘tanh’). In the final layers, the dimension of the vector was reduced to three and were passed to ‘softmax’ activation function. The values of the three nodes (*F*_1_, *F*_2_, *F*_3_) before the final softmax layer were employed to represent cell shapes. The number of training epoch was 2000 which was sufficient for adequate learning as determined by the accuracy and loss values (S1A and S1B Fig). The vector representing three nodes ***F*** = (*F*_1_, *F*_2_, *F*_3_) was further reduced in dimension by principal component analysis (PCA). The PCA parameters were acquired from the average ***F*** of 54 timeseries in total (19, 23, and 12 samples for *Dictyostelium*, HL-60 and keratocyte, respectively).

### Geometrical analysis of feature space

To examine mapping of 2-dimensional geometries in the feature space, 7 well-defined objects were used; circle, isosceles triangle, asymmetric right triangle, rectangle, rhombus, pentagon and star polygon in a specified orientation (Fig 1N). Triangles, pentagon and star were flipped vertically to obtain total of 11 basic objects. The library was further expanded to 33 shapes by including variants of the basic geometries with different aspect ratio 1:1, 1:2 (Fig 1N red, horizontally elongated shape to the moving direction) and 2:1 (Fig 1N blue, flattened shape with vertically elongated to the moving direction).

### Model equations

To numerically simulate the interface between the plasma membrane and the extracellular space, we employed a phase-field equation in the following form [42,69,70].


$$\tau \frac{\partial \phi }{\partial t}=\eta \left(\Delta \phi -\frac{1}{\varepsilon^{2}}G\prime (\phi )\right)-M\left(\int_{\mathrm{cell}}\phi dr-A_{0}\right)|\nabla \phi |+F_{\mathrm{prot}}(\left\{W\right\})|\nabla \phi |$$ (1)

The equation describes the dynamics of a continuous state variable *ϕ*(***r***;*t*) in a two-dimensional space that specifies whether a position ***r*** is occupied (*ϕ* = 1) or not occupied (*ϕ* = 0) by a cell at time *t*. In the present study, we consider initial conditions with a single continuous domain with *ϕ* = 1. The parameter *τ* is the viscous friction coefficient. The first term of the r.h.s represents effective surface tension, where Δ*ϕ* and *G* are derived from membrane energy. *G* is Landau functional describing a bi-stable potential. Here we chose *G*(*ϕ*) = 18*ϕ*^2^(1−*ϕ*)^2^ [69]. The second term describes restoring force that keeps the cell area close to *A*_0_. The Eq 1 assumes that the bending energy is negligible [70]. The third term represents active force with magnitude *F*_prot_ that is perpendicular to the boundary |*∇ϕ*|≠0 and thus drives membrane extension. In the present simulations, parameters in Eq 1 were set so that they are an order of magnitude within generally accepted values (Table C in S1 Text); surface tension *η* = 1.0 [pN], [71], cell area *A*_0_ = 78.83 [μm^2^] (~ 5 μm radius circle), protrusive force by actin polymerization *a*_w_*W* = 0.8–4.0 [pN/μm] (for *W* ~ 1) [72,73]. Since *τ* was not well constrained experimentally and expected to differ between cell-types and the culture conditions, we adopted an empirical value 0.83 [pN/μm^2^] [70], which was then calibrated retrospectively by a multiplier τ*’* so that the time scale of the simulated cell trajectories match with that of real data as described in the later section. The area constraint *M* = 0.5 pN/μm^3^ and the size of the boundary layer *ε* = 1.0 [μm] were set close to those in the earlier studies [69,70].

The ‘ideal cell’ that can take close to all of the basic phases of morphology features that we have examined (Fig 1) should consist of two main features: 1) transient appearance of localized protrusions and 2) prolonged presence of single expanding edge and retracting tail to appear under homogeneous extracellular conditions [1]. Here, we formulate a reaction-diffusion model that describes these two processes mathematically as follows:

$$\tau \prime \frac{\partial (\phi U)}{\partial t}=\phi \left(-\frac{\alpha UVW}{K_{k}+\frac{\int dr\phi VW}{\int dr\phi }}+\frac{\beta UV}{K_{p}+\frac{\int dr\phi U}{\int dr\phi }}+s-\gamma U\right)+D_{U}\nabla ∙(\phi \nabla U)-\phi N(r,t)-\chi_{U}\frac{U{\left|\nabla \phi \right|}^{2}}{\int dr{\left|\nabla \phi \right|}^{2}},$$ (2A)

$$\tau \prime \frac{\partial (\phi V)}{\partial t}=\phi \left(\frac{\alpha UVW}{K_{k}+\frac{\int dr\phi VW}{\int dr\phi }}-\frac{\beta UV}{K_{p}+\frac{\int dr\phi U}{\int dr\phi }}-\mu V\right)+D_{V}\nabla ∙(\phi \nabla V)+\phi N(r,t),$$ (2B)

$$\tau \prime \frac{\partial (\phi W)}{\partial t}=\phi (k_{W1}(-\rho W^{3}+\rho W^{2}W^{*}-W)+\zeta V)+D_{W}\nabla ∙(\phi \nabla W).$$ (2C)

where the first two equations for *U* and *V* describe an excitable reaction network for transient protrusive dynamics, and the third equation for *W* describes polarization dynamics, respectively (Fig 2A; S1 Text). The role of the excitable reaction network is to generate transient signals for local protrusions in a minute to a few minutes time-scale by amplifying small edge fluctuations of seconds order [55]. In neutrophils, excitable dynamics of Cdc42 and PI3K activity [32,33] is essential for front protrusions. In *Dictostelium*, excitable dynamics are observed at the level of spontaneous Ras and PI3K activation [30,70,74]. Here, we adopted equations originally introduced to study excitable PI3K activities and the resulting F-actin waves in *Dictyostelium* (Eqs 2A and 2B) [70]. Parameters *α* and *β* are the rate constants of reaction *U* → *V* and *V* → *U* multiplied by the time-scale factor *τ*′. The source of edge fluctuation is introduced as a noise term *ϕ N*(***r***,*t*) [70] in Eq 2B which is amplified through *V* by a positive-feedback described in the first term. Increase in *V* is then slowed down due to depletion in *U*, and the system eventually recovers the original resting state.

The expanding membrane region is determined by *W* governed by Eq 2C (Fig 2A; see S1 Text for derivation) which is similar in form to the wave-pinning [29]. The same type of equation has been used earlier to study polarized cell shape in fish keratocyte [75] and *Dictyostelium* [42]. The first term describes reaction kinetics with bistability, and the second term describes diffusion. We assume that the sum of *W* and its reciprocal state W* is conserved (*W*_tot_ = const.) [29] and that W* diffuses much faster than *W* and thus can be approximated as uniform in space. Hence we obtain

$$W^{*}=\frac{W_{\mathrm{tot}}-\int dr\phi W}{\int dr\phi }$$

Due to the global constraint, this class of bistable system gives rise to coexistence of low and high *W* regions due to stalling of a transitory wave front—a property known as “wave pinning” [29,75]. For the sake of brevity, we shall embed rear retraction passively in the form of restoring force. Thus we set *F*_prot_ = *a*_W_*W* in Eq 1 [69] so that the edge expands where *W* is high, and the rest of the domain with high *W*_tot_ /*A*_0_ -*W* as a result contracts due to area conservation imposed by the second term in Eq 1. Note that *W* only specifies where the protrusions and retraction take place and does not assume the origin of their driving force. The variable *W* can thus be interpreted as an abstract representation of bistable signals such associated with the leading edge of polarize cells as Rac-GTP, or alternatively, *W*_tot_/*A*_0_-*W* with the reciprocal spatial profile to indirectly represent signals that regulate rear contraction such as RhoA-GTP. A recent study has shown that Rac-GTP accumulates at the front of migrating neutrophils regardless of whether it is F-actin filled or blebbing [65]. Based on the inherent ability to maintain persistent front-and-back unipolarity, the polarity network (Eq 2C) serves as a spatio-temporal filter to select or remove local protrusions. This coupling is described by assuming that *W* positively regulates the positive-feedback amplification of *V* (Eq 2C; r.h.s first term) in addition to *W* feeding back to increase *V* (Eq 2A) to reflect F-actin- or tension-dependent positive regulation of leading edge signals in neutrophils and *Dictyostelium* [65,76,77].

### Parameter search

Model parameters were selected for the systematic feature analysis (Fig 3A; Table G in S1 Text) based on the following criteria: Of the total 22 parameters (Table C and D in S1 Text), eight parameters (*W*_tot_, *a*_W_, *k*_W1_, *μ*, *γ*, *ρ*, *D*_W_, *χ*_U_) were chosen for detail analysis. Parameters (*α*, *β*, *K*_*k*_, *K*_*P*_, *s*) that define the kinetics of *U*, *V* were fixed to those used earlier [70]. Two to four independent simulations from different random seeds were executed for a given parameter set to obtain average feature scores ***F***. Because grid-search in 8 dimensional parameter space was unattainable due to heavy computational load, we adopted parameter search around manually selected reference parameter sets. First, based on preliminary simulations performed with various parameters, a single parameter set R1 was chosen which gave rise to polarized cell morphology; i.e. elongated shape in the moving direction. Around the reference set R1, the following parameters were varied: *a*_w_ and *W*_tot_ which appeared to affect the elongation of cell in the moving direction,*χ*_U_ and *D*_W_ related to the split of the leading edge, and *γ* that seems to affect the appearance of new pseudopods. We performed a grid search around R1 in 2-dimension (*a*_W_, *γ*) for *χ*_U_ = 0 and *χ*_U_ = 50 (S5A Fig left panels) while fixing other 6 parameters. Similarly, a grid search was performed in (*W*_tot_, *D*_W_) for *χ*_U_ = 0 and 50 (S5A Fig left panels). Second, we chose another parameter set R2 which gave rise to relatively round shape and random cell displacement (i.e. near HL60-like behavior). Around R2, we varied (*a*_W_, *W*_tot_), (*γ*, *ρ*), and (*k*_W1_, *μ*) at both *χ*_U_ = 0 and 50 (S5A Fig right panels). Representative results of the simulations are shown in S5B and S5C Fig. Phase diagram of feature vector ***F*** around R1 on the *γ*-*a*_W_ plane and around R2 on *μ* - *k*_W1_ plane are shown in S5D and S5E Fig, respectively.

### Pseudopod analysis

For both experiments and simulation data, events of pseudopod formation were detected manually by eye as either ’de novo’, ’Y-split’ or ‘one-way-split’ based on [7]. New pseudopods that appeared in non-protruding regions (Fig 4E left) were counted as "de novo formation”. Protrusions that appeared close to the leading edge were marked as ‘Y-split’ because of the resulting cell shape (Fig 4E right). When new protrusive events occurred on top of the leading edge so as to offset and steer the extending pseudopod, it was counted as ‘one-way-split’ (Fig 4E middle) [7]. For the subset of the data, the analysis was performed three independent times to confirm reproducibility. To obtain angular distribution, direction of pseudopod extension 10 seconds from the onset was measured relative to the centroid movement. Aggregation-stage *Dicyostelium* (N = 90 from 4 timeseries), and the closest simulations (N = 44 from 2 timeseries). HL-60 (N = 68 from 5 timeseries), and the closest simulations (N = 77 from 3 timeseries).

## Supporting information

S1 Text The online supporting information includes. 1. Details of our mathematical model. 2. Feature extraction from the intermediate-layer. 3. Analysis based on commonly used intuitive features. 4. Details of related mathematical models.
(DOCX)

S1 Fig Image classification of migratory cells based on a deep convolutional neural network provides highly compressed representation of the overall cell shape, protrusions and their orientation. (A, B) The values of accuracy (A) and loss (B) during training (blue) and validation (red) of the deep convolutional neural networks. (C-E) Representative time series of the feature vector ***F*** for *Dictyostelium* (agg)(C), HL60 (D) and fish keratocyte (E). (F, G) Orientation dependency for the oval shape (Fig 1(M)) *L* = 0.75*L*_0_ (F) and *L* = *L*_0_ (G). (H-J) Mapping of circles and polygons with various aspect ratios (x-axis: y-axis) 1:3, 2:5, 1:2, 2:3, 1:1, 3:2, 2:1, 5:2 and 3:1. Circles, squares and rhombuses (H), triangles, pentagons and star shapes in the upright (I) and inverted (J) orientation. (K) Orientation dependency of a complex shape with multiple edges.
(TIF)

S2 Fig Analysis of hand-crafted morphological features. (A, B) Definition of *h*_1_ (A) and *h*_2_ (B). The first feature *h*_1_ and the second feature *h*_2_ is the degree of elongation parallel and orthogonal to the front-tail axis direction. Those values were extracted using the normalized mask image. (C) Definition of *h*_3_. The third feature *h*_3_ is circularity of a cell mask. (D) PC1-PC2 diagram obtained from each snapshots using hand-crafted features (left). The same diagram obtained using DNN-based features was also shown for comparison (right). The aggregation-stage *Dictyostelium*, HL-60, and fish keratocyte were shown as dark red, dark red, and yellow colors.
(TIF)

S3 Fig Analysis of real cell data with varying degree of bifurcating protrusions indicates polar and multiple edge representation in PC2. (A, B) Mapping of cell shape with anterior-posterior elongation without lateral pseudopods (*Dictyostelium* prespore cell-type). Cell-to-cell variation (A) and a representative temporal variation of a single cell (B). Blue circled regions represent 95% confidence eclipses for the mean of all combined timeseries (dotted) and the mean of individual cells (filled). (C, D) Mapping of mouse T cells in the PC1-PC2 space. Filled rhombuses: T helper 1 (C) and regulatory T cell (D). Circled regions in the background indicate the reference data in Fig 1B (aggregation-stage *Dictyostelium* dark green (dark green), HL60 (dark red) and fish keratocyte (yellow)).
(TIF)

S4 Fig Model behaviors in the 2-variable and 1-variable limit shows other repertoires in the morphodynamics. (A-D) The 2-variable scheme (A) and its morphology dynamics (B-D). Representative simulations showing traveling patches (B) and lamellipodium-like protrusions (C). Color overlay; red *V*, green *U*. (D) Time series of the local curvature of the boundary (left panel) and local elongation (right panel) for (C). (E-G) The 1-variable scheme (E) and its representative morphology dynamics (F, G). Blue indicates the high *W* region. (H) Time series of the local curvature at the boundary (let panel) and local elongation (right panel) for (G). (I) Feature mapping of the polar morphology in 1-variable scheme. Time average (solid circles) and time samples (filled). Right panels in colored frames indicate representative snapshots. See Table O in S1 Text for parameter values.
(TIF)

S5 Fig Sampled parameter conditions. (A) Two-dimensional grids around manually selected reference parameters R1 (upper left panels; *χ*_U_ = 0), R1’ (lower left panels, *χ*_U_ = 50), R2 (upper right panels) and R2’ (lower right panels). Each square in the grid represents a sampled parameter condition. (B, C) Representative simulation time series for parameters R1, R2’ and (1)-(8) in (A). (D) Feature scores (*F*_1_, *F*_2_, *F*_3_) in the (*γ-a*_W_) plane around R1. (E) Feature scores (*F*_1_, *F*_2_, *F*_3_) in the (*k*_W1_-*μ*) plane around R2’. See Table O in S1 Text for parameter values.
(TIF)

S6 Fig Parameter dependency of the simulated morphology. Dependency on *γ*, *ρ*, *D*_W_, *χ*_U_ of the simulated morphology (A, C, E, G) and the feature values (B, D, F, H). See Table O in S1 Text for parameter values.
(TIF)

S7 Fig Reducing the noise term improves high Score-K simulations. (A, B) Shape and orientation fluctuations observed under the default noise size (*θ =* 1.4, *σ* = 0.075) (A). Mapping in PC1-PC2 plane in Score-K rank1 simulation (B). (C, D) The same analyses at (*θ =* 14, *σ* = 0.0075).
(TIF)

S8 Fig An intermediate-layer of the convolutional neural network yields a similar feature space. (A) PCA of the 256 dimensional intermediate layer. Microscopy dataset of *Dictyostelium* (agg) (green +), HL-60 (dark red +), keratocyte (yellow +), *Dictyostelium* (veg) (magenta inverted triangles), *Dicytostelium racE*- strain (red squares) and Nocodazole-treated HL-60 (blue triangles). (B) Mapping of model simulations (grey circles), the top-ranking simulations based on the feature vector ***F*** (dark green circle for *Dictyostelium* (agg), magenta circle for *Dictyostelium* (veg)) and those based on 256-dimensional features (light green circle for *Dictyostelium* (agg), pink circle for *Dictyoste* the *lium* (veg)). (C, D) Time-series of simulations with the highest feature similarity to *Dictyostelium* (aggregation-stage) (C), vegetative *Dicyostelium* (D). See Table O in S1 Text for parameter values.
(TIF)

S9 Fig Morphology features in previous excitable models. (A, B) The model by Bhattacharya et al [40]. The PC1-PC2 diagram was plotted based on snapshots taken from S4 Movie (red circle) and S5 Movie (blue circle) in [40] (A). Local boundary curvature (top) and the protrusion speed (bottom) obtained from S5 Movie in [40] (B). (C, D) The model by Cao *et al*., 2019 [37]. PC1-PC2 diagram was plotted based on snapshots from Video 2 (red circle) and Video 3 (blue circle) in [37] (A). Local boundary curvature (top) and the protrusion speed (bottom) obtained from Video 2 in [37] (B).
(TIF)

S10 Fig Feature mapping of a fluctuating fan-shaped cell. (A) A representative simulation result of a fan-shaped cell with a fluctuating front. Color overlay; red *V*, green *U*. (B) Feature mapping: present model (triangle), the PIP2-modulated cell (Video 4 in [14]) (circle) and an earlier model (Video 13 in [14]) (cross). (C) A kymograph of the variable *V* at the cell edge taken from the simulation in (A). Arrows indicate bifurcating V-shaped fronts. (D) Kymographs of the local curvature (top panels) and the protrusion size (bottom panels) along the cell boundary; for the real cell data (Video 4 in [14]) (left), and an earlier model simulation (Video 13 in [14]) (middle), and the present model (A) (right).
(TIF)

S11 Fig Morphology features in Moreno *et al*. [52]. (A) Mapping of simulated cell morphology from 105 parameter sets of the model (blue circle). The results from our model (black circle) shown for comparison. (B) Mapping of individual snapshots of the *Dictyostelium* (agg) data (left), Score-D(agg) rank1 simulations in the Moreno *et al*. (middle) and our model (right). (C) Mapping of individual snapshots of HL-60 data (left), Score-H rank1 simulations in the Moreno *et al*. (middle) and our model (right). (D) Proportion of de novo pseudopod formation in real cell data and the rank1 simualtions; Dictyostelium (agg) (left lanes) and HL-60 (right lanes).
(TIF)

S1 Movie Timelapse sequence of representative training data images; *Dictyostelium* (agg), HL60 and fish keratocyte (from left to right).
(MP4)

S2 Movie Parameter dependency (*W*_tot_) of the morphodynamic model (Fig 2D). (MP4)

S3 Movie Parameter dependency (*k*_w1_) of the morphodynamic model (Fig 2F). (MP4)

S4 Movie Parameter dependency (*a*_w_) of the morphodynamic model (Fig 2H). (MP4)

S5 Movie Parameter dependency (*μ*) of the morphodynamic model (Fig 2J). (MP4)

S6 Movie Cell morphology dynamics of aggregation stage *Dictyostelium* and the corresponding top ranking simulations. (MP4)

S7 Movie Cell morphology dynamics of HL60 and the corresponding top ranking simulations. (MP4)

S8 Movie Cell morphology dynamics of fish keratocyte and the corresponding top ranking simulations. (MP4)

S9 Movie Cell morphology dynamics of vegetative stage *Dictyostelium* and the corresponding top ranking simulations. (MP4)

S10 Movie Cell morphology dynamics of nocodazole-treated HL60 and the corresponding top ranking simulations. (MP4)

S11 Movie Cell morphology dynamics of *Dictyostelium* racE- and the corresponding top ranking simulations. (MP4)
