# Supplementary material for: Comparative mapping of crawling-cell morphodynamics in deep learning-based feature space
Source: PLoS Comput Biol. 2021 Aug 12;17(8):e1009237. doi: 10.1371/journal.pcbi.1009237 (PMC8360578; doi:10.1371/journal.pcbi.1009237)
Supplement: S1 Text — 1. Details of our mathematical model. 2. Feature extraction from the intermediate-layer. 3. Analysis based on commonly used intuitive features. 4. Details of related mathematical models. (DOCX) [file pcbi.1009237.s001.docx]

**Supporting Information for**

**Comparative mapping of crawling-cell morphodynamics in**

**deep learning-based feature space**

Daisuke Imoto^1^, Nen Saito^2,3^, Akihiko Nakajima^1,4^, Gen Honda^1^, Motohiko Ishida^1^, Toyoko Sugita^1^,

Sayaka Ishihara^5^, Koko Katagiri^5^, Chika Okimura^6^, Yoshiaki Iwadate^6^ and Satoshi Sawai^1,2,4,7*^

**1** Department of Basic Science, Graduate School of Arts and Sciences, University of Tokyo, Tokyo, Japan, **2** Universal Biological Institute, University of Tokyo, Tokyo, Japan, **3** Exploratory Research Center on Life and Living Systems, National Institutes of Natural Sciences, Okazaki, Japan, **4** Research Center for Complex Systems Biology, Graduate School of Arts and Sciences, University of Tokyo, Tokyo, Japan, **5** Department of Biosciences, School of Science, Kitasato University, Sagamihara, Japan, **6** Faculty of Science, Yamaguchi University, Yamaguchi, Japan, **7** Department of Biology, Graduate School of Science, University of Tokyo, Tokyo, Japan

*Corresponding author: cssawai@mail.ecc.c.u-tokyo.ac.jp

1. **Mathematical model**

**1-1. Model equations**

The reaction scheme for *U* and *V* describes two counteracting reactions U → V and V → U mediated by enzymes A and B. We adopted an earlier model [70] with a minor modification to include a feedback from an additional polarity factor W; namely,

$$\frac{\text{d}A}{\text{d}t}=k_{1}WVA_{cyt}-k_{2}A$$

$$\frac{\text{d}B}{\text{d}t}=k_{3}UB_{cyt}-k_{4}B$$

$$\frac{\text{d}V}{\text{d}t}=k_{5}AU-k_{6}BV-k_{7}V$$

$$\frac{\text{d}U}{\text{d}t}={-k}_{5}AU+k_{6}BV+k_{8}-k_{9}U$$

where *A* and *B* are the concentrations of enzymes in the membrane-bound form that mediate the reaction U → V and V → U, respectively. Originally, the forward reaction from *U* to *V* represented phosphorylation of the phosphatidylinositol (4,5)-bisphosphate (PIP2) to phosphatidylinositol (3,4,5)-triphosphate (PIP3) mediated by PI3kiase and the reverse reaction mediated by PTEN phosphatase, and the positive feedback amplification of *A* described the F-actin dependent PI3K activation. Due to the introduction of new polarity variable *W*, the amplification rate for A in the present model assumed it to be proportional to *VW* instead of *V*^2^ [70]. *A*_cyt_ and *B*_cyt_ represent the respective cytosolic fractions. Parameters *k*_1_ through *k*_9_ are the rate coefficients. We assume that *A*_cyt_ and *B*_cyt_ diffuse fast so that they are distributed uniformly in space and that the sum of membrane-bound and cytosolic fractions are conserved, $A_{\mathrm{cyt}}\int_{\text{Ω}} \phi{\text{d}\boldsymbol{r}}+\int_{\text{Ω}} \phi A{\text{d}\boldsymbol{r}}=A_{\mathrm{tot}}\int_{\text{Ω}} \phi{\text{d}\boldsymbol{r}}$ and $B_{\mathrm{cyt}}\int_{\text{Ω}} \phi{\text{d}\boldsymbol{r}}+\int_{\text{Ω}} \phi B{\text{d}\boldsymbol{r}}=B_{\mathrm{tot}}\int_{\text{Ω}} \phi{\text{d}\boldsymbol{r}}$. Ω represents the entire spatial region computed. Provided that the reaction for *A* and *B* is fast compared to those of *U* and *V* [70], we obtain Equations (2a) and (2b) by combining the reactions with molecular diffusion in the phase-field *φ* (diffusion coefficients for *U* and *V* are $D_{U}$ and $D_{V}$) and the absorption of *U* at the cell boundary represented by the last term of Eq. (2a). Parameters in Eqs. (2a) and (2b) are *α* = *k*_5_*A*_tot_, *β* = *k*_6_*B*_tot_, *K*_k_ = *k*_2_/*k*_1_, *K*_p_ = *k*_4_/*k*_3,_ * = k*_7,_ *s = k*_8_ and γ = *k*_9._

For the time evolution of *W*, we adopted an interconversion process between the active form *W* and the inactive form *W** under both positive and negative feedback regulation (S4 Fig).

$$\frac{\text{d}X}{\text{d}t}=\kappa_{1}W^{2}-\kappa_{2}X$$

(S2-1)

$$\frac{\text{d}Y}{\text{d}t}=\kappa_{3}+\kappa_{4}W^{2}-\kappa_{5}Y$$

(S2-2)

$$\frac{\text{d}W}{\text{d}t}=\kappa_{6}XW^{*}-\kappa_{7}YW$$

(S2-3)

where X mediates the reaction $W\to W^{*}$ and Y mediates $W^{*}\to W$*.* By assuming sufficiently fast relaxation for *X* and *Y* compared to *W*, we adiabatically eliminated the dynamics for *X* and *Y* (d*X*/d*t* = 0 and d*Y*/d*t* = 0) so that

$$\frac{\text{d}W}{\text{d}t}=k_{W1}(-\rho W^{3}+\rho W^{*}W^{2}-W)$$

(S2-4)

where $k_{W1}= \kappa_{3}\kappa_{7}/\kappa_{5}$ and $\rho=\kappa_{4}/\kappa_{3}{=\kappa}_{1}\kappa_{5}\kappa_{6}/(\kappa_{2}{\kappa_{3}\kappa}_{7})$. By further assuming that *W* is positively regulated by *V* following first-order kinetics and coupling the reactions with molecular diffusion in the phase-field *φ* [70], we obtained Eq. 2-2c which is similar in form to the cell polarity model [29] derived from somewhat more detailed bistable regulation of Rho-GTPase. Other related studies [42, 75] have also employed similar equations.

Under conditions where *W* becomes enslaved to *V* (*ρ* → 0, *D*_w_ → 0; Eq. 2c), the system is reduced to the 2-variable model [70] (Eq. 2A,B; Figs S4A, S4B, S4C, and S4D). Similarly when activation of *W* by *V* is diminished (*ζ* → 0; Eq. 2c), the system becomes the 1-variable cell polarity model (Figs S4E-S4H). In the 2-variable regime, depending on the boundary conditions, excitable waves propagated either globally [70] or as randomly nucleated patches at the edge (S4B Fig). These protrusions were always localized in space and time and did not give rise to persistently polarized cell morphology. On the other hand, the 1-variable model exhibited polarized morphologies and new protrusions grew only in the rear and not in the anterior region (S4G and S4H Figs). Moreover, the average morphologies in the 1-variable regime (S4I Fig; small circles) were displaced towards higher PC1 from the *Dictyostelium* (agg) data (S4I Fig; oval regions) and thus mapped near the non-bifurcating ellipsoidal shapes analyzed in Fig 1M.

**1-2. Numerical calculation**

Numerical calculation was performed by a semi-implicit type of explicit method [75]. First, the updated values for $\phi$, $\phi V$, $\phi U$and $\phi W$are calculated as follows,

$${(\phi V)}_{t+\Delta t}=\phi_{t}V_{t}+\Delta t\left( \frac{\partial(\phi V)}{\partial t} \right)$$

(S3-1),

$${(\phi U)}_{t+\Delta t}=\phi_{t}U_{t}+\Delta t\left( \frac{\partial(\phi U)}{\partial t} \right)$$

(S3-2),

$${(\phi W)}_{t+\Delta t}=\phi_{t}W_{t}+\Delta t\left( \frac{\partial(\phi W)}{\partial t} \right)$$

(S3-3),

$$\phi_{t+\Delta t}=\phi_{t}+\Delta t\left( \frac{\partial\phi}{\partial t} \right)$$

(S3-4).

Next, if $\phi_{t}$ is larger than th*_φ_*, *V*, *U* and *W* are updated following

$$V_{t+\Delta t}=\frac{{(\phi V)}_{t+\Delta t}}{\phi_{t+\Delta t}}$$

(S3-6),

$$U_{t+\Delta t}=\frac{{(\phi U)}_{t+\Delta t}}{\phi_{t+\Delta t}}$$

(S3-7),

$$W_{t+\Delta t}=\frac{{(\phi W)}_{t+\Delta t}}{\phi_{t+\Delta t}}$$

(S3-8),

otherwise

$$V_{t+\Delta t}={(\phi V)}_{t+\Delta t}$$

(S3-9),

$$U_{t+\Delta t}={(\phi U)}_{t+\Delta t}$$

(S3-10),

$$W_{t+\Delta t}={(\phi W)}_{t+\Delta t}$$

(S3-11).

The value of th*_φ_* was set to 10^−4^. The time increment (Δ*t*) and the spatial grid (Δ*x*) are set to Δ*t* = 4×10^−5^ and Δ*x* = 0.1. The program was coded in C++ with Open ACC (PGI compiler) for GPGPU computation (NVIDIA GeForce 1080Ti). The noise terms in Eq. 2a and 2b were given by $N\left( \boldsymbol{x,}t \right)=N_{0}exp(-\left| \boldsymbol{x}-\boldsymbol{x}_{c} \right|^{2}/2d^{2})$, where $\boldsymbol{x}_{c}$ is a random position selected at rate $\theta$ per area and *d* is the nucleation size. The noise amplitude $N_{0}$ follows an exponential distribution with the average σ. In case *N*(***r***,*t*) exceeds *U*(***r***,*t*), *N*(***r***,*t*) was reset to *U* so as to prevent *U* from taking negative values*.* Parameters were fixed to *θ* = 1.4, *d* = 0.8, *σ* = 0.075 for all calculations on full 3-variable reaction regime. For 1-variable and 2-variable regime, σ was varied.

**2. Feature extraction from the intermediate-layer of the trained neural network**

To check robustness of the feature mapping, we revisited the morphology analysis using the 256-dimension intermediate-layer of the trained convolutional neural network (Fig 1A). This was to check that important shape features useful to further constrain the model parameters were not lost in the final layer of the convolutional network. Starting from the 256 dimensional feature, we found that PCA yielded almost identical shape representation in two dimensional space. The contribution of the first two principle components PC1, PC2 and the sum of the all other components (PC3~PC256) were approximately 61.4%, 34.2% and 4.4%, respectively. Real dataset were clustered in the feature space accordingly to their respective attributes (S8A Fig). This new space was very similar to the one obtained from the last layer in terms of the relative positioning of the trained data. The vegetative *Dictyostelium* data are located between HL-60 and aggregation-stage *Dictyostelium,* and Nocodazole-treated HL-60 data are located between HL-60 and keratocyte, whereas *Dictyostelium racE*- data are mapped between aggregation-stage *Dictyostelium* and keratocyte. Mapping of simulated cell morphologies are shown in S8B Fig. Parameter sets were ranked according to the Euclidean distance in the 256 dimensional feature space between the simulated morphology and the mean features of the reference real data. In case of HL-60, keratocyte, nocodazole-treated HL-60 and *Dictyostelium* *racE*- data, top ranking simulations were identical to those obtained using the feature vector ***F*** described in the main text (Figs 3F, 3I, 5E, and 5G). The top ranking simulations for aggregation-stage *Dictyostelium* and vegetative *Dictyostelium* based on the 256-dimensional features (Figs S8C and S8D) were different (Table M) but still within a very close range in the feature space (S8B Fig). These results indicate that the measure of similarity does not depend largely on whether the feature is based on the intermediate or the final layer of the trained network.

**3. Analysis based on hand-crafted features**

The ability of the proposed Deep Neural Network (DNN)-based features to characterize the three representative morphologies was compared to traditional hand-crafted features extracted from the same normalized mask images. The first and second features *H*_1_ and *H*_2_ represent the degree of elongation ‘orthogonal’ (S2A Fig) or ‘parallel’ (S2B Fig) to the front-tail axis direction respectively. The third feature *H*_3_ was ‘circularity’ (= 4π*A*/*P*^2^ ) of a cell mask with area *A* and perimeter *P*. The features *H*_i_ were normalized as follows:

$$h_{i}=(H_{i}-\mu_{i})/\sigma_{i}$$

(S4-1).

where, *μ*_i_ and *σ*_i_ are the mean and the standard deviation, respectively (Table P). For comparison, the feature vector ***h*** = (*h*_1_, *h*_2_, *h*_3_) was reduced in dimensionality by PCA. The latency values of the three principal components PC1, PC2 and PC3 were approximately 59.8%, 38.3% and 1.9%, respectively similar to those using the DNN-based features (66.3%, 33.5% and 0.3%).

Mapping of individual snapshots in the PC1-PC2 space showed that there were larger overlaps between *Dictyostelium* and HL-60, *Dictyostelium* and keratocyte data in the hand-crafted feature space (S2D Fig left panel) compared to the DNN-based feature space (S2D Fig right panel). In other words, the three morphologies were more equally spaced and better separated in the DNN-based feature space than in the traditional hand-crafted features. For further comparison, the accuracy of classifying the validation data was checked by Fisher’s Linear Discriminant Analysis (LDA) (Table Q) and linear kernel-Support Vector Machine (SVM) (Table R). For the keratocyte and HL-60 -type morphology, both LDA (Table Q) and SVM (Table R) yielded good classification of the hand-crafted features comparable to that by the deep learning-based features (Table B). In contrast, for the *Dictyostelium* (agg) data, the deep learning-based features gave more than 40% points more accurate classification compared to the hand-crafted features using LDA (Table Q) or SVM (Table R).

**4. Related mathematical models**

Summarized in Table S are abilities of related mathematical models to describe the following shape dynamics: 1) A constant canoe-like shape with high directional persistence, 2) Elongated form with directional persistence, 3) Pseudopod-like activity like Y-split. Of notable interest with respect to comparisons with our model, Neilson *et al.*, 2011 [38] reported a model where membrane protrusions are described by excitable dynamics based on the Meihardt’s reaction diffusion equation. While the model captures pseudopod-like activities, however a uni-directional migration with a constant canoe-like shape cannot be described by this model since the protrusion is always transient due to the excitable signaling kinetics. Shi *et al.* [39] assumes an excitable dynamics with an additional slow variable that serves as memory. The model is able to capture some degree of directional persistence to the pseudopod-driven dynamics, however the resulting polarized form is inherently oscillatory and does not maintain a constant canoe-like shape with high directional persistence (Figs 1-3 in [39]). The model by Bhattacharya *et al.* [40] depicts an excitable system that supports a travelling wave along cell periphery that stalls and forms a standing wave. The model describes amoeboid-type protrusion dynamics and captures well pseudopod-like morphologies however lacks persistent directional motion due to absence of mechanism that maintain a polarized reading edge (Movie S4 and S5 in [40]). The model by Miao *et al.* [14] based on excitable dynamics captures oscillatory, amoeboid-like and fan-shape-like morphologies. The ’fan-shaped’ morphology in their simulation (Video 13 in [14]) is also highly oscillatory and its morphology feature mapped far from their reference data of a cell modulated with PIP2-phosphatase (Fig. 2E and Video 4 in [14]) (S10B Fig). While strongly oscillatory shapes can also be seen in the two-variable limit (i.e. excitable) of the present model [70], shape dynamics that map closer to the reference experimental data (Video 13 in [14]) can be obtained in the vicinity of the S4C Fig simulation (i.e. weak polarity regime) by shifting the noise term (*θ* and *σ*) and the elongation speed *a* (S10A and S10B Figs). The variable (*V*) (S10C Fig; arrows), the boundary curvature wave and the protrusion patterns (S10D Fig; right panel) also represented well the fluctuating V-shaped bifurcating fronts which is enriched in F-actin binding protein *LimE* (Fig. 2E in [14], S10D Fig; left panel)*.* These features were obscure if not absent in the earlier model simulation (S10D Fig; middle panel).

The model by Moreno *et al.* [53] (hereafter referred to as the ‘stochastic bistable model’) on the other hand is based on bistability dynamics with a slowly relaxing noise variable. The noise variable serves to introduce memory to the system and thus supports elongated morphologies with and without directional persistence and thus comes close to our present model in capturing the morphology dynamics. For detail comparison, we revisited the stochastic bistable model [53] by simulating total of 105 parameter sets that combine 5 conditions for *σ*, 3 conditions for *k*a, and 7 conditions for *C*_0_ in [53] (Table T). S11A Fig plots the average DNN features. As for the feature distances between real cell data and the corresponding rank1 simulations, Score-H, and Score-K were somewhat better in the stochastic bistable model compared to our simulations whereas our model was vastly superior in Score-D (Table U). Note that the noise parameter was essential in the stochastic bistable model and was allowed to change in their study. The noise value in our model grid-search was fixed to accommodate *Dictyostelium*-like morphology at the cost of introducing more perturbation in the high Score-H and high Score-K regions. Time-averaged morphology features of the stochastic bistable model showed a distribution similar to that of our model with less coverage of high PC2 regions (S11A Fig). A closer inspection of snapshot images for the top Score-D(agg) simulations in the stochastic bistable model revealed that they were confined to a much narrower region in the morphology space (S11B Fig) meaning that the low PC1 components that signify complex protrusive structures were missing. This was expected, as the model, with the exception of special noise term, was essentially a polarity model and thus corresponded to our 1-variable model limit (Figs S4E-S4H). Consistent with this morphology feature, the proportion of de novo formation in the stochastic bistable model was almost non-existent (S11D Fig). The top Score-H simulations in the stochastic bistable model show slightly higher PC2 components (S11C Fig) compared to our model (S11C Fig), and the proportion of de novo formation was also markedly small (S11D Fig).

**Table A. Data samples used for shape feature extraction (total snapshot images).**

| **Dataset** | ***Dictyostelium***  **(aggregation-stage)** | **HL-60** | **keratocyte** |
| --- | --- | --- | --- |
| Training | 2430 (1215) | 2286 (1143) | 2393 (731) |
| Validation | 296 (234) | 296 (296) | 296 (211) |

Sample # (Sample # before data augmentation)

**Table B. Classification accuracy of snapshot images.**

| **Validation dataset** | **Predicted class** | | |
| --- | --- | --- | --- |
|  | ***Dictyostelium***  **(aggregation-stage)** | **HL-60** | **keratocyte** |
| ***Dictyostelium***  **(aggregation-stage)** | 94.6% | 5.4% | 0.0% |
| **HL-60** | 4.1% | 96.0% | 0.0% |
| **keratocyte** | 11.2% | 1.0% | 87.8% |

**Table C. Parameters for the phase-field dynamics (Eq. 1).**

| **Parameter** | **Description** | **Value(s)** | **Misc.** |
| --- | --- | --- | --- |
| *τ* | Viscous friction coefficient | 0.83× *τ'* [pN/μm^2^] | Fixed |
| *η* | Surface tension | 1.0 [pN] | Fixed |
| *ε* | Spatial scale of the phase boundary | 1.0 [μm] | Fixed |
| *M* | Area conservation constraint | 0.5 [pN/μm^3^] | Fixed |
| *A*_0_ | Cell area | 78.83 [μm^2^] | Fixed |
| *a*_W_ | Protrusion force | 0.8, 1.6, 2.4, 3.2, 4.0 [pN/μm (conc.)^-1^] |  |

**Table D: Parameters for the kinetic equations (Eqs. 2a-c).**

| **Parameter** | **Description** | **Value(s)** | **Misc.** |
| --- | --- | --- | --- |
| *α* | Relative strength of the feedback from Vand Wto reaction V→U | 2.0 | Fixed (for 1-variable regime,  also *α* = 6, 10) |
| *β* | Relative strength of the feedback from Uto reaction U→V | 3.5 | Fixed |
| *s* | Supply rate of U | 1.0 | Fixed |
| *γ* | Decay rate of U | 0.1, 0.3, 0.5, 0.7 |  |
| *μ* | Decay rate of V | 0.1, 0.3, 0.5, 0.7, 0.9 |  |
| *D*_U_ | Diffusion coefficient of U | 0.05 | Fixed |
| *D*_V_ | Diffusion coefficient of V | 0.2 | Fixed |
| *χ*_U_ | Absorption rate of *U* at the edge | 0, 10, 30, 50, 70 |  |
| *ζ* | Activation rate of Wby V | 200 | Fixed |
| *k*_W1_ | Decay rate of W | 10, 20, 50, 90, 95, 100, 105, 110, 220 |  |
| *ρ* | Relative rate of W auto-regulation | 4.55, 4.76, 5, 5.26, 5.56 |  |
| *D*_W_ | Diffusion coefficient of W | 1.2, 3.0 |  |
| *W*_tot_ | Total amount of W and W* | 50, 60, 70, 80, 90, 100, 110, 120 |  |
| *K_K_* | Effective Michaelis-Menten constant | 3.5 | Fixed |
| *K_P_* | Effective Michaelis-Menten constant | 3.2 | Fixed |
| *τ'* | Time scaling factor | 10 |  |

**Table E. Parameters for the 2-variable (*V*-*U*) equations.**

| (*χ*_U_, *μ*, *γ*, *a*_V_, *α*, σ) | | |
| --- | --- | --- |
| 0, 0.1, 0.1, 24, 2, 0.075  0, 0.5, 0.1, 24, 2, 0.075  0, 0.5, 0.3, 24, 2, 0.075  0, 0.5, 0.5, 24, 2, 0.075  0, 0.1, 0.1, 24, 10, 0.075  0, 0.5, 0.1, 24, 10, 0.075  0, 0.5, 0.3, 24, 10, 0.075  0, 0.5, 0.5, 24, 10, 0.075  50, 0.1, 0.1, 24, 2, 0.075  50, 0.5, 0.1, 24, 2, 0.075  50, 0.5, 0.3, 24, 2, 0.075  50, 0.5, 0.5, 24, 2, 0.075  50, 0.1, 0.1, 24, 10, 0.075  50, 0.5, 0.1, 24, 10, 0.075  50, 0.5, 0.3, 24, 10, 0.075  50, 0.5, 0.5, 24, 10, 0.075  0, 0.5, 0.5, 24, 6, 0.075  0, 0.1, 0.1, 24, 6, 0.075  0, 0.5, 0.1, 24, 6, 0.075  0, 0.5, 0.3, 24, 6, 0.075  50, 0.1, 0.1, 24, 6, 0.075  50, 0.5, 0.1, 24, 6, 0.075  50, 0.5, 0.3, 24, 6, 0.075  50, 0.5, 0.5, 24, 6, 0.075 | 0, 0.3, 0.1, 24, 2, 0.075  0, 0.7, 0.1, 24, 2, 0.075  0, 0.9, 0.1, 24, 2, 0.075  0, 0.5, 0.7, 24, 2, 0.075  0, 0.3, 0.1, 24, 10, 0.075  0, 0.7, 0.1, 24, 10, 0.075  0, 0.9, 0.1, 24, 10, 0.075  0, 0.5, 0.7, 24, 10, 0.075  50, 0.3, 0.1, 24, 2, 0.075  50, 0.7, 0.1, 24, 2, 0.075  50, 0.9, 0.1, 24, 2, 0.075  50, 0.5, 0.7, 24, 2, 0.075  50, 0.3, 0.1, 24, 10, 0.075  50, 0.7, 0.1, 24, 10, 0.075  50, 0.9, 0.1, 24, 10, 0.075  50, 0.5, 0.7, 24, 10, 0.075  0, 0.3, 0.1, 24, 6, 0.075  0, 0.7, 0.1, 24, 6, 0.075  0, 0.9, 0.1, 24, 6, 0.075  0, 0.5, 0.7, 24, 6, 0.075  50, 0.3, 0.1, 24, 6, 0.075  50, 0.7, 0.1, 24, 6, 0.075  50, 0.9, 0.1, 24, 6, 0.075  50, 0.5, 0.7, 24, 6, 0.075 | 0, 0.1, 0.1, 8, 2, 0.3  0, 0.5, 0.1, 8, 2, 0.3  0, 0.5, 0.3, 8, 2, 0.3  0, 0.5, 0.5, 8, 2, 0.3  0, 0.3, 0.1, 8, 2, 0.3  0, 0.7, 0.1, 8, 2, 0.3  0, 0.9, 0.1, 8, 2, 0.3  0, 0.5, 0.7, 8, 2, 0.3  0, 0.1, 0.1, 8,10, 0.3  0, 0.5, 0.1, 8, 10, 0.3  0, 0.5, 0.3, 8, 10, 0.3  0, 0.5, 0.5, 8, 10, 0.3  0, 0.3, 0.1, 8, 10, 0.3  0, 0.7, 0.1, 8, 10, 0.3  0, 0.9, 0.1, 8, 10, 0.3  0, 0.5, 0.7, 8, 10, 0.3  50, 0.1, 0.1, 8, 2, 0.3  50, 0.5, 0.1, 8, 2, 0.3  50, 0.5, 0.3, 8, 2, 0.3  50, 0.5, 0.5, 8, 2, 0.3  50, 0.3, 0.1, 8, 2, 0.3  50, 0.7, 0.1, 8, 2, 0.3  50, 0.9, 0.1, 8, 2, 0.3  50, 0.5, 0.7, 8, 2, 0.3 |

**Table F. Parameters for the 1-variable (*W*) equation.**

| (*k*_W1_, *ρ*, *a*_W_, *D*_W_, *W*_tot_, *σ*) | | |
| --- | --- | --- |
| 10, 5, 2.4, 3, 80, 0.15  20, 5, 2.4, 3, 80, 0.15  50, 5, 2.4, 3, 80, 0.15  100, 5, 2.4, 3, 80, 0.15  90, 5.5556, 2.4, 3, 80, 0.15  95, 5.2632, 2.4, 3, 80, 0.15  105, 4.7619, 2.4, 3, 80, 0.15  110, 4.5455, 2.4, 3, 80, 0.15  110, 4.5455, 0.8, 3, 80, 0.15  110, 4.5455, 1.6, 3, 80, 0.15  110, 4.5455, 3.2, 3, 80, 0.15  110, 4.5455, 4, 3, 80, 0.15  110, 4.5455, 2.4, 3, 60, 0.15  110, 4.5455, 2.4, 3, 70, 0.15  110, 4.5455, 2.4, 3, 90, 0.15  110, 4.5455, 2.4, 3, 100, 0.15  90, 5.5556, 2.4, 3, 80, 0.5  95, 5.2632, 2.4, 3, 80, 0.5  105, 4.7619, 2.4, 3, 80, 0.5 | 110, 4.5455, 2.4, 3, 80, 0.5  110, 4.5455, 0.8, 3, 80, 0.5  110, 4.5455, 1.6, 3,80, 0.5  110, 4.5455, 3.2, 3,80, 0.5  110, 4.5455, 4,3, 80, 0.5  110, 4.5455, 2.4, 3, 60, 0.5  110, 4.5455, 2.4, 3, 70, 0.5  110, 4.5455, 2.4, 3, 90, 0.5  110, 4.5455, 2.4, 3, 100, 0.5  110, 4.5455, 2.4, 3, 110, 0.5  110, 4.5455, 2.4, 3, 110, 0.15  110, 4.5455, 2.4, 3, 120, 0.5  110, 4.5455, 2.4, 3, 120, 0.15  80, 6.25, 0.8, 3, 80, 0.15  80, 6.25, 1.6, 3, 80, 0.15  80, 6.25, 3.2, 3, 80, 0.15  80, 6.25, 4, 3, 80, 0.15  90, 5.5556, 1.6, 3, 80, 0.15  100, 5, 1.6, 3, 80, 0.15 | 75, 6.6667, 1.6, 3, 80, 0.15  85, 5.8824, 1.6, 3, 80, 0.15  90, 5.5556, 1.6, 3, 100, 0.15  90, 5.5556, 0.8, 3, 100, 0.15  90, 7.6923, 1.6, 3, 80, 0.15  45, 5.5556, 1.6, 3, 100, 0.15  90, 5.5556, 0.4, 3, 100, 0.15  45, 5.5556, 0.8, 3, 100, 0.15  70, 7.1429, 0.4, 3, 80, 0.15  35, 7.1429, 0.8, 3, 80, 0.15  70, 7.1429, 0.4, 3, 100, 0.15  80, 6.2500, 0.4, 3, 100, 0.15  35, 7.1429, 0.8, 3, 100, 0.15  40, 6.2500, 0.8, 3, 100, 0.15  80, 6.2500, 0.4, 3, 90, 0.15  40, 6.2500, 0.8, 3, 90, 0.15  80, 6.2500, 0.8, 3, 90, 0.15  40, 6.2500, 1.6, 3, 90, 0.15 |

**Table G. Model parameters (228 sets).**

| (*χ*_U_, *k*_W1_, *μ*, *ρ*, *γ*, *a*_W_, *D*_W_, *W*_tot_) | | |
| --- | --- | --- |
| 0,110,0.5,4.5455,0.1,0.8,1.2,80  0,110,0.5,4.5455,0.1,1.6,1.2,80  0,110,0.5,4.5455,0.1,2.4,1.2,80  0,110,0.5,4.5455,0.1,3.2,1.2,80  0,110,0.5,4.5455,0.1,4,1.2,80  0,110,0.5,4.5455,0.3,0.8,1.2,80  0,110,0.5,4.5455,0.3,1.6,1.2,80  0,110,0.5,4.5455,0.3,2.4,1.2,80  0,110,0.5,4.5455,0.3,3.2,1.2,80  0,110,0.5,4.5455,0.3,4,1.2,80  0,110,0.5,4.5455,0.5,0.8,1.2,80  0,110,0.5,4.5455,0.5,1.6,1.2,80  0,110,0.5,4.5455,0.5,2.4,1.2,80  0,110,0.5,4.5455,0.5,3.2,1.2,80  0,110,0.5,4.5455,0.5,4,1.2,80  0,110,0.5,4.5455,0.7,0.8,1.2,80  0,110,0.5,4.5455,0.7,1.6,1.2,80  0,110,0.5,4.5455,0.7,2.4,1.2,80  0,110,0.5,4.5455,0.7,3.2,1.2,80  0,110,0.5,4.5455,0.7,4,1.2,80  0,110,0.5,4.5455,0.1,2.4,1.2,50  0,110,0.5,4.5455,0.1,2.4,1.8,50  0,110,0.5,4.5455,0.1,2.4,2.4,50  0,110,0.5,4.5455,0.1,2.4,3,50  0,110,0.5,4.5455,0.1,2.4,1.2,60  0,110,0.5,4.5455,0.1,2.4,1.8,60  0,110,0.5,4.5455,0.1,2.4,2.4,60  0,110,0.5,4.5455,0.1,2.4,3,60  0,110,0.5,4.5455,0.1,2.4,1.2,70  0,110,0.5,4.5455,0.1,2.4,1.8,70  0,110,0.5,4.5455,0.1,2.4,2.4,70  0,110,0.5,4.5455,0.1,2.4,3,70  0,110,0.5,4.5455,0.1,2.4,1.2,80  0,110,0.5,4.5455,0.1,2.4,1.8,80  0,110,0.5,4.5455,0.1,2.4,2.4,80  0,110,0.5,4.5455,0.1,2.4,3,80  0,110,0.5,4.5455,0.1,2.4,1.2,90  0,110,0.5,4.5455,0.1,2.4,1.8,90  0,110,0.5,4.5455,0.1,2.4,2.4,90  0,110,0.5,4.5455,0.1,2.4,3,90  0,110,0.5,4.5455,0.1,2.4,1.2,100  0,110,0.5,4.5455,0.1,2.4,1.8,100  0,110,0.5,4.5455,0.1,2.4,2.4,100  0,110,0.5,4.5455,0.1,2.4,3,100  0,110,0.5,4.5455,0.1,2.4,1.2,110  0,110,0.5,4.5455,0.1,2.4,1.8,110  0,110,0.5,4.5455,0.1,2.4,2.4,110  0,110,0.5,4.5455,0.1,2.4,3,110  50,110,0.5,4.5455,0.1,0.8,1.2,80  50,110,0.5,4.5455,0.1,1.6,1.2,80  50,110,0.5,4.5455,0.1,2.4,1.2,80  50,110,0.5,4.5455,0.1,3.2,1.2,80  50,110,0.5,4.5455,0.1,4,1.2,80  50,110,0.5,4.5455,0.3,0.8,1.2,80  50,110,0.5,4.5455,0.3,1.6,1.2,80  50,110,0.5,4.5455,0.3,2.4,1.2,80  50,110,0.5,4.5455,0.3,3.2,1.2,80  50,110,0.5,4.5455,0.3,4,1.2,80  50,110,0.5,4.5455,0.5,0.8,1.2,80  50,100,0.3,5,0.1,2.4,3,80  50,100,0.5,5,0.1,2.4,3,80  50,100,0.7,5,0.1,2.4,3,80  50,100,0.9,5,0.1,2.4,3,80  50,200,0.1,5,0.1,2.4,3,80  50,200,0.3,5,0.1,2.4,3,80  50,200,0.5,5,0.1,2.4,3,80  50,200,0.7,5,0.1,2.4,3,80  50,200,0.9,5,0.1,2.4,3,80  50,90,0.5,5.5556,0.1,2.4,3,80  50,95,0.5,5.2632,0.1,2.4,3,80  50,100,0.5,5,0.1,2.4,3,80  50,105,0.5,4.7619,0.1,2.4,3,80  50,110,0.5,4.5455,0.1,2.4,3,80  50,90,0.5,5.5556,0.3,2.4,3,80  50,95,0.5,5.2632,0.3,2.4,3,80  50,100,0.5,5,0.3,2.4,3,80  50,105,0.5,4.7619,0.3,2.4,3,80  50,110,0.5,4.5455,0.3,2.4,3,80  50,90,0.5,5.5556,0.5,2.4,3,80  50,95,0.5,5.2632,0.5,2.4,3,80  50,100,0.5,5,0.5,2.4,3,80  50,105,0.5,4.7619,0.5,2.4,3,80  50,110,0.5,4.5455,0.5,2.4,3,80  50,90,0.5,5.5556,0.7,2.4,3,80  50,95,0.5,5.2632,0.7,2.4,3,80  50,100,0.5,5,0.7,2.4,3,80  50,105,0.5,4.7619,0.7,2.4,3,80  50,110,0.5,4.5455,0.7,2.4,3,80  50,90,0.5,5.5556,0.1,2.4,3,80  50,95,0.5,5.2632,0.1,2.4,3,80  50,100,0.5,5,0.1,2.4,3,80  50,105,0.5,4.7619,0.1,2.4,3,80  50,110,0.5,4.5455,0.1,2.4,3,80  50,90,0.5,5.5556,0.3,2.4,3,80  50,95,0.5,5.2632,0.3,2.4,3,80  50,100,0.5,5,0.3,2.4,3,80  50,105,0.5,4.7619,0.3,2.4,3,80  50,110,0.5,4.5455,0.3,2.4,3,80  50,90,0.5,5.5556,0.5,2.4,3,80  50,95,0.5,5.2632,0.5,2.4,3,80  50,100,0.5,5,0.5,2.4,3,80  50,105,0.5,4.7619,0.5,2.4,3,80  50,110,0.5,4.5455,0.5,2.4,3,80  50,90,0.5,5.5556,0.7,2.4,3,80  50,95,0.5,5.2632,0.7,2.4,3,80  50,100,0.5,5,0.7,2.4,3,80  50,105,0.5,4.7619,0.7,2.4,3,80  50,110,0.5,4.5455,0.7,2.4,3,80 | 50,110,0.5,4.5455,0.5,1.6,1.2,80  50,110,0.5,4.5455,0.5,2.4,1.2,80  50,110,0.5,4.5455,0.5,3.2,1.2,80  50,110,0.5,4.5455,0.5,4,1.2,80  50,110,0.5,4.5455,0.7,0.8,1.2,80  50,110,0.5,4.5455,0.7,1.6,1.2,80  50,110,0.5,4.5455,0.7,2.4,1.2,80  50,110,0.5,4.5455,0.7,3.2,1.2,80  50,110,0.5,4.5455,0.7,4,1.2,80  0,110,0.5,4.5455,0.1,2.4,1.2,50  0,110,0.5,4.5455,0.1,2.4,1.8,50  0,110,0.5,4.5455,0.1,2.4,2.4,50  50,110,0.5,4.5455,0.1,2.4,3,50  50,110,0.5,4.5455,0.1,2.4,1.2,60  0,110,0.5,4.5455,0.1,2.4,1.8,60  0,110,0.5,4.5455,0.1,2.4,2.4,60  50,110,0.5,4.5455,0.1,2.4,3,60  50,110,0.5,4.5455,0.1,2.4,1.2,70  0,110,0.5,4.5455,0.1,2.4,1.8,70  0,110,0.5,4.5455,0.1,2.4,2.4,70  50,110,0.5,4.5455,0.1,2.4,3,70  0,110,0.5,4.5455,0.1,2.4,1.2,80  0,110,0.5,4.5455,0.1,2.4,1.8,80  0,110,0.5,4.5455,0.1,2.4,2.4,80  50,110,0.5,4.5455,0.1,2.4,3,80  50,110,0.5,4.5455,0.1,2.4,1.2,90  0,110,0.5,4.5455,0.1,2.4,1.8,90  0,110,0.5,4.5455,0.1,2.4,2.4,90  50,110,0.5,4.5455,0.1,2.4,3,90  50,110,0.5,4.5455,0.1,2.4,1.2,100  0,110,0.5,4.5455,0.1,2.4,1.8,100  0,110,0.5,4.5455,0.1,2.4,2.4,100  50,110,0.5,4.5455,0.1,2.4,3,100  50,110,0.5,4.5455,0.1,2.4,1.2,110  0,110,0.5,4.5455,0.1,2.4,1.8,110  0,110,0.5,4.5455,0.1,2.4,2.4,110  50,110,0.5,4.5455,0.1,2.4,3,110  0,10,0.1,5,0.1,2.4,3,80  0,10,0.3,5,0.1,2.4,3,80  0,10,0.5,5,0.1,2.4,3,80  0,10,0.7,5,0.1,2.4,3,80  0,10,0.9,5,0.1,2.4,3,80  0,20,0.1,5,0.1,2.4,3,80  0,20,0.3,5,0.1,2.4,3,80  0,20,0.5,5,0.1,2.4,3,80  0,20,0.7,5,0.1,2.4,3,80  0,20,0.9,5,0.1,2.4,3,80  0,50,0.1,5,0.1,2.4,3,80  0,50,0.3,5,0.1,2.4,3,80  0,50,0.5,5,0.1,2.4,3,80  0,50,0.7,5,0.1,2.4,3,80  0,50,0.9,5,0.1,2.4,3,80  0,100,0.1,5,0.1,2.4,3,80  0,100,0.3,5,0.1,2.4,3,80  0,100,0.5,5,0.1,2.4,3,80  0,100,0.7,5,0.1,2.4,3,80  0,100,0.9,5,0.1,2.4,3,80  0,200,0.1,5,0.1,2.4,3,80  0,200,0.3,5,0.1,2.4,3,80 | 0,200,0.5,5,0.1,2.4,3,80  0,200,0.7,5,0.1,2.4,3,80  0,200,0.9,5,0.1,2.4,3,80  0,90,0.5,5.5556,0.1,2.4,3,80  0,95,0.5,5.2632,0.1,2.4,3,80  0,100,0.5,5,0.1,2.4,3,80  0,105,0.5,4.7619,0.1,2.4,3,80  0,110,0.5,4.5455,0.1,2.4,3,80  0,90,0.5,5.5556,0.3,2.4,3,80  0,95,0.5,5.2632,0.3,2.4,3,80  0,100,0.5,5,0.3,2.4,3,80  0,105,0.5,4.7619,0.3,2.4,3,80  0,110,0.5,4.5455,0.3,2.4,3,80  0,90,0.5,5.5556,0.5,2.4,3,80  0,95,0.5,5.2632,0.5,2.4,3,80  0,100,0.5,5,0.5,2.4,3,80  0,105,0.5,4.7619,0.5,2.4,3,80  0,110,0.5,4.5455,0.5,2.4,3,80  0,90,0.5,5.5556,0.7,2.4,3,80  0,95,0.5,5.2632,0.7,2.4,3,80  0,100,0.5,5,0.7,2.4,3,80  0,105,0.5,4.7619,0.7,2.4,3,80  0,110,0.5,4.5455,0.7,2.4,3,80  0,90,0.5,5.5556,0.1,2.4,3,80  0,95,0.5,5.2632,0.1,2.4,3,80  0,100,0.5,5,0.1,2.4,3,80  0,105,0.5,4.7619,0.1,2.4,3,80  0,110,0.5,4.5455,0.1,2.4,3,80  0,90,0.5,5.5556,0.3,2.4,3,80  0,95,0.5,5.2632,0.3,2.4,3,80  0,100,0.5,5,0.3,2.4,3,80  0,105,0.5,4.7619,0.3,2.4,3,80  0,110,0.5,4.5455,0.3,2.4,3,80  0,90,0.5,5.5556,0.5,2.4,3,80  0,95,0.5,5.2632,0.5,2.4,3,80  0,100,0.5,5,0.5,2.4,3,80  0,105,0.5,4.7619,0.5,2.4,3,80  0,110,0.5,4.5455,0.5,2.4,3,80  0,90,0.5,5.5556,0.7,2.4,3,80  0,95,0.5,5.2632,0.7,2.4,3,80  0,100,0.5,5,0.7,2.4,3,80  0,105,0.5,4.7619,0.7,2.4,3,80  0,110,0.5,4.5455,0.7,2.4,3,80  50,10,0.1,5,0.1,2.4,3,80  50,10,0.3,5,0.1,2.4,3,80  50,10,0.5,5,0.1,2.4,3,80  50,10,0.7,5,0.1,2.4,3,80  50,10,0.9,5,0.1,2.4,3,80  50,20,0.1,5,0.1,2.4,3,80  50,20,0.3,5,0.1,2.4,3,80  50,20,0.5,5,0.1,2.4,3,80  50,20,0.7,5,0.1,2.4,3,80  50,20,0.9,5,0.1,2.4,3,80  50,50,0.1,5,0.1,2.4,3,80  50,50,0.3,5,0.1,2.4,3,80  50,50,0.5,5,0.1,2.4,3,80  50,50,0.5,5,0.1,2.4,3,80  50,50,0.9,5,0.1,2.4,3,80  50,100,0.1,5,0.1,2.4,3,80 |

**Table H. Top ranking parameters for Score-D, Score-H and Score-K.**

| Reference  morphology | \|\|***F***\|\|_2_ | CNN layer | score rank | *χ_U_* *k*_W1_ * * *ρ* * γ* *a*_W_ *D*_W_ *W*_tot_ |
| --- | --- | --- | --- | --- |
| Score-D  (agg) | 4954  11637  12855  13917  15273  17338  18571  18933  19327  20461  20570 | Final layer | 1  2  3  4  5  6  7  8  9  10  11 | 0 110.0    0.5 4.5455 0.3 4.0    1.2   80.0  0 110.0    0.5 4.5455 0.7 4.0    1.2   80.0  50.0  110.0 0.5 4.5455    0.5   4.0    1.2   80.0  0 110.0 0.5    4.5455    0.5    4.0    1.2   80.0  50.0  110.0 0.5    4.5455    0.7    4.0    1.2   80.0  0 110.0    0.5    4.5455    0.5    3.2    1.2   80.0  50.0  110.0 0.5    4.5455    0.3    4.0    1.2   80.0  50.0  110.0 0.5    4.5455   0.1    4.0    1.2   80.0  0 110.0    0.5    4.5455    0.1    4.0    1.2   80.0  50.0  110.0    0.5    4.5455    0.5    3.2    1.2   80.0  0 110.0    0.5 4.5455 0.3 2.4 3.0   80.0 |
| Score-H | 2561  3350  4447  5913  6320  6420  6564  7235  7804  8075  8405 | Final layer | 1  2  3  4  5  6  7  8  9  10  11 | 50.0  100.0 0.5 5.0 0.1    1.6    3.0   80.0  0 90.0    0.5    5.5556    0.3    2.4    3.0   80.0  0 90.0    0.5    5.5556    0.1    2.4    3.0   80.0  0 95.0 0.5 5.2632    0.1    2.4    3.0   80.0  0 100.0    0.1    5.0    0.1    2.4    3.0   80.0  50.0  100.0    0.7    5.0 0.1    2.4    3.0   80.0  0 50.0    0.3    5.0 0.1    2.4    3.0   80.0  50.0  110.0    0.5    4.5455    0.1    1.6    1.2   80.0  0 50.0    0.7    5.0    0.1    2.4    3.0   80.0  0 100.0    0.5    5.0 0.1    1.6    3.0   80.0   50.0 110.0    0.5    4.5455    0.1    0.8    1.2   80.0 |
| Score-K | 18021  18189  26460  40680  41350  44566  50622  55830  56589  59338  60322 | Final layer | 1  2  3  4  5  6  7  8  9  10  11 | 50.0   20.0    0.1    5.0    0.1    2.4    3.0   80.0  50.0  　50.0    0.1    5.0    0.1    2.4    3.0   80.0  50.0  　10.0    0.1    5.0    0.1    2.4    3.0   80.0  50.0  110.0    0.5    4.5455    0.1    2.4    1.2  100.0  50.0  100.0    0.5    5.0    0.1    1.6    3.0  100.0  50.0  100.0    0.1    5.0    0.1    2.4    3.0   80.0  50.0  110.0    0.5    4.5455    0.1    2.4    1.2   90.0  50.0   20.0 0.3 5.0    0.1    2.4    3.0   80.0  0 110.0    0.5    4.5455    0.1    2.4    1.8  100.0  0 110.0    0.5    4.5455    0.1    2.4    1.2  100.0  50.0  110.0    0.5    4.5455    0.1    2.4    1.8  100.0 |

**Table I. Classification accuracy of high Score-D (agg) simulations.**

| **score rank** | **Predicted class** | | |
| --- | --- | --- | --- |
|  | ***Dictyostelium***  **(aggregation-stage)** | **HL-60** | **keratocyte** |
| Score-D (agg) rank1 | 97.0% | 2.9% | 0.1% |
| Score-D (agg) rank2 | 94.0% | 5.6% | 0.4% |
| Score-D (agg) rank3 | 91.1% | 8.9% | 0.0% |
| Score-D (agg) rank4 | 92.1% | 7.6% | 0.2% |
| Score-D (agg) rank5 | 92.1% | 7.8% | 0.1% |
| Score-D (agg) rank6 | 91.1% | 8.7% | 0.3% |
| Score-D (agg) rank7 | 91.2% | 8.4% | 0.4% |
| Score-D (agg) rank8 | 90.5% | 9.5% | 0.0% |
| Score-D (agg) rank9 | 93.9% | 6.1% | 0.0% |
| Score-D (agg) rank10 | 86.5% | 13.2% | 0.3% |

**Table J. Classification accuracy of high Score-H simulations.**

| **score rank** | **Predicted class** | | |
| --- | --- | --- | --- |
|  | ***Dictyostelium***  **(aggregation-stage)** | **HL-60** | **keratocyte** |
| Score-H rank1 | 5.1% | 94.7% | 0.1% |
| Score-H rank2 | 7.1% | 92.9% | 0.0% |
| Score-H rank3 | 4.3% | 95.7% | 0.0% |
| Score-H rank4 | 16.8% | 83.2% | 0.0% |
| Score-H rank5 | 11.1% | 88.9% | 0.0% |
| Score-H rank6 | 9.5% | 90.5% | 0.0% |
| Score-H rank7 | 8.0% | 92.0% | 0.0% |
| Score-H rank8 | 16.6% | 83.1% | 0.3% |
| Score-H rank9 | 14.9% | 85.1% | 0.0% |
| Score-H rank10 | 6.3% | 93.7% | 0.0% |

**Table K. Classification accuracy of high Score-K simulations.**

| **score rank** | **Predicted class** | | |
| --- | --- | --- | --- |
|  | ***Dictyostelium***  **(aggregation-stage)** | **HL-60** | **keratocyte** |
| Score-K rank1 | 27.5% | 7.5% | 64.9% |
| Score-K rank2 | 20.9% | 13.7% | 65.4% |
| Score-K rank3 | 36.6% | 8.2% | 55.2% |
| Score-K rank4 | 50.1% | 9.8% | 40.1% |
| Score-K rank5 | 45.2% | 15.6% | 39.2% |
| Score-K rank6 | 29.8% | 37.6% | 32.6% |
| Score-K rank7 | 60.9% | 8.2% | 31.0% |
| Score-K rank8 | 33.1% | 52.6% | 14.3% |
| Score-K rank9 | 40.5% | 36.0% | 23.5% |
| Score-K rank10 | 42.1% | 40.8% | 17.1% |

**Table L. Top ranking parameters for Score-D (veg) and Score-H (nocodazole) and Score-D (*racE*-) .**

| Reference  morphology | \|\|***F***\|\|_2_ | CNN layer | score rank | *χ*_U_ *k*_W1_ *μ* *ρ* * γ* *a*_W_ *D*_W_ *W*_tot_ |
| --- | --- | --- | --- | --- |
| Score-D  (veg) | 5671  5798  6238  6382  6434  6742  7058  7239  7277  7495  7509 | Final layer | 1  2  3  4  5  6  7  8  9  10  11 | 0 50.0 0.9 5.0 0.1 2.4 3.0   80.0  50.0  110.0 0.5 4.5455 0.1 2.4 3.0   80.0  50.0  100.0 0.9 5.0 0.1 2.4 3.0   80.0  0 200.0 0.3 5.0 0.1 2.4 3.0   80.0  0 100.0    0.7 5.0 0.1 2.4 3.0   80.0  0 100.0 0.9 5.0 0.1 2.4 3.0   80.0  50.0  100.0 0.5 5.0 0.1 2.4 3.0   80.0  50.0  200.0 0.5 5.0 0.1 2.4 3.0   80.0  50.0  200.0 0.3 5.0 0.1 2.4 3.0   80.0  50.0  100.0 0.5 5.0 0.3 2.4 3.0   80.0  50.0  200.0 0.7 5.0 0.1 2.4 3.0   80.0 |
| (agg) to (veg)  comparison |  | Final layer |  | - n/a - n/a ** ** n/a - |
| Score-H  (nocodazole) | 2568  5559  5668  5906  6821  7916  9075  9402  11260  11278  11316 | Final layer | 1  2  3  4  5  6  7  8  9  10  11 | 0 100.0 0.5 5.0 0.1 1.6 3.0   90.0  50.0  10.0 0.3 5.0 0.1 2.4 3.0   80.0  0 100.0 0.5 5.0 0.1 2.4 3.0   90.0  0 110.0 0.5 4.5455 0.1 2.4 3.0  110.0  0 10.0 0.3 5.0 0.1 2.4 3.0   80.0  0 110.0 0.5 4.5455 0.1 2.4 1.8  110.0  50.0  20.0 0.5 5.0 0.1 2.4 3.0   80.0  50.0  100.0 0.5 5.0 0.1 0.8 3.0   90.0  50.0  20.0 0.9 5.0 0.1 2.4 3.0   80.0  0 110.0 0.5 4.5455 0.1 2.4 1.2  110.0  0 100.0    0.5    5.0 0.1    1.6 3.0  100.0 |
| Score-D (*racE*-) | 5664  12327  17115  20419  22708  24674  26676  28313  28548  28812  30237 | Final layer | 1  2  3  4  5  6  7  8  9  10  11 | 50.0  110.0 0.5 4.5455 0.1 2.4 1.2   90.0  50.0  110.0 0.5 4.5455 0.1 2.4 1.2  100.0  50.0  110.0 0.5 4.5455 0.1 2.4 1.8  100.0  50.0  110.0 0.5 4.5455 0.1 2.4 2.4  100.0  50.0  100.0 0.5 5.0 0.1 1.6 3.0  100.0  50.0  110.0 0.5 4.5455 0.1 2.4 3.0  100.0  50.0  10.0 0.1 5.0 0.1 2.4 3.0   80.0  0 110.0 0.5 4.5455 0.1 2.4 1.8  100.0  50.0  100.0 0.5 5.0 0.1 2.4 3.0   90.0  50.0  100.0 0.5 5.0 0.1 2.4 3.0  100.0  0 100.0 0.5 5.0 0.1 2.4 3.0  100.0 |
| (*racE*-) to (agg)  comparison |  | Final layer |  | - n/a - n/a ** ** n/a ** |
| (*racE*-) to (veg)  comparison |  | Final layer |  | - n/a * n/a - - n/a ** |

*… (p < 10%), **… (p < 0.1%), ***… (p < 0.001%), -… not significant

n/a : subject to bias from the choice of parameter search

**Table M. Top ranking parameters for Score-D (veg) and Score-D (agg) based on feature extraction at the intermediate layer .**

| Reference  morphology | \|\|***F***\|\|_2_ | CNN layer | rank | *χ_U_* *k*_W1_ * * *ρ* * γ* *a*_W_ *D*_W_ *W*_tot_ | Table H rank |
| --- | --- | --- | --- | --- | --- |
| Score-D  (veg) | 2701  2744  2880  2988  3083  3190  3375  3378  3382  3393  3433 | Intermediate layer | 1  2  3  4  5  6  7  8  9  10  11 | 0.0 90.0 0.5 5.5556 0.5 2.4 3.0 80.0  50.0 100.0 0.5 5.0 0.3 2.4 3.0 80.0  50.0 105.0 0.5 4.7619 0.1 2.4 3.0 80.0  50.0 100.0 0.5 5.0 0.1 2.4 3.0 80.0  50.0 95.0 0.5 5.2632 0.5 2.4 3.0 80.0  0.0 50.0 0.5 5.0 0.1 2.4 3.0 80.0  50.0 110.0 0.5 4.5455 0.1 2.4 1.2 80.0  50.0 200.0 0.3 5.0 0.1 2.4 3.0 80.0  50.0 100.0 0.5 5.0 0.5 2.4 3.0 80.0  50.0 110.0 0.5 4.5455 0.3 1.6 1.2 80.0  50.0 95.0 0.5 5.2632 0.1 2.4 3.0 80.0 | 21  10  12  7  30  13  20  9  32  39  47 |
| Score-D  (agg) | 2634  2707  2972  3123  3276  3969  4153  4268  4547  4588  4885 | Intermediate layer | 1  2  3  4  5  6  7  8  9  10  11 | 0.0 110.0 0.5 4.5455 0.7 4.0 1.2 80.0  50.0 110.0 0.5 4.5455 0.5 4.0 1.2 80.0  0.0 110.0 0.5 4.5455 0.3 4.0 1.2 80.0  0.0 110.0 0.5 4.5455 0.5 4.0 1.2 80.0  50.0 110.0 0.5 4.5455 0.7 4.0 1.2 80.0  50.0 110.0 0.5 4.5455 0.3 4.0 1.2 80.0  50.0 110.0 0.5 4.5455 0.1 4.0 1.2 80.0  0.0 110.0 0.5 4.5455 0.5 3.2 1.2 80.0  50.0 110.0 0.5 4.5455 0.5 3.2 1.2 80.0  0.0 110.0 0.5 4.5455 0.1 4.0 1.2 80.0  50.0 110.0 0.5 4.5455 0.3 3.2 1.2 80.0 | 2  3  1  4  5  7  8  6  10  9  13 |
| (agg) to (veg)  comparison |  | Intermediate  layer |  | - n/a - n/a * *** n/a - |  |
| Score-D  (*racE*-) | 1719  3180  3929  4523  5599  5613  6068  6177  6778  6785  6789 | Intermediate layer | 1  2  3  4  5  6  7  8  9  10  11 | 50.0 110.0 0.5 4.5455 0.1 2.4 1.2 90.0  50.0 110.0 0.5 4.5455 0.1 2.4 1.2 100.0  50.0 110.0 0.5 4.5455 0.1 2.4 1.8 100.0  50.0 110.0 0.5 4.5455 0.1 2.4 2.4 100.0  50.0 110.0 0.5 4.5455 0.1 2.4 3.0 100.0  50.0 100.0 0.5 5.0 0.1 1.6 3.0 100.0  50.0 10.0 0.1 5.0 0.1 2.4 3.0 80.0  50.0 100.0 0.5 5.0 0.1 2.4 3.0 100.0  50.0 100.0 0.5 5.0 0.1 2.4 3.0 90.0  0.0 100.0 0.5 5.0 0.1 3.2 3.0 100.0  0.0 110.0 0.5 4.5455 0.1 2.4 1.8 100.0 | 1  2  3  4  6  5  7  10  9  14  8 |
| (*racE*-) to (agg)  comparison |  | Intermediate layer |  | - n/a 　 -- n/a ** *** n/a ** |  |
| (*racE*-) to (veg)  comparison |  | Intermediate layer |  | - n/a - n/a * - n/a ** |  |

*… (p < 10%), **… (p < 0.1%), ***… (p < 0.001%), -… not significant

n/a : subject to bias from the choice of parameter search

**Table N. Parameters for representative data in main figures (Figs 2, 3 and 5)**

| **Description** | **Parameters** |
| --- | --- |
| Fig 2B | *χ*_U_ = 50, *γ* = 0.1, *μ* = 0.5, *D*_W_ = 3, *ρ* = 4.55, *k*_W1_ = 110, *W*_tot_ = 80, *a*_W_ = 2.4, |
| Fig 2C | *χ*_U_ = 50, *γ* = 0.1, *μ* = 0.7, *D*_W_ = 3, *ρ* = 4.76, *k*_W1_ = 105, *W*_tot_ = 80, *a*_W_ = 2.4 |
| Fig 2D | *χ*_U_ = 50, *γ* = 0.1, *μ* = 0.5, *D*_W_ = 3, *ρ* = 5.56, *k*_W1_ = 90, *W*_tot_ = [60, 65, 70, 75, 80, 85, 90, 95, 100, 105, 110], *a*_W_ = 2.4 |
| Fig 2E  (black) | *χ*_U_ *=* 50, *γ* = 0.1, *μ* = 0.5, *D*_W_ = 3, *ρ* = 5.56, *k*_W1_ = 90, *W*_tot_ = [60, 65, 70, 75, 80, 85, 90, 95, 100, 105, 110, 115], *a*_W_ = 2.4 |
| (blue) | *χ*_U_ *=* 50, *γ =* 0.1, *μ* = 0.1, *D*_W_ = 3, *ρ =* 5, *k*_W1_ = 100*, W*_tot_ = [50, 55, 60, 65, 70, 75, 80, 85, 90, 95, 100, 105, 110, 115], *a*_W_ = 2.4 |
| (magenta) | *χ*_U_ *=* 50, *γ =* 0.1, *μ* = 0.5, *D*_W_ = 3, *ρ =* 4.55, *k*_W1_ = 110*,* *W*_tot_ = [65, 70, 75, 80, 85, 90, 95, 100, 105, 110, 115, 120, 125, 130], *a*_W_ = 4 |
| Fig 2F | *χ*_U_ = 30, *γ* = 0.1, *μ* = 0.5, *D*_W_ = 3, *ρ* = 4.55, *k*_W1_ = 110, *W*_tot_ = 80, *a*_W_ = [0.8, 1.6, 2.4, 3.2, 4] |
| Fig 2G  (black) | *χ*_U_ *=* 30, *γ* = 0.1, *μ* = 0.5, *D*_W_ = 3, *ρ* = 4.55, *k*_W1_ = 110, *W*_tot_ = 80, *a*_W_ = [0.8, 1.6, 2.4, 3.2, 4, 4.8, 5.6] |
| (blue) | *χ*_U_ *=* 50, *γ* = 0.1, *μ* = 0.1, *D*_W_ = 3, *ρ* = 5, *k*_W1_ = 100, *W*_tot_ = 80, *a*_W_ = [0.8, 1.6, 2.4, 3.2, 4, 4.8, 5.6] |
| Fig 2H | *χ*_U_ = 50, *γ* = 0.1, *μ* = 0.5, *D*_W_ = 3, *ρ* = 4.55, *k*_W1_ = [1.1, 2.2, 11, 55, 110], *W*_tot_ = 80, *a*_W_ = 5.6 |
| Fig 2I  (black) | *χ*_U_ *=* 50, *γ* = 0.1, *μ* = 0.5, *D*_W_ = 3, *ρ* = 4.55, *k*_W1_ = [1.1, 2.2, 11, 22, 55, 110], *W*_tot_ = 80  *a*_W_ = 5.6 |
| (blue) | *χ*_U_ *=* 50, *γ* = 0.1, *μ* = 0.5, *D*_W_ = 3, *ρ* = 4.55, *k*_W1_ = [1.1, 2.2, 11, 22, 55, 110], *W*_tot_ = 90  *a*_W_ = 2.4 |
| (magenta) | *χ*_U_ *=* 50, *γ* = 0.1, *μ* = 0.1, *D*_W_ = 3, *ρ* = 5, *k*_W1_ = [1, 2, 10, 20, 50, 100], *W*_tot_ = 80,  *a*_W_ = 2.4 |
| Fig 2J | *χ*_U_ = 50, *γ* = 0.1, *μ* = [0.1, 0.3, 0.5, 0.7, 0.9], *D*_W_ = 3, *ρ* = 5, *k*_W1_ = 100, *W*_tot_ = 80, *a*_W_ = 2.4 |
| Fig 2K  (black) | *χ*_U_ = 50, *γ* = 0.1, *μ* = [0.1, 0.3, 0.5, 0.7, 0.9], *D*_W_ = 3, *ρ* = 5, *k*_W1_ = 100, *W*_tot_ = 80, *a*_W_ = 2.4 |
| (blue) | *χ*_U_ *=* 50, *γ* = 0.1, *μ* = [0.02, 0.05, 0.1, 0.3, 0.5, 0.7, 0.9], *D*_W_ = 3, *ρ* = 4.55, *k*_W1_ = 110, *W*_tot_ = 80, *a*_W_ = 4.8 |
| Fig 3C-E | *χ*_U_ = 0, *γ* = 0.3, *μ* = 0.5, *D*_W_ = 1.2, *ρ* = 4.55, *k*_W1_ = 110, *W*_tot_ = 80, *a*_W_ = 4 |
| Fig 3F-H | *χ*_U_ = 50, *γ* = 0.1, *μ* = 0.5, *D*_W_ = 3, *ρ* = 5, *k*_W1_ = 100, *W*_tot_ = 80, *a*_W_ = 1.6 |
| Fig 3I-K | *χ*_U_ = 50, *γ* = 0.1, *μ* = 0.1, *D*_W_ = 3, *ρ* = 5, *k*_W1_ = 20, *W*_tot_ = 80, *a*_W_ = 2.4 |
| Fig 5C | *χ*_U_ = 0, *γ* = 0.1, *μ* = 0.9, *D*_W_ = 3, *ρ* = 5, *k*_W1_ = 50, *W*_tot_ = 80, *a*_W_ = 2.4 |
| Fig 5E | *χ*_U_ = 50, *γ* = 0.1, *μ* = 0.5, *D*_W_ = 1.2, *ρ* = 4.55, *k*_W1_ = 110, *W*_tot_ = 90, *a*_W_ = 2.4 |
| Fig 5G | *χ*_U_ = 0, *γ* = 0.1, *μ* = 0.5, *D*_W_ = 3, *ρ* = 5, *k*_W1_ = 100, *W*_tot_ = 90, *a*_W_ = 1.6 |

**Table O. Parameters for representative data in supplementary figures (Figs S4, S5, S6, S8 and S10)**

| **Description** | **Parameters** |
| --- | --- |
| S4B Fig | *α* = 10, *β* = 3.5, *χ*_U_ = 50, *γ* = 0.32, *μ* = 1.0, *θ* = 0.014, *d* = 0.8, *σ* = 7.5, *a*= 35, b=0.09 |
| S4C Fig | *α* = 10, *β* = 3.5, *χ*_U_ = 50, *γ* = 0.32, *μ* = 1.0, *θ* = 14, *d* = 0.8, *σ* = 0.02, *a*= 35, b=0.09 |
| S4F Fig | *D*_W_ = 3, *ρ* = 5.56, *k*_W1_ = 90, *W*_tot_ = 80, *θ* = 2.0, *d* = 5.0, *σ* = 0.75, *a*_W_ = 2.4 |
| S4G Fig | *D*_W_ = 3, *ρ* = 6.25, *k*_W1_ = 80, *W*_tot_ = 90, *θ* = 1.4, *d* = 0.8, *σ* = 0.15, *a*_W_ = 0.8 |
| S5B Fig R1 | *α* = 2, *β* = 3.5, *χ*_U_ = 0, *γ* = 0.1, *μ* = 0.5, *D*_W_ = 1.2, *ρ* = 4.55, *k*_W1_ = 110, *W*_tot_ = 80, *a*_W_ = 2.4 |
| S5B Fig (1) | *α* = 2, *β* = 3.5, *χ*_U_ = 0, *γ* = 0.5, *μ* = 0.5, *D*_W_ = 1.2, *ρ* = 4.55, *k*_W1_ = 110, *W*_tot_ = 80, *a*_W_ = 2.4 |
| S5B Fig (2) | *α* = 2, *β* = 3.5, *χ*_U_ = 0, *γ* = 0.7, *μ* = 0.5, *D*_W_ = 1.2, *ρ* = 4.55, *k*_W1_ = 110, *W*_tot_ = 80, *a*_W_ = 2.4 |
| S5B Fig (3) | *α* = 2, *β* = 3.5, *χ*_U_ = 0, *γ* = 0.5, *μ* = 0.5, *D*_W_ = 1.2, *ρ* = 4.55, *k*_W1_ = 110, *W*_tot_ = 80, *a*_W_ = 0.8 |
| S5B Fig (4) | *α* = 2, *β* = 3.5, *χ*_U_ = 0, *γ* = 0.5, *μ* = 0.5, *D*_W_ = 1.2, *ρ* = 4.55, *k*_W1_ = 110, *W*_tot_ = 80, *a*_W_ = 4 |
| S5C Fig R2’ | *α* = 2, *β* = 3.5, *χ*_U_ = 50, *γ* = 0.1, *μ* = 0.5, *D*_W_ = 3, *ρ* = 5, *k*_W1_ = 100, *W*_tot_ = 80, *a*_W_ = 2.4 |
| S5C Fig (5) | *α* = 2, *β* = 3.5, *χ*_U_ = 50, *γ* = 0.1, *μ* = 0.5, *D*_W_ = 3, *ρ* = 50, *k*_W1_ = 10, *W*_tot_ = 80, *a*_W_ = 2.4 |
| S5C Fig (6) | *α* = 2, *β* = 3.5, *χ*_U_ = 50, *γ* = 0.1, *μ* = 0.5, *D*_W_ = 3, *ρ* = 2.5, *k*_W1_ = 200, *W*_tot_ = 80, *a*_W_ = 2.4 |
| S5C Fig (7) | *α* = 2, *β* = 3.5, *χ*_U_ = 50, *γ* = 0.1, *μ* = 0.1, *D*_W_ = 3, *ρ* = 5, *k*_W1_ = 100, *W*_tot_ = 80, *a*_W_ = 2.4 |
| S5C Fig (8) | *α* = 2, *β* = 3.5, *χ*_U_ = 50, *γ* = 0.1, *μ* = 0.9, *D*_W_ = 3, *ρ* = 5, *k*_W1_ = 100, *W*_tot_ = 80, *a*_W_ = 2.4 |
| S5D Fig | *α* = 2, *β* = 3.5, *χ*_U_ = 0, *γ* = [0.1, 0.3, 0.5, 0.7], *μ* = 0.5, *D*_W_ = 1.2, *ρ* = 4.55, *k*_W1_ = 110, *W*_tot_ = 80, *a*_W_ = [0.8, 1.6, 2.4, 3.2, 4] |
| S5E Fig | *α* = 2, *β* = 3.5, *χ*_U_ = 50, *γ* = 0.1, *μ* = [0.1, 0.3, 0.5, 0.7, 0.9], *D*_W_ = 3, *ρ* = 5, *k*_W1_ = [10, 20, 50, 100, 200], *W*_tot_ = 80, *a*_W_ = 2.4 |
| S6 Fig A, B | *α* = 2, *β* = 3.5, *χ*_U_ = 50, *γ* = 0.1, *μ* = 0.5, *D*_W_ = 3, *ρ* = [4.55, 4.76, 5, 5.26, 5.56], *k*_W1_ = 500, *W*_tot_ = 80, *a*_W_ = 2.4 |
| S6 Fig C, D | *α* = 2, *β* = 3.5, *χ*_U_ = 0, *γ* = [0.1, 0.3, 0.5, 0.7, 0.9], *μ* = 0.5, *D*_W_ = 3, *ρ* = 4.55, *k*_W1_ = 110, *W*_tot_ = 80, *a*_W_ = 2.4 |
| S6 Fig E, F | *α* = 2, *β* = 3.5, *χ*_U_ = 0, *γ* = 0.1, *μ* = 0.5, *D*_W_ = [0.6, 1.2, 1.8, 2.4, 3, 3.6], *ρ* = 4.55, *k*_W1_ = 110, *W*_tot_ = 80, *a*_W_ = 2.4 |
| S6 Fig G, H | *α* = 2, *β* = 3.5, *χ*_U_ = [0, 10, 30, 50], *γ* = 0.1, *μ* = 0.5, *D*_W_ = 3, *ρ* = 4.55, *k*_W1_ = 110, *W*_tot_ = 80, *a*_W_ = 2.4 |
| S8C Fig | *α* = 2, *β* = 3.5, *χ*_U_ *=* 0, *γ* = 0.7, *μ* = 0.5, *D*_W_ = 1.2, *ρ* = 4.55, *k*_W1_ = 110, *W*_tot_ = 80, *a*_W_ = 4 |
| S8D Fig | *α* = 2, *β* = 3.5, *χ*_U_ *=* 0, *γ* = 0.5, *μ* = 0.5, *D*_W_ = 3, *ρ* = 5.56, *k*_W1_ = 90, *W*_tot_ = 80, *a*_W_ = 2.4 |
| S10A Fig | *α* = 10, *β* = 3.5, *χ*_U_ = 50, *γ* = 0.32, *μ* = 1.0, *θ* = 56, *d* = 0.8, *σ* = 0.02, *a*= 17.5, b=0.09 |

**Table P. Average and standard deviation of the hand-crafted features**

|  | **Features** | | |
| --- | --- | --- | --- |
|  | **Elongation parallel to front direction**  ***H*_1_**  **(*i*=1)** | **Elongation orthogonal to front direction**  ***H*_2_**  **(*i*=2)** | **Circularity**  ***H*_3_**  **(*i*=3)** |
| **Average (*σ_i_*)** | 27.7 | 29.7 | 0.82 |
| **Standard deviation (*μ_i_*)** | 9.3 | 7.7 | 0.21 |

**Table Q. Classification accuracy of snapshot images using hand-crafted features + LDA.**

| **Validation dataset** | **Predicted class** | | |
| --- | --- | --- | --- |
|  | ***Dictyostelium***  **(aggregation-stage)** | **HL-60** | **keratocyte** |
| ***Dictyostelium***  **(aggregation-stage)** | 52.1% | 32.4% | 15.5% |
| **HL-60** | 0.0% | 99.3% | 0.7% |
| **keratocyte** | 0.0% | 12.2% | 87.8% |

**Table R. Classification accuracy of snapshot images using hand-crafted features + SVM (linear kernel).**

| **Validation dataset** | **Predicted class** | | |
| --- | --- | --- | --- |
|  | ***Dictyostelium***  **(aggregation-stage)** | **HL-60** | **keratocyte** |
| ***Dictyostelium***  **(aggregation-stage)** | 54.4% | 39.9% | 5.7% |
| **HL-60** | 0.0% | 96.3% | 3.7% |
| **keratocyte** | 0.0% | 2.0% | 98.0% |

**Table S. Qualitative morphology features captured by related models.**

|  | Neilson *et al.*, 2011 [38] | Shi  *et al.*, 2013 [39] | Miao *et al.*, 2017 [14] | Bhattacharya *et al.*, 2020 [40] | Cao  *et al.*, 2019 [37] | Edelstein-Keshet  *et al.*, 2013 [41] | Alonso *et al.*, 2018 [42] | Moreno *et al.*, 2020 [53] | Our model |
| --- | --- | --- | --- | --- | --- | --- | --- | --- | --- |
| Canoe-like and high directional persistence | - | - | + | - | ++ | + | - | ++ | ++ |
| Elongated form with directional persistence | + | + | + | - | + | + | ++ | + | ++ |
| Pseudopod-like Y-split dynamics | ++ | + | + | + | - | - | + | + | ++ |
| Systematic comparison to real data | - | - | - | - | - | - | - | - | ++ |
| Pattern generating kinetics (E:excitability, B:bistability) | E | E | | | E | B | B | | E + B |
| Membrane deformation | 1D (level-set) | 1D (level-set) | | | 2D (phase-field) | 2D (phase-field) | 2D  (phase-field) | | 2D (phase-field) |

(++) Fully described, (+) subtle or intermediate forms, (-) not observed.

**Table T. Moreno et al model parameters in S11 Fig.**

| **Parameters** | **Values** |
| --- | --- |
| *σ* | 800, 2000, 4000, 6000, 8000 |
| *k*_a_ | 2, 3.5, 5 |
| *C*_0_ | 14, 28, 42, 56, 70, 84, 98 |

**Table U. Feature distance between the real cell data and the rank1 simulations.**

|  | Score-D(agg) rank1 | Score-H rank1 | Score-K rank1 |
| --- | --- | --- | --- |
| Moreno *et al.*, 2020 | 13229 | 1656 | 10961 |
| Ours | 4954 | 2715 | 18021 |
