# Supplementary figures and images for: Comparative mapping of crawling-cell morphodynamics in deep learning-based feature space
Source: PLoS Comput Biol. 2021 Aug 12;17(8):e1009237. doi: 10.1371/journal.pcbi.1009237 (PMC8360578; doi:10.1371/journal.pcbi.1009237)

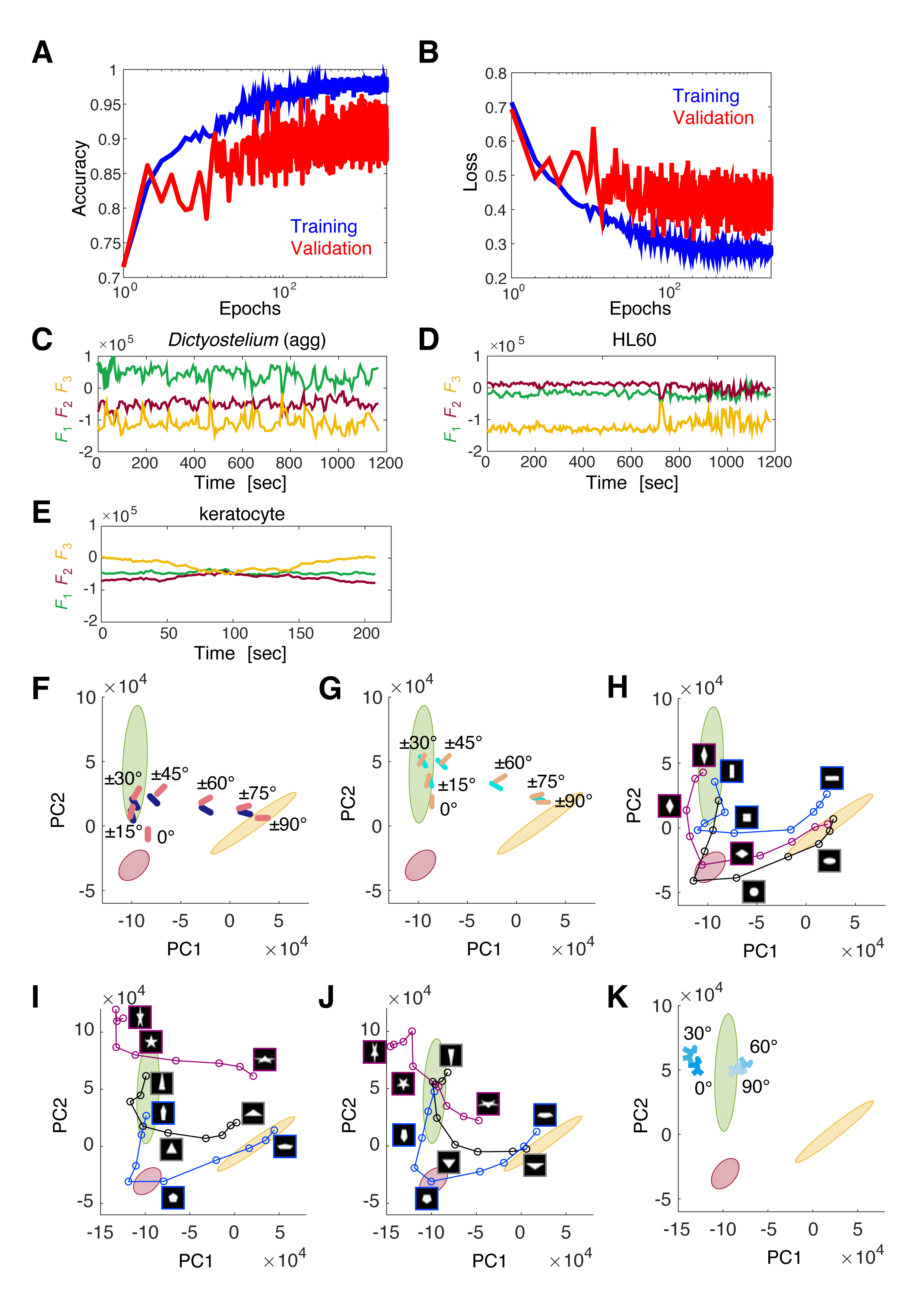

Supplement: S1 Fig — (A, B) The values of accuracy (A) and loss (B) during training (blue) and validation (red) of the deep convolutional neural networks. (C-E) Representative time series of the feature vector F for Dictyostelium (agg)(C), HL60 (D) and fish keratocyte (E). (F, G) Orientation dependency for the oval shape (Fig 1(M)) L = 0.75L0 (F) and L = L0 (G). (H-J) Mapping of circles and polygons with various aspect ratios (x-axis: y-axis) 1:3, 2:5, 1:2, 2:3, 1:1, 3:2, 2:1, 5:2 and 3:1. Circles, squares and rhombuses (H), triangles, pentagons and star shapes in the upright (I) and inverted (J) orientation. (K) Orientation dependency of a complex shape with multiple edges. (TIF) [file pcbi.1009237.s002.tif]

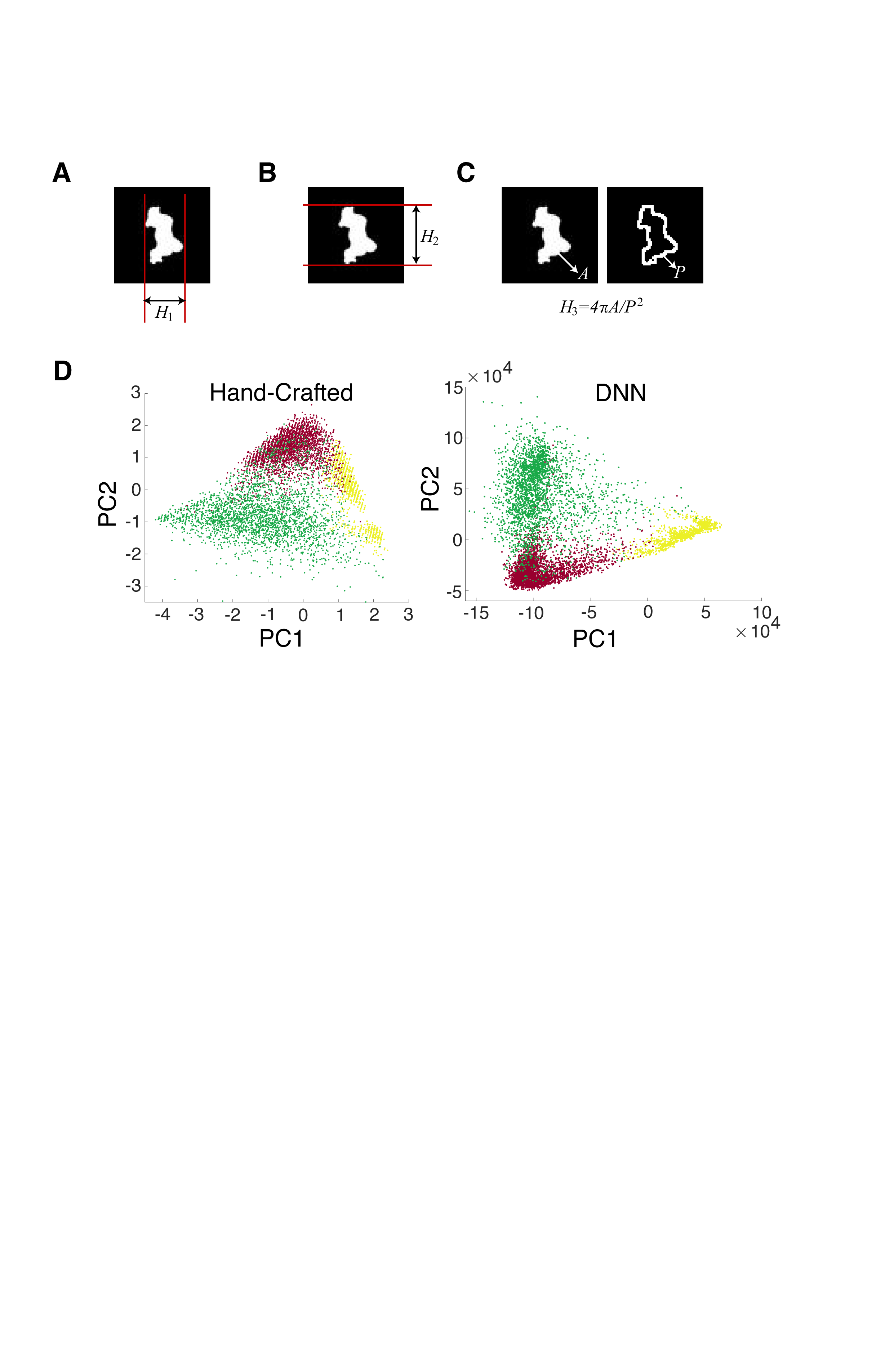

Supplement: S2 Fig — (A, B) Definition of h1 (A) and h2 (B). The first feature h1 and the second feature h2 is the degree of elongation parallel and orthogonal to the front-tail axis direction. Those values were extracted using the normalized mask image. (C) Definition of h3. The third feature h3 is circularity of a cell mask. (D) PC1-PC2 diagram obtained from each snapshots using hand-crafted features (left). The same diagram obtained using DNN-based features was also shown for comparison (right). The aggregation-stage Dictyostelium, HL-60, and fish keratocyte were shown as dark red, dark red, and yellow colors. (TIF) [file pcbi.1009237.s003.tif]

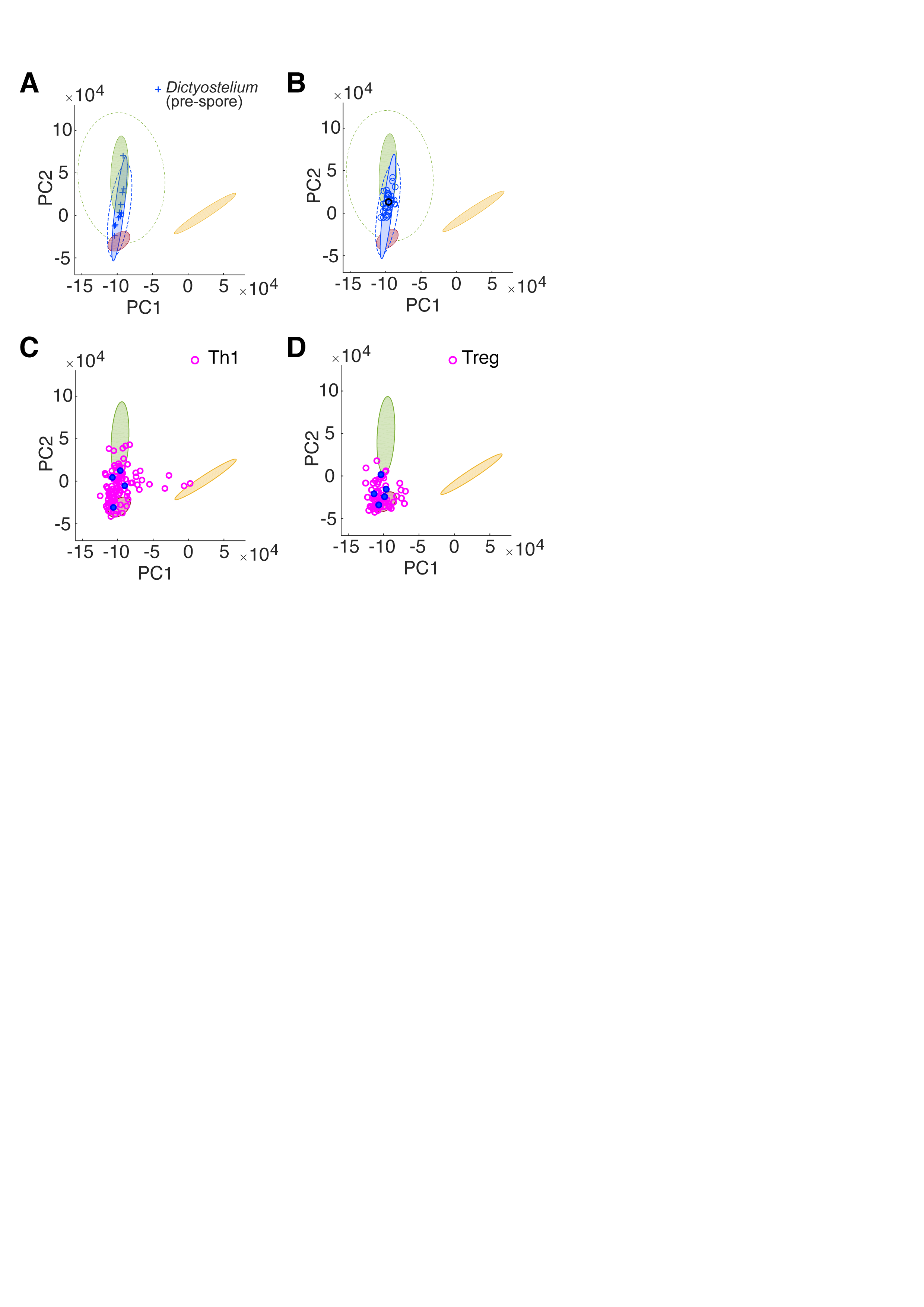

Supplement: S3 Fig — (A, B) Mapping of cell shape with anterior-posterior elongation without lateral pseudopods (Dictyostelium prespore cell-type). Cell-to-cell variation (A) and a representative temporal variation of a single cell (B). Blue circled regions represent 95% confidence eclipses for the mean of all combined timeseries (dotted) and the mean of individual cells (filled). (C, D) Mapping of mouse T cells in the PC1-PC2 space. Filled rhombuses: T helper 1 (C) and regulatory T cell (D). Circled regions in the background indicate the reference data in Fig 1B (aggregation-stage Dictyostelium dark green (dark green), HL60 (dark red) and fish keratocyte (yellow)). (TIF) [file pcbi.1009237.s004.tif]

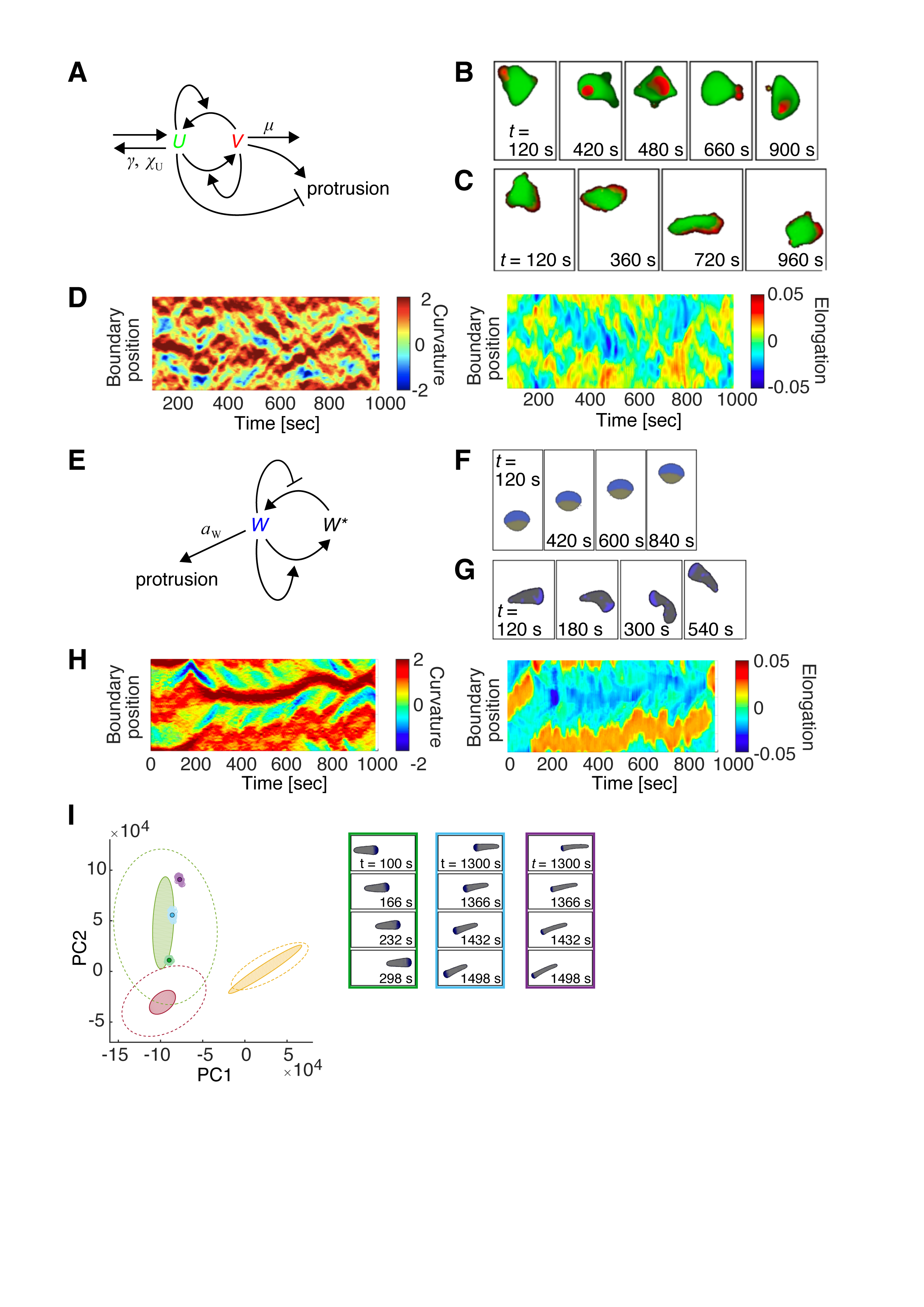

Supplement: S4 Fig — (A-D) The 2-variable scheme (A) and its morphology dynamics (B-D). Representative simulations showing traveling patches (B) and lamellipodium-like protrusions (C). Color overlay; red V, green U. (D) Time series of the local curvature of the boundary (left panel) and local elongation (right panel) for (C). (E-G) The 1-variable scheme (E) and its representative morphology dynamics (F, G). Blue indicates the high W region. (H) Time series of the local curvature at the boundary (let panel) and local elongation (right panel) for (G). (I) Feature mapping of the polar morphology in 1-variable scheme. Time average (solid circles) and time samples (filled). Right panels in colored frames indicate representative snapshots. See Table O in S1 Text for parameter values. (TIF) [file pcbi.1009237.s005.tif]

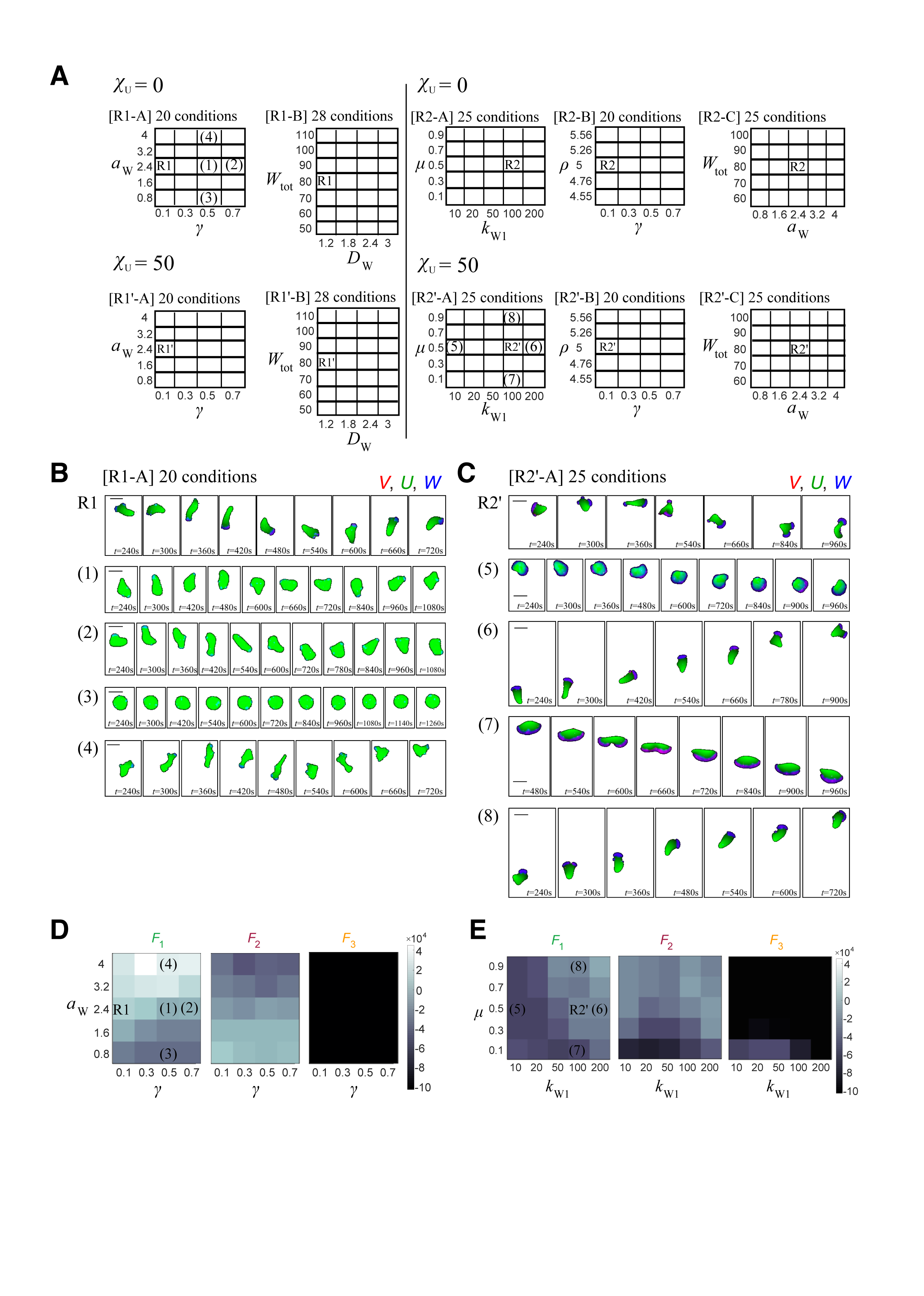

Supplement: S5 Fig — (A) Two-dimensional grids around manually selected reference parameters R1 (upper left panels; χU = 0), R1’ (lower left panels, χU = 50), R2 (upper right panels) and R2’ (lower right panels). Each square in the grid represents a sampled parameter condition. (B, C) Representative simulation time series for parameters R1, R2’ and (1)-(8) in (A). (D) Feature scores (F1, F2, F3) in the (γ-aW) plane around R1. (E) Feature scores (F1, F2, F3) in the (kW1-μ) plane around R2’. See Table O in S1 Text for parameter values. (TIF) [file pcbi.1009237.s006.tif]

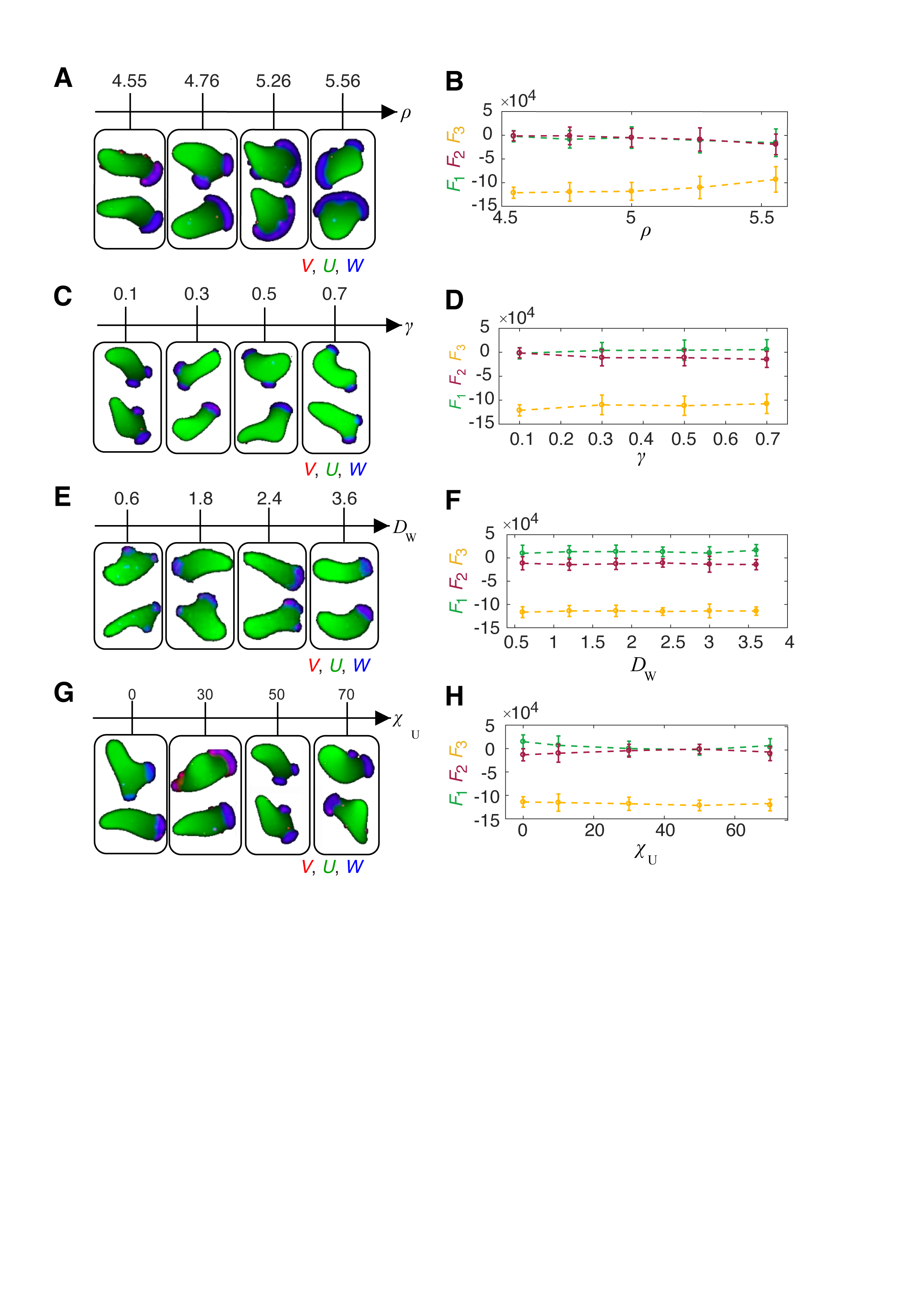

Supplement: S6 Fig — Dependency on γ, ρ, DW, χU of the simulated morphology (A, C, E, G) and the feature values (B, D, F, H). See Table O in S1 Text for parameter values. (TIF) [file pcbi.1009237.s007.tif]

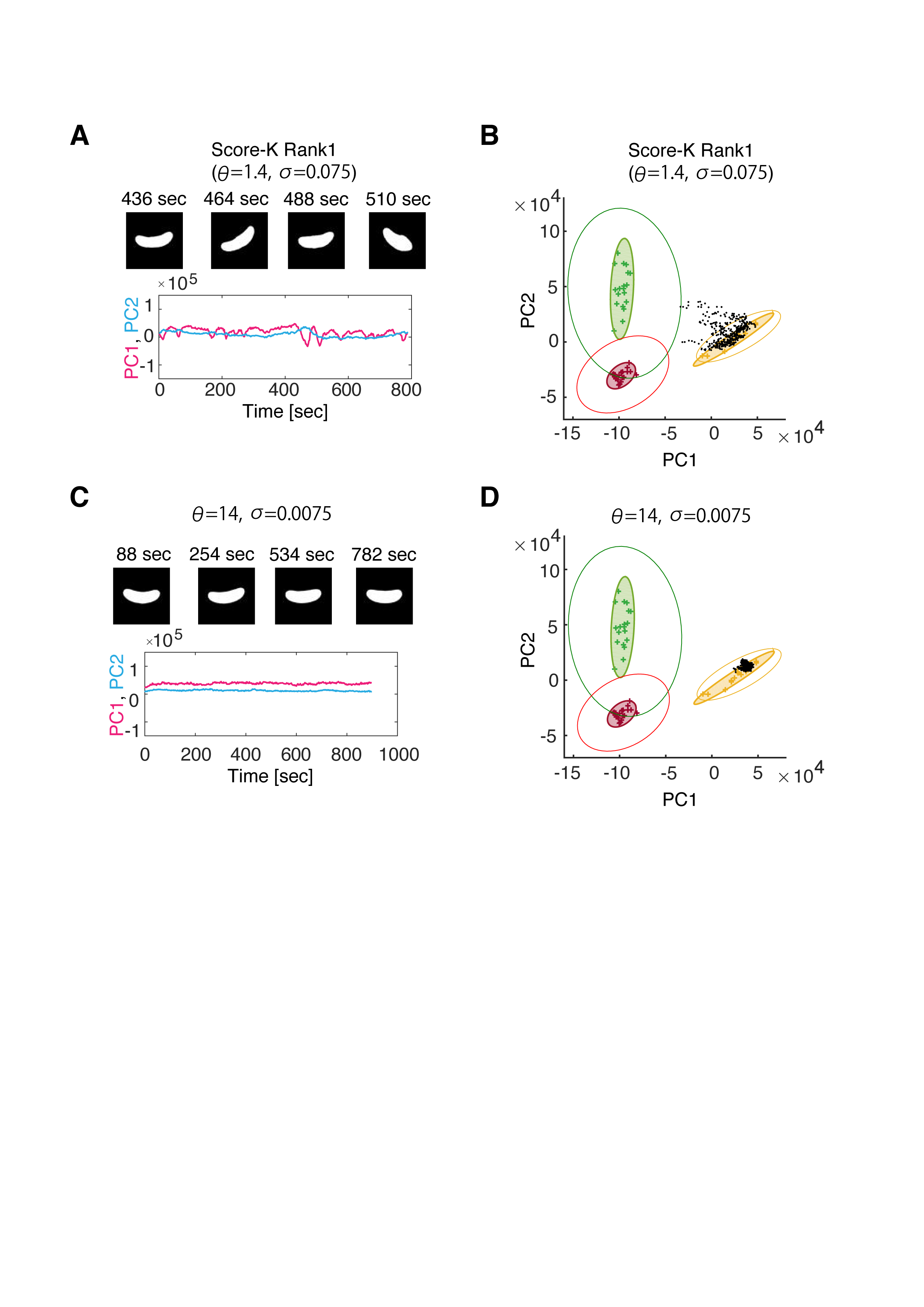

Supplement: S7 Fig — (A, B) Shape and orientation fluctuations observed under the default noise size (θ = 1.4, σ = 0.075) (A). Mapping in PC1-PC2 plane in Score-K rank1 simulation (B). (C, D) The same analyses at (θ = 14, σ = 0.0075). (TIF) [file pcbi.1009237.s008.tif]

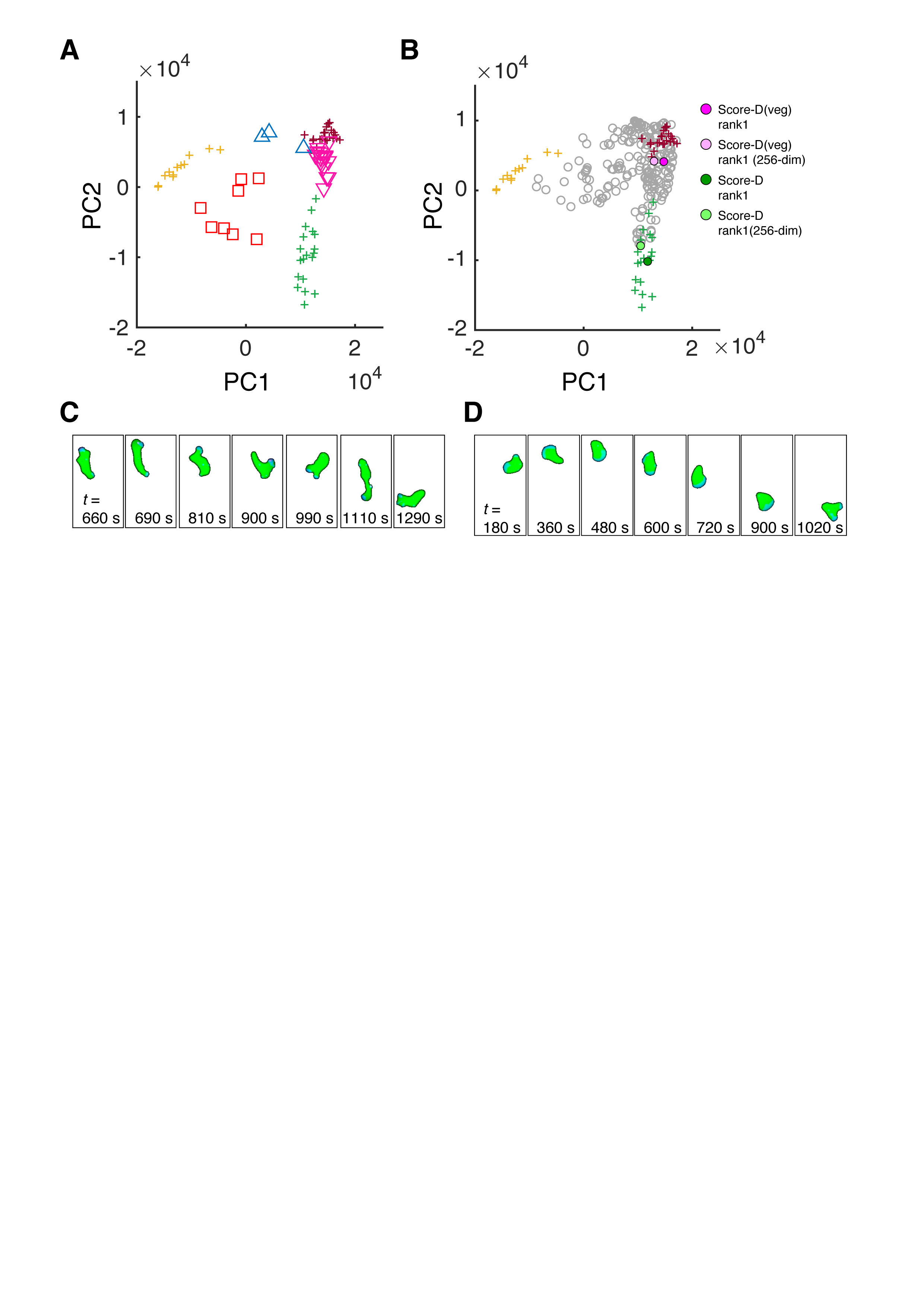

Supplement: S8 Fig — (A) PCA of the 256 dimensional intermediate layer. Microscopy dataset of Dictyostelium (agg) (green +), HL-60 (dark red +), keratocyte (yellow +), Dictyostelium (veg) (magenta inverted triangles), Dicytostelium racE- strain (red squares) and Nocodazole-treated HL-60 (blue triangles). (B) Mapping of model simulations (grey circles), the top-ranking simulations based on the feature vector F (dark green circle for Dictyostelium (agg), magenta circle for Dictyostelium (veg)) and those based on 256-dimensional features (light green circle for Dictyostelium (agg), pink circle for Dictyoste the lium (veg)). (C, D) Time-series of simulations with the highest feature similarity to Dictyostelium (aggregation-stage) (C), vegetative Dicyostelium (D). See Table O in S1 Text for parameter values. (TIF) [file pcbi.1009237.s009.tif]

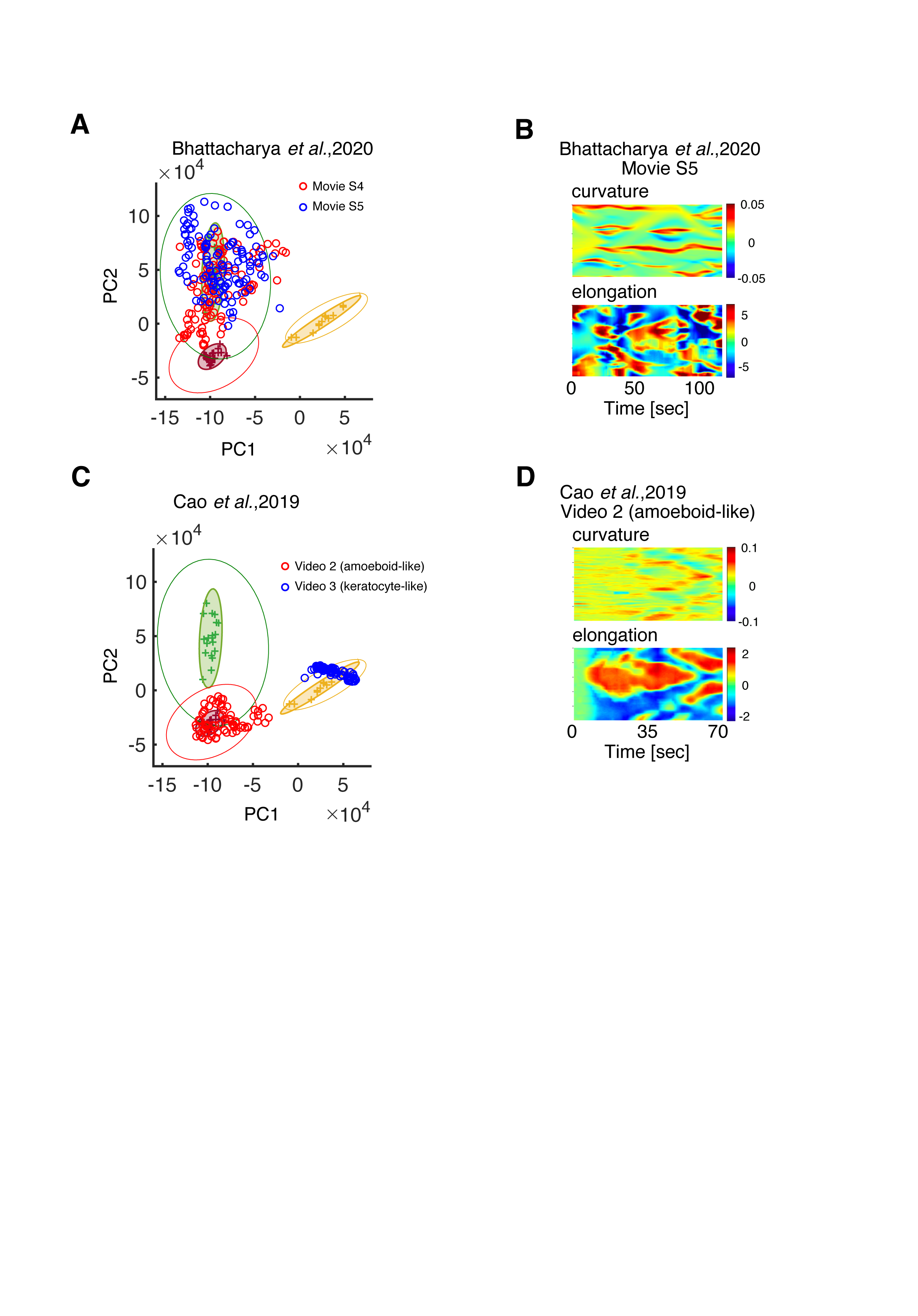

Supplement: S9 Fig — (A, B) The model by Bhattacharya et al [40]. The PC1-PC2 diagram was plotted based on snapshots taken from S4 Movie (red circle) and S5 Movie (blue circle) in [40] (A). Local boundary curvature (top) and the protrusion speed (bottom) obtained from S5 Movie in [40] (B). (C, D) The model by Cao et al., 2019 [37]. PC1-PC2 diagram was plotted based on snapshots from Video 2 (red circle) and Video 3 (blue circle) in [37] (A). Local boundary curvature (top) and the protrusion speed (bottom) obtained from Video 2 in [37] (B). (TIF) [file pcbi.1009237.s010.tif]

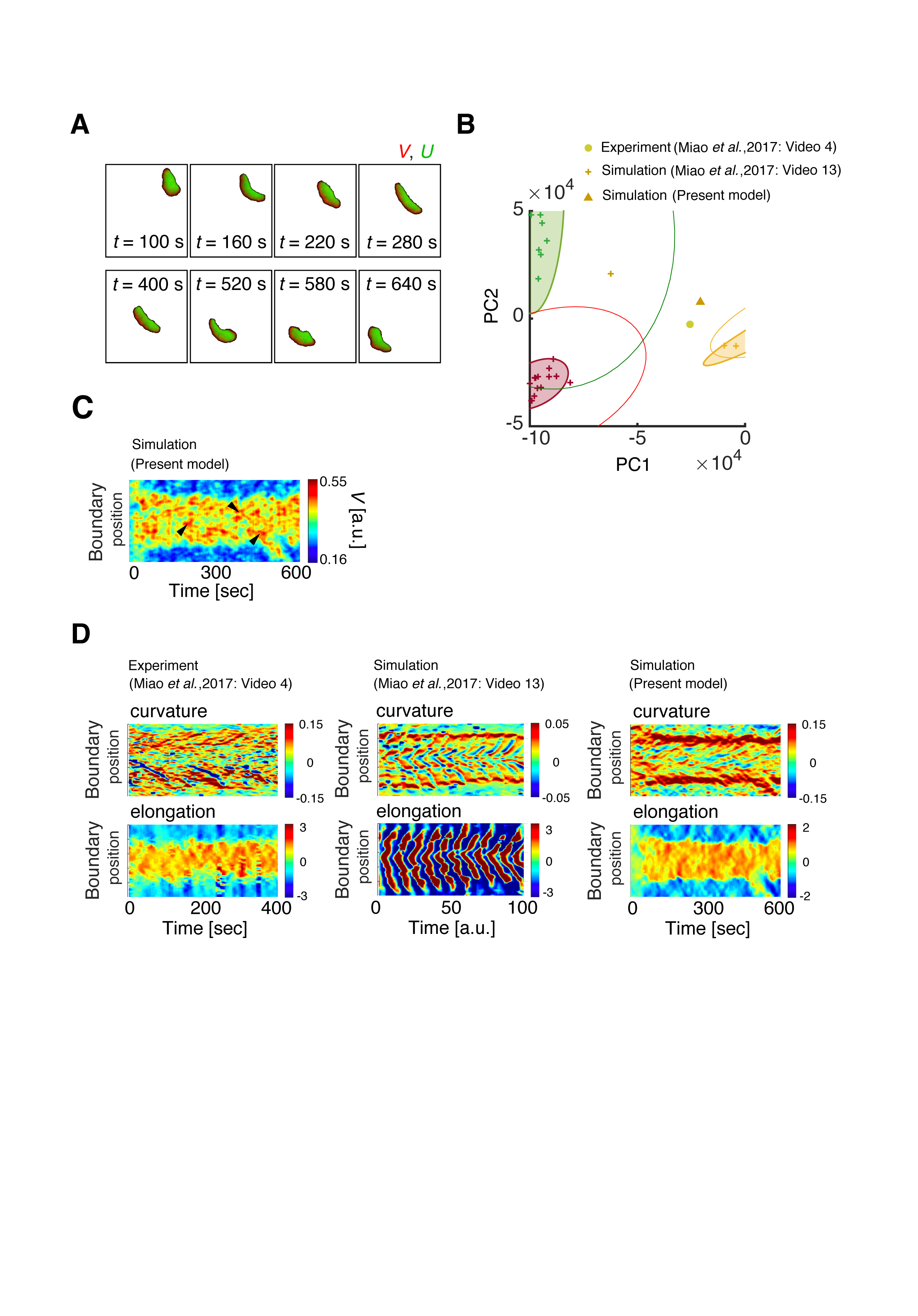

Supplement: S10 Fig — (A) A representative simulation result of a fan-shaped cell with a fluctuating front. Color overlay; red V, green U. (B) Feature mapping: present model (triangle), the PIP2-modulated cell (Video 4 in [14]) (circle) and an earlier model (Video 13 in [14]) (cross). (C) A kymograph of the variable V at the cell edge taken from the simulation in (A). Arrows indicate bifurcating V-shaped fronts. (D) Kymographs of the local curvature (top panels) and the protrusion size (bottom panels) along the cell boundary; for the real cell data (Video 4 in [14]) (left), and an earlier model simulation (Video 13 in [14]) (middle), and the present model (A) (right). (TIF) [file pcbi.1009237.s011.tif]

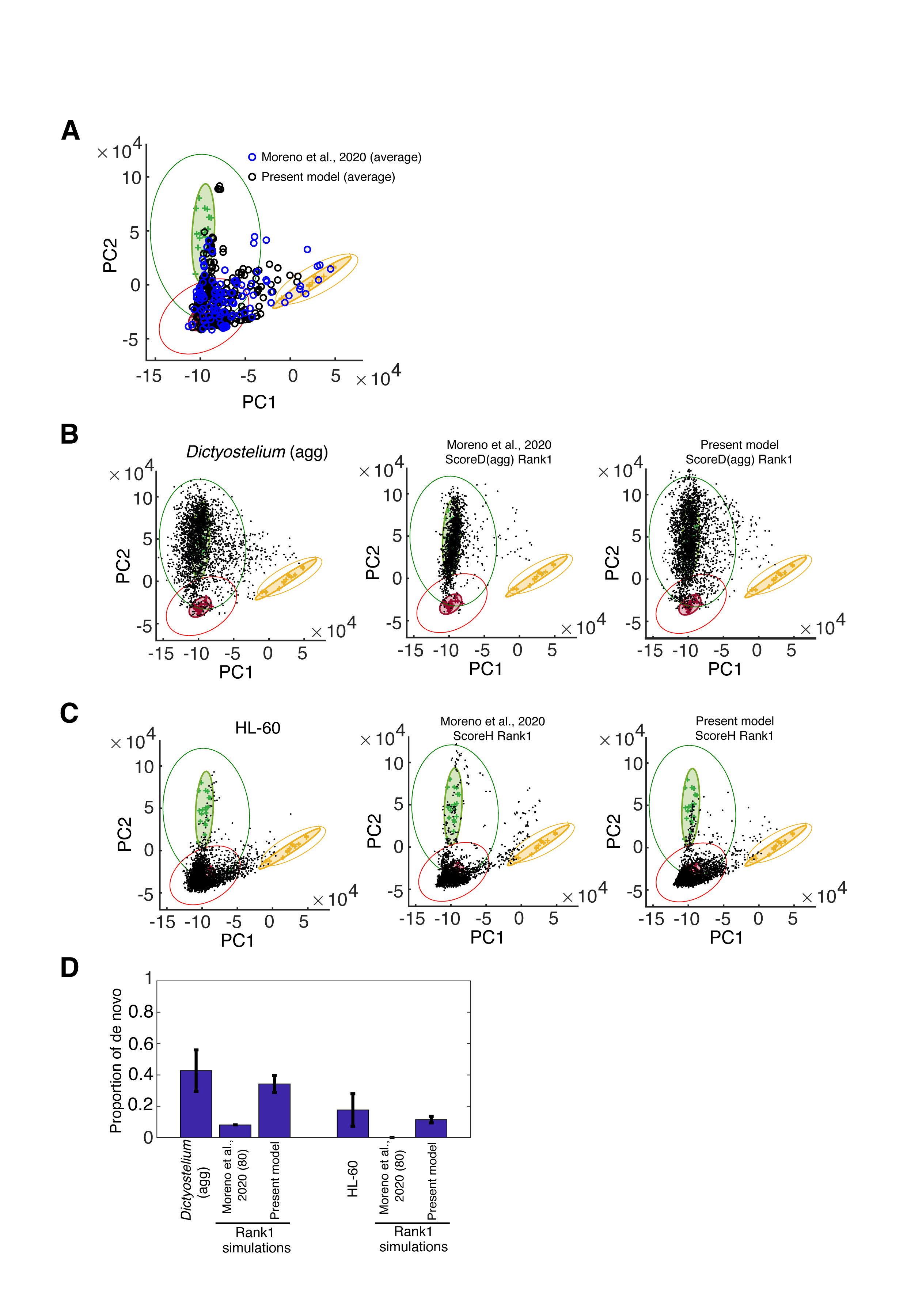

Supplement: S11 Fig — (A) Mapping of simulated cell morphology from 105 parameter sets of the model (blue circle). The results from our model (black circle) shown for comparison. (B) Mapping of individual snapshots of the Dictyostelium (agg) data (left), Score-D(agg) rank1 simulations in the Moreno et al. (middle) and our model (right). (C) Mapping of individual snapshots of HL-60 data (left), Score-H rank1 simulations in the Moreno et al. (middle) and our model (right). (D) Proportion of de novo pseudopod formation in real cell data and the rank1 simualtions; Dictyostelium (agg) (left lanes) and HL-60 (right lanes). (TIF) [file pcbi.1009237.s012.tif]
